# Polarclean/Water as a Safe and Recoverable Medium for Selective C2-Arylation of Indoles Catalyzed by Pd/C

**DOI:** 10.1021/acssuschemeng.0c05049

**Published:** 2020-10-27

**Authors:** Filippo Campana, Beatrice Maria Massaccesi, Stefano Santoro, Oriana Piermatti, Luigi Vaccaro

**Affiliations:** Laboratory of Green S.O.C. − Dipartimento di Chimica, biologia e Biotecnologie, Università degli Studi di Perugia, Via Elce di Sotto 8, 06123 Perugia − I, Italy

**Keywords:** polarclean/water, Pd/C, indole arylation, green solvents

## Abstract

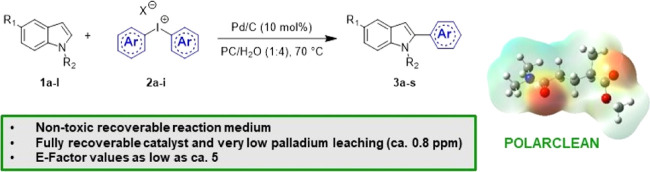

Herein, we report the use of nontoxic, water-miscible Polarclean as a safe dipolar aprotic solvent for the metal-catalyzed direct C2–H arylation of indoles using Pd/C as a catalyst. The developed method allows reaching excellent yields and regioselectivities, and it tolerates various substituents on both indole and diaryliodonium salt scaffolds. Polarclean is fully recoverable and reusable; it shows a very low leaching of the metal catalyst, allowing its complete recovery and reuse for at least six representative reaction runs.

## Abstract 2

A novel non-toxic reaction media for the selective Pd/C catalyzed C-2 arylation of indoles.

## Introduction

The choice of the reaction medium plays a major role in determining the efficiency of a synthetic procedure, as well as its sustainability profile.^1−4^ Classic polar aprotic solvents such as *N,N*-dimethylformamide (DMF), *N*,*N*-dimethylacetamide (DMA), and *N*-methyl-2-pyrrolidone (NMP) still find wide applications in synthesis, particularly in metal-catalyzed reactions, because they are generally very effective as reaction media. However, their use has been recognized as highly problematic from the point of view of sustainability, due to their toxicity and their origin from nonrenewable sources.^5−7^

Environmental, health, and safety concerns associated with the use of these solvents can be minimized by identifying alternative green solvents that may offer high chemical efficiency but also increase the sustainability of a process.^5−23^

Generally, an ideal green solvent is defined as a versatile, inexpensive, nontoxic, recyclable and/or biodegradable, and nonvolatile liquid with a sufficiently high boiling point to minimize exposure and release into the environment. A green solvent must be produced from renewable sources, e.g., from biomass via enzymatic fermentation/esterification processes.^8^

**Figure 1:**
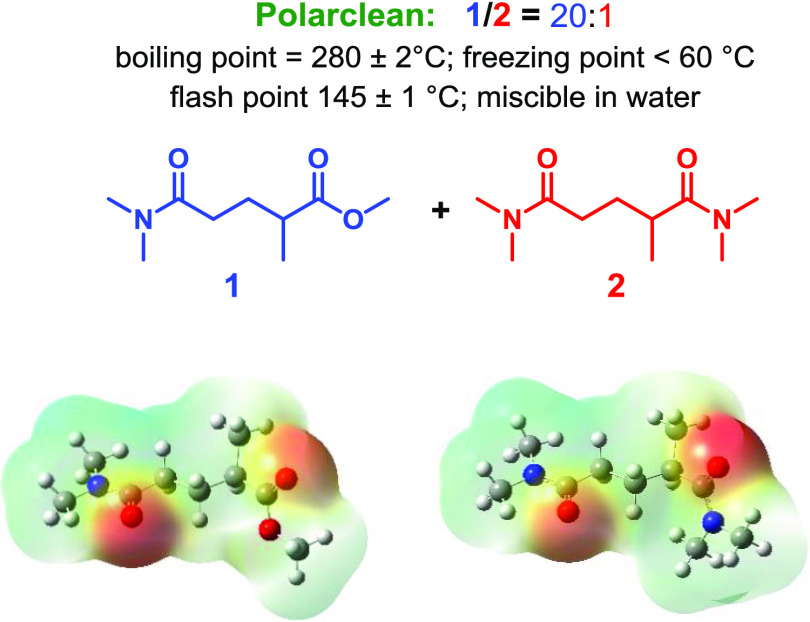
Structure and properties of Rhodiasolv® Polarclean.

However, green alternatives are not always available for all solvent classes. While a wide choice of green alcohols or low-polarity esters are available, there is only a limited number of green candidates with dipolar aprotic properties.^10^ Representatively, some examples include γ-valerolactone (GVL),^17−19^*N*-butylpyrrolidone (NBP),^20,21^ and Rhodiasolv® Polarclean,^22,23^ whose main component is methyl 5-(dimethylamino)-2-methyl-5-oxopentanoate (**1**) (Figure 1).

We have focused our attention on Polarclean (hereinafter referred to as PC), a promising solvent that can reduce carbon footprint as it is produced on a ton scale by Solvay in a circular economy approach. In fact, in large-scale production of Nylon 6,6,^24^ the key byproduct 2-methylglutaronitrile (MGN) is converted into the corresponding imide and then into dimethyl 2-methylpentanedioate (commercially known as Rhodiasolv® IRIS), the diester precursor of Polarclean.^22−24^

Recently, additional efforts have also been invested in the development of an alternative and “greener” synthetic route for producing Polarclean.^25^

Polarclean is miscible with water, possesses a high boiling point (bp 278–282 °C), is not flammable (fp 145 °C), is not toxic nor mutagenic, and is highly biodegradable.^26^ Thanks not only to its eco-friendly properties but also to its solvation ability, Polarclean finds one of its larger applications as a solvent or cosolvent for agrochemical formulation, crop protection, and animal nutrition.^26,27^

Its use as a reaction medium is limited to a few examples, i.e., polymerization,^28,29^ olefin epoxidation,^30^ nucleophilic aromatic substitution,^25^ and membrane fabrication.^31−35^

Recently, we have proved that the use of Polarclean as a reaction medium can be extended to some metal-catalyzed reactions opening to the environmentally safe and waste-minimized synthesis of heterocyclic systems.^36,37^ For example, 1,2,3-triazoles^36^ have been prepared in high yields by copper-catalyzed cycloaddition in Polarclean/water (4:1). This solvent system allowed isolating the desired heterocyclic products in very high yields by simple filtration. In addition, the reaction medium and the catalyst dissolved therein could be both recovered and successfully reused. Polarclean has also allowed the effective preparation of isoxazoles via copper-catalyzed dipolar cycloaddition, and it has been employed in a Pd(II)-catalyzed intramolecular C–H functionalization process for the preparation of heterofused triazoles.^37^

Direct functionalization of the nonactivated C–H bond represents an attractive synthetic strategy that can maximize atom and step economy providing modern and chemically sustainable alternative protocols for organic synthesis.^38−47^ In this context, the development of sustainable protocols for C–H functionalization processes based on the use of heterogeneous catalysis,^48^ and/or environmentally benign media,^19,49−51^ represents an important challenge.

Direct C–H functionalization of indoles is potentially of great synthetic utility, providing access to an important class of heterocyclic systems.^52−65^ In fact, an indole scaffold is considered as a privileged structure in medicinal chemistry, and it is present in a large number of natural products endowed with biological activity.^66−69^

While catalytic methods for direct C2–H arylation of indoles have been widely explored,^52−65^ to our knowledge, only a few reports have been disclosed on the use of heterogeneous Pd/C as a catalyst.^70−77^ Among them, two papers are based on the use of diphenyliodonium tetrafluoroborate in EtOH or water, which allowed the arylation of indole^70^ or *N*-methylindole,^71^ respectively. High C2 selectivity has been achieved although the yields of isolated products are moderate.^70,71^

Among green protocols, relevant are the results on the use of micellar aqueous catalysis to access milder conditions^61^ or Pd@MOF catalysts for the selective C2-arylation of *N*-methylindoles.^56^

Here, we report our investigation on the beneficial effects of Polarclean, a new, biodegradable, safe, and “industrial waste”-derived solvent as a reaction medium for C2-arylation of both free N–H and N-methylindoles using diaryliodonium salts as an arylating agent and commercial Pd/C as a catalyst, proving that this approach leads to a significant minimization of waste production in the preparation of C2-aryl-decorated indoles.

## Experimental Section

All chemicals were purchased and used without further purification. ^1^H NMR and ^13^C NMR spectra were recorded at 400 and 100.6 MHz, respectively, on a Bruker DRX-ADVANCE 400 MHz. Gas–liquid chromatography (GLC) analyses were performed using a Hewlett-Packard HP 5890A equipped with a DB-35MS capillary column (30 m, 0.53 mm), an FID detector, and helium as the gas carrier. Gas chromatography with electron impact mass spectrometry (GC-EIMS) analyses were carried out using a Hewlett-Packard HP 6890N Network GC system/5975 mass selective detector equipped with an electron impact ionizer at 70 eV. Pd leaching analyses were performed using an Agilent 4210 MP-AES instrument. Thin-layer chromatography analyses were performed with silica gel on aluminum plates (silica gel 60 F254, Fluka).

### Typical Procedure for Direct C–H Arylation of Indole (**1a**) with Diphenyliodonium Tetrafluoroborate (**2a**)

In a screw-capped vial equipped with a magnetic stirring bar, indole (**1a**) (0.2 mmol, 23.4 mg), Pd/C (10 mol %, 21.3 mg), 1 mL of PC/H_2_O (1:4), and diphenyliodonium tetrafluoroborate (**2a**) (0.25 mmol, 92 mg) were consecutively added, and the resulting mixture was left under stirring at 70 °C. After 4 h, the reaction mixture was left to cool to room temperature and then centrifuged to separate and recover the reaction medium, PC/H_2_O. The solid residue was then washed with hot ethyl acetate (2 × 0.5 mL), which was recovered as a supernatant after centrifugation (6500 rpm, 15 min). Recovered Pd/C was dried at 100 °C for 1 h and reused in the following run. The combined organic layers were concentrated under reduced pressure and the crude oil was purified by flash chromatography on silica gel (PE/EtOAc 9:1) to afford the title compound (**3a**) as a white solid (36 mg, 93%). Both the recovered reaction medium and the catalyst were reused for six consecutive runs.
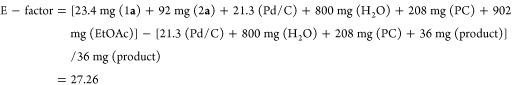


### Protocol with the Isolation of the Final Product by 2-Propanol Recrystallization (No Column Chromatography, Minimum 5 mmol)

In a screw-capped vial equipped with a magnetic stirring bar, indole (**1a**) (5 mmol, 585 mg), Pd/C (10 mol %, 532.1 mg), 25 mL of PC/H_2_O (1:4), and diphenyliodonium tetrafluoroborate (**2a**) (6.25 mmol, 2300 mg) were consecutively added, and the resulting mixture was left under stirring at 70 °C. After 4 h, the reaction mixture was cooled to room temperature and centrifuged to recover the reaction medium, PC/H_2_O. To the solid residue, 2-propanol (10 mL) was added, and the resulting mixture was left under stirring at 70 °C for 30 min. The catalyst was then filtered off, washed with additional hot 2-propanol (5 × 3 mL), and dried at 100 °C for 2 h before being reused for the following run. The combined organic layers were cooled to −20 °C for 24 h, and the pure recrystallized product (**3a**) was recovered in 83% yield (803 mg). Both the recovered reaction medium and the catalyst were reused for five consecutive runs. 2-Propanol used for recrystallization can be easily recovered (ca. 90%) by distillation.
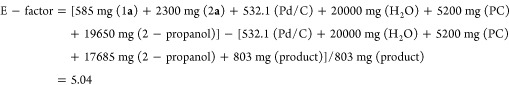


## Results and Discussion

**Figure 2:**
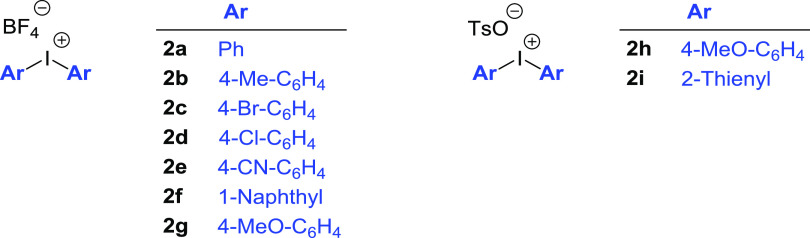
Diaryliodonium tetrafluoroborate and tosylate salts.

Diaryliodonium salts are hypervalent iodine compounds known for their stability, safety, and low toxicity and are widely used as selective arylating reagents for nucleophiles under metal-free and metal-catalyzed conditions.^78,79^ Diaryliodonium tetrafluoroborate **2a**–**2g** and diaryliodonium tosylate **2h** and **2i** used in this study are described in Figure 2.^80,81^

We initially used indole (**1a**) and diphenyliodonium tetrafluoroborate (**2a**) as representative substrates to test the catalytic efficiency of Pd/C in a series of green solvents (Table 1).

**Table 1:**
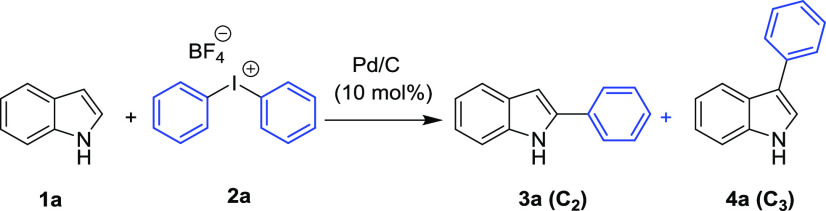
Screening of Green Solvents in Direct Arylation of Indole **1a** with Diphenyliodonium Tetrafluoroborate **2a**^a^

entry	medium	T (°C)	t (h)	conv. (%)^b^	C2/C3^b^	Yield (%)^c^
1^d^		90	4	>99	10:90	21^e^
2^d^	GVL	90	4	43	9:91	
3^f^	GVL	90	4	>99	90:10	41
4	GVL	90	4	98	95:5	46
5	GVL	70	15	>99	97:3	52
6	EtOH	70	15	>99	97:3	48
7	DMC	70	15	>99	57:43	40^g^
8	2-MeTHF	70	15	95	89:11	74^g^
9	*t*BuOMe	70	15	25	75:25	22^g^
10	CPME	70	15	51	73:27	36^g^
11	Polarclean	70	15	35	97:3	34^g^
12	Polarclean	90	8	96^h^	94:6	90
13	PC/H_2_O (4:1)	90	4	>99	92:8	90^g^
14	PC/H_2_O (3:2)	90	4	>99	94:6	93^g^
15	PC/H_2_O (3:2)	70	4	>99	95:5	95^g^
16	PC/H_2_O (1:4)	70	4	>99^i^	97:3	93^j^
17	H_2_O	70	4	>99	92:8	76
18^d^	PC/H_2_O (1:4)	70	4	0		

a Reaction conditions: **1a** (0.1 mmol), **2a** (1.25 equiv), Pd/C (10 mol %), and medium (0.5 mL).

b Conversion to products was determined by GLC and ^1^H NMR analyses.

c Yield of the isolated C2 product.

d No catalyst.

e Yield of the isolated C3 product.

f 5 mol % catalyst was used.

g Determined by ^1^H NMR analyses with trimethoxybenzene as internal standard.

h After 4 h, the conv. was 58%.

i After 2 h, the conv. was 76%.

j NMR yield was 96%. Abbreviations: GVL = γ-valerolactone, EtOH = ethanol, DMC = dimethyl carbonate, 2-MeTHF = 2-methyltetrahydrofuran, *t*BuOMe = *t*-butyl methyl ether, CPME = cyclopentyl methyl ether, and PC = Polarclean.

The reaction performed under solvent-free conditions in the absence of a catalyst resulted in complete conversion after 4 h at 90 °C. In this case, the C3-phenyl-substituted indole was obtained as the major product (C2/C3 = 10:90), but it was isolated in yield as low as 21% (Table 1, entry 1). This result confirmed the natural tendency of the process to yield the thermodynamically favored C3 product under thermal Pd-free conditions^82−86^ and also that both arylated indoles and the starting material are prone to some decomposition when the reaction is performed at high temperatures, due to the polymerization of indole moieties.^87,88^

When the reaction was performed in γ-valerolactone (GVL) as the medium, after 4 h at 90 °C, the conversion was only 43% and the C3-arylated product was predominant (entry 2). In the presence of 5 mol % Pd/C, the conversion was complete; C2-phenyl indole was obtained as a major product (C2/C3 = 90:10), but the yield of the isolated product was still as low as 41% (entry 3). Increasing the amount of Pd/C (10 mol %) resulted in an improvement in the selectivity (C2/C3 = 95:5) but with little increase in the yield of the C2-functionalized product (entry 4).

To improve the yields, temperature was tentatively reduced to 70 °C hoping that the uncatalyzed process could be slowed down. Accordingly, the reaction required 15 h to reach completion and the selectivity C2/C3 increased to 97:3. Unfortunately, thermal decomposition was still dominant and the isolated yield of the C2-arylated product only increased to 52% (entry 5).

Very similar results were obtained by performing the reaction in ethanol (entry 6); a decrease in selectivity was observed in dimethyl carbonate (DMC) and 2-methyl tetrahydrofuran (2-MeTHF), while a decrease of both reactivity and selectivity was observed in methyl *t*-butyl ether (*t*BuOMe) and cyclopentyl methyl ether (CPME) (entries 7–10). In all of the cases, the yield of the C2-functionalized product determined by ^1^H NMR analyses using 1,3,5-trimethoxybenzene as internal standard was much lower than the conversion measured by GLC analyses. Accordingly, the crude reaction mixture showed a significant amount of decomposition products.

Much better results were obtained when Polarclean (PC) was used as the reaction medium. After 15 h at 70 °C, the conversion was 35% and the C2/C3 ratio was 97:3, but the crude reaction mixture showed no signs of decomposition (entry 11). This behavior was somehow expected as it is known that arylated indoles undergo decomposition under thermal and acidic conditions.^87,88^ During the arylation of indoles, tetrafluoroboric acid is generated and therefore Polarclean, with its amide moieties, has a beneficial buffering role minimizing the decomposition of the product.

To confirm this beneficial effect, stability tests under thermal and acidic conditions were carried out on 2- and 3-phenylindole (**3a** and **4a**) in DMC and CPME in the absence or presence of dimethylacetamide to reproduce the effect of Polarclean (see Figure S1). In thermal conditions both in DMC and CPME, at 70 °C for 15 h, phenylindole **3a** and **4a** are stable and no appreciable decomposition is observed. In the presence of 1 equiv.. of HBF_4_, a non-negligible decomposition of 2-phenylindole **3a** was observed, while 3-phenylindole **4a** was stable in these conditions. When the test was performed in the presence of 1 equiv. of HBF_4_ and 5 equiv. of dimethylacetamide, decomposition of the products was completely avoided (see the Supporting Information, Figure S1).

By increasing the temperature to 90 °C, conversion to products was almost complete and the isolated yield of the desired C2-arylated product was 90% (entry 12).

Considering our previous experience and with the aim of simplifying the final workup procedure, the reactions were also performed in an increased amount of water added to Polarclean. As reported,^70−77^ water facilitates the process (entries 13–16), and when the mixture Polarclean/water (PC/H_2_O 1:4) was used, the arylation reaction reached completion in 4 h at 70 °C, with a 97:3 C2/C3 ratio and an isolated yield of the C2-functionalized product of 93% (entry 16).

Also, in pure water, the conversion was complete but both the selectivity and the yield of the isolated product were lower (entry 17).

Additionally, a kinetic study has been performed to confirm that C2-arylated indole is constantly the favored product when Pd(0) is used as a catalyst. As shown in Figure S2 (see the Supporting Information), the C2/C3 ratio remained constant throughout the reaction time.

When the reaction is performed in the absence of Pd/C, the thermal reaction is not effective at 70 °C (entry 18, Table 1); the formation of the C-3 product provides support for an electrophilic palladiation mechanism at C-3 followed by a 1,2-migration of palladium to the C-2 position that involves classic Pd(0)/Pd(II) catalytic cycles.^88−96^

The combination of water and Polarclean is not only beneficial for reactivity and selectivity but, from our experience, can be very effective in facilitating the isolation of products, as we reported in the waste-minimized synthesis of 1,2,3-triazoles.^36^

In fact, phenyl-substituted indoles **3a** and **4a** are insoluble in PC/H_2_O (1:4), and the use of this solution as a reaction medium allows the products to be recovered by simple decantation. Afterward, the products can be dissolved in a minimal amount of an extractive solvent (ethyl acetate or 2-propanol) and separated from the solid catalyst (Pd/C) by centrifugation. With this approach, both heterogeneous catalyst and medium are efficiently recovered.

To validate the experimental procedure, catalyst and reaction medium recycling has been investigated, and the results are reported in Table 2. For each reuse, the reaction medium was separated by centrifugation, the solid residue was washed with hot ethyl acetate (2 × 0.5 mL) to recover the products, and the Pd/C catalyst was separated by centrifugation, dried at 100 °C for 1 h, and reused in the next run. The solid catalyst and the reaction medium have been used for six consecutive runs without loss of activity and selectivity (Table 2).

**Table 2:**
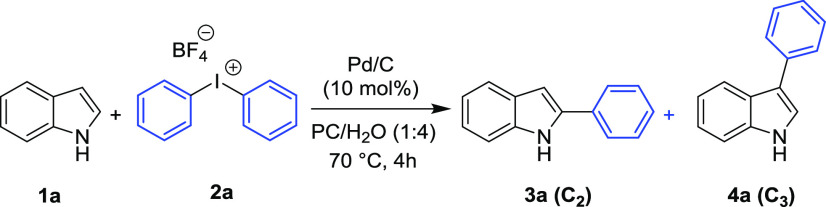
Recovery and Reuse of Both Reaction Medium and Catalyst for the Representative Direct Arylation of Indole **1a** with Diphenyliodonium Salt **2a**^a^

entry	conv. (%)^b^	C2/C3^b^	yield (%)^c^	Pd leaching (ppm)^d^
1	>99	97:3	93	0.8^e^
2	>99	97:3	93	0.9
3	>99	98:2	93	0.8
4	>99	98:2	92	0.8
5	>99	97:3	93	0.9
6	>99	96:4	92	1.0

a Reaction conditions: **1a** (0.2 mmol), **2a** (1.25 equiv), Pd/C (10 mol %), and PC/H_2_O (1:4, 1 mL) at 70 °C for 4 h.

b Conversion to products was determined by GLC and ^1^H NMR analyses.

c Yield of the isolated C2 product.

d Amount of palladium dissolved in the reaction medium was determined by MP-AES analyses.

e Pd leaching in the reaction medium after one cycle.

Looking more closely at the recycling study, it appears to be evident that at the end of the sixth run, in the reaction mixture, 6 equiv. of HBF_4_ as a side product is present as detected by boron MP-AES analysis conducted on the solvent, product, and catalyst. It has been confirmed that tetrafluoroboric acid remains mostly in the reaction medium (88%), while the remaining portion settles over the catalyst. To elaborate the role of the acid, an additional reaction in the presence of 1–5 equiv. of HBF_4_ was performed (Table 3).

**Table 3:**
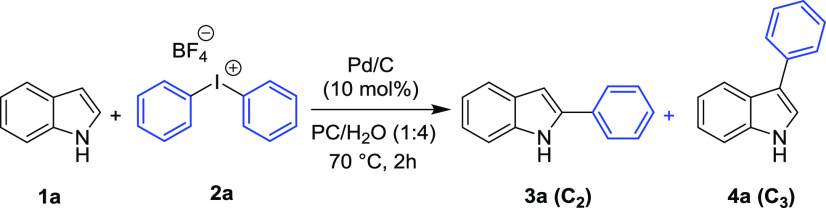
HBF_4_ Influence in Process Reactivity for the Representative Direct Arylation of Indole **1a** with Diphenyliodonium Salt **2a**^a^

entry	HBF_4_ (equiv)	conv. (%)^b^	C2/C3^b^
1	1	96	97:3
2	5	94	97:3

a Reaction conditions: **1a** (0.2 mmol), **2a** (1.25 equiv), Pd/C (10 mol %), and PC/H_2_O (1:4, 1 mL) at 70 °C for 2 h.

b Conversion to products was determined by GLC and ^1^H NMR analyses.

The data obtained (shown in Table 3) demonstrate that HBF_4_ influences the process reactivity by increasing the reaction rate but does not influence the product selectivity even when used in 1 or 5 equiv., simulating more than six consecutive runs.

This finding suggests that HBF_4_ could have a role in the catalyst particle size stabilization, as was reported for citric acid when used as a dispersing agent in the preparation of Pd/C. Indeed, the adsorption of citric acid on the Pd nanoparticle surface is considered the main reason for the enhanced catalytic activity of Pd/C.^89,90^

The amount of palladium dissolved during the reaction in the medium was quantified by MP-AES analyses, which showed very low metal concentrations after each reaction run (0.8–1.0 ppm), proving a very limited loss of palladium from the heterogeneous Pd/C catalyst, which can lead us to consider it as a stable catalyst for this process.

This behavior, as specified above, is probably enhanced by the presence of tetrafluoroboric acid, which could have a stabilizing effect on the catalyst.

At this stage, we also performed a hot-filtration test and a Hg-poisoning test to gain insights into the nature of the catalytically active species. In the optimized batch conditions, after 1 h, (a) 100 equiv (relative to the catalyst) of Hg(0) was added or (b) the hot reaction mixture was filtered to remove all heterogeneous components and the reactions were allowed to stir at the reaction conditions for an additional 3 h. No further reaction was observed in both cases, suggesting that no homogeneous active palladium species were present in the solution and so a plausible heterogeneous catalysis mechanism occurred (see the Supporting Information for details).^70,71^

Additional insights into the reaction mechanism and the actual role of the catalyst were obtained by performing the arylation of indole (**1a**) in the absence of Pd/C but also using the recycled PC/H_2_O (1:4) solution, without further addition of Pd/C (see the Supporting Information, Table S1). In all of the cases, arylation of **1a** performed in the absence of Pd/C in Polarclean, in water, or in the Polarclean/water (1:4) mixture at 70 °C for 4 h gave no traces of the arylation product. When the process was performed in the Polarclean/water (1:4) mixture recovered after the first or sixth run, no traces of products were detected, additionally proving that the amount of palladium released in the medium is minimal and not sufficient to promote the process.

**Scheme 1:**
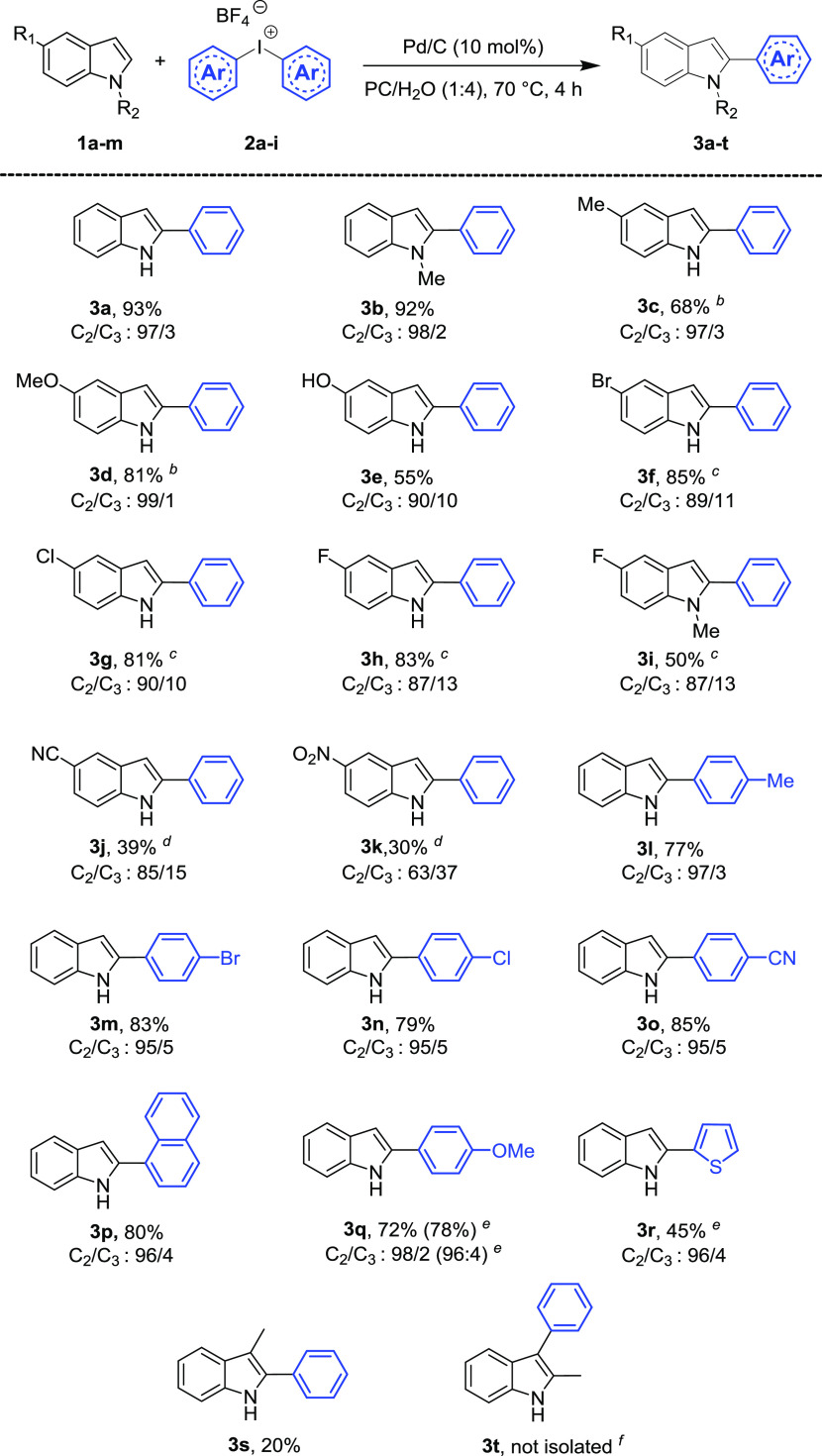
Substrate Scope for the Synthesis of Aryl Indoles Reaction time 8 h. Reaction time 15 h. Reaction performed at 120 °C. TsO^–^ as a counterion of **2**. Conv. (%) = 15%. Reaction conditions: **1** (0.2 mmol), **2** (0.25 mmol), and PC/H_2_O (1:4, 1 mL), 70 °C, 4 h; yield of isolated C-2 product **3**.

The scope and limitations of our protocol were therefore investigated by expanding the substrate scope to several substituted indoles (**1b**–**1e**) and a variety of diaryliodonium tetrafluoroborate (**2b**–**2g**) and tosylate (**2h**–**2i**) salts (Scheme 1).

*N*-Methyl indole (**1b**) showed a reactivity comparable to indole **1a**, giving the C2-arylated product in excellent isolated yield (92%). On the other hand, 5-substituted indoles showed a reactivity and selectivity strongly dependent on the nature of the substituent. Arylation of highly reactive 5-hydroxy indole (**1e**) is complete after 4 h at 70 °C, but the yield of the isolated product is only 55%, due to some product decomposition. 5-Methyl and 5-methoxy indoles (**1c** and **1d**, respectively) required a slightly longer reaction time for reaching complete conversion (8 h). For indoles possessing electron-withdrawing groups, such as 5-bromo, 5-chloro, and 5-fluoro (**1f–1i**), 15 h and a temperature of 70 °C were necessary for complete conversion to the corresponding arylated compounds. 5-Fluoro indole (**1h**) and *N*-methyl-5-fluoro indole (**1i**) showed the same selectivity but the isolated yield of the C2-functionalized product was lower for the latter. Indoles possessing strongly electron-withdrawing groups, such as 5-cyano indole (**1j**) and 5-nitro indole (**1k**), showed a satisfactory reactivity only at 120 °C (conversion to products being 75 and 80%, respectively). In both cases, C2/C3 selectivity (85:15 and 63:37, respectively) and yields (39 and 30%, respectively) were moderate. Longer reaction times did not allow better results due to the competitive thermal decomposition of the products.

The protocol showed good results also in the arylation of indole (**1a**) with diaryliodonium tetrafluoroborate salts **2b**–**2g** and tosylate salts **2h**–**2i** (Scheme 1).

In general, the reaction of diaryliodonium tetrafluoroborate salts **2b**–**2f** with indole **1a** provided, after 4 h at 70 °C, the C2-arylation products **3l**–**3p** with good to excellent selectivities and isolated product yields (77–85%). The bis-(4-methoxyphenyl)iodonium tetrafluoroborate **2g** and the corresponding tosylate **2h** showed a similar reactivity, but a slightly higher C2 selectivity was achieved with the tetrafluoroborate salt (C2/C3 = 98:2 vs 96:4). When bis(2-thiophenyl)iodonium tosylate **2i** was used, still at 70 °C for 4 h, the conversion observed was only 50% with a yield of isolated product **3r** of 45%. A longer reaction time or a higher reaction temperature did not lead to any improvement in the results. The reaction of 3-methyl indole (**1l**) with diphenyliodonium tetrafluoroborate (**2a**) at 70 °C for 4 h provided the corresponding 3-methyl-2-phenyl indole (**3s**) with a conversion of 60% and a yield of isolated product of only 20%. In the same conditions, 2-methyl indole (**1m**), in which C3–C2 migration was not possible, showed a lower reactivity and 2-methyl-3-phenyl indole (**3t)** was obtained with a conversion of only 15%.

Finally, with the optimized protocols, we also calculated the E-factors associated with our procedure and compared them to those reported in the literature (see the Supporting Information for details, p. S7–S11).

It is noteworthy that all of the protocols for the C2-arylation of indoles require the final classic chromatographic purification for the isolation of pure products. In most of the cases, the reaction medium and/or catalyst cannot be recovered. Therefore, we have initially calculated the E-factor values (kg of waste/kg of product) for our protocol based on the recovery of the reaction medium and the use of EtOAc. With this approach, excellent results have been obtained and both catalyst and reaction medium can be recovered and reused.

**Figure 3:**
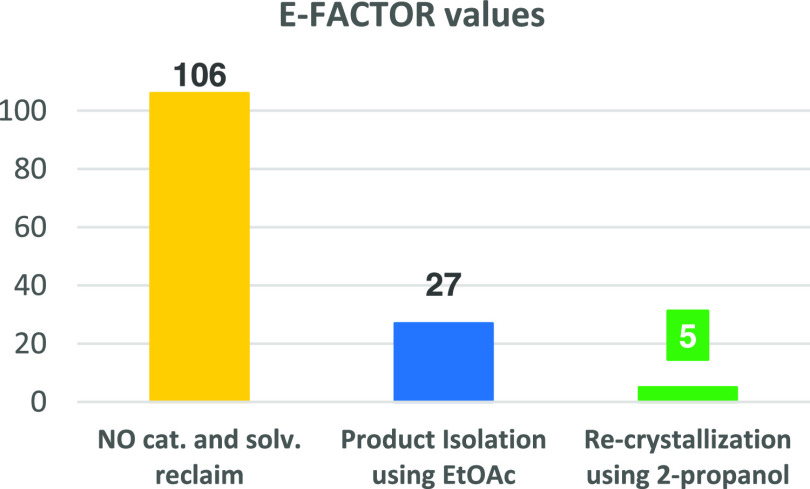
Graphical representation of the E-factor.

The calculated E-factor is 27 (blue bar in Figure 3), which is quite low compared to those calculated for literature protocols (66–105). This great improvement is essentially due to the recovery of the reaction medium and the heterogeneous catalyst. In fact, without considering this possibility, the E-factor value increases to ca. 106 (yellow bar). However, additional waste-producing column chromatography is needed in all cases. Therefore, to better exploit the recoverability of our reaction medium and catalyst, we have also investigated the possibility of avoiding the chromatographic purification.

We have found that hot 2-propanol can efficiently dissolve the products and induce their recrystallization at room temperature. This approach (see details in the Experimental Section), combined with the solvent and catalyst recovery, gives a very low E-factor value of 5 (green bar, Figure 3, see the Supporting Information for detailed calculations).

**Figure 4:**
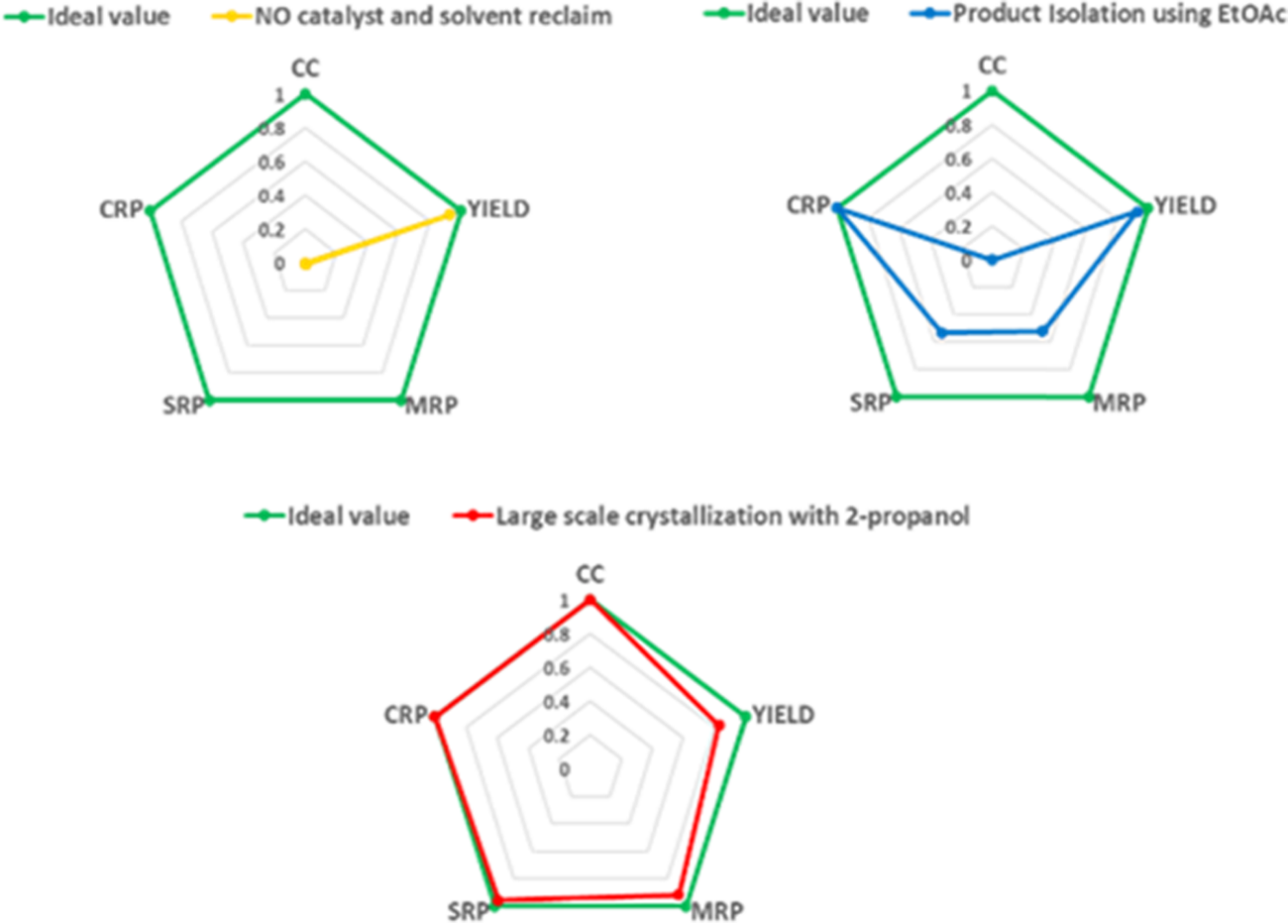
Radial polygon representation of green metrics.

Finally, to gain a better understanding of the contribution of different parameters to the sustainability of our methodology, we resorted to a graphical representation of radial polygons comprising different aspects of each process, such as the reaction yield, total mass recovery parameter (MRP), solvent recovery parameter (SRP), catalyst recovery parameter (CRP), and the use of column chromatography (CC) (Figure 4). It is evident that the significant contribution of the recovery/reuse approach of the reaction medium (mainly) and of the heterogeneous catalyst is responsible for most of the reduction in waste generation and therefore of the E-factor values of our classic protocol. Further optimization is derived using 2-propanol for the isolation of the product without the need for column chromatography. This almost allows reaching the ideal values in the radial pentagon representation. In this last case, the results refer to a minimum 5 mmol scale that facilitates the recrystallization step. On a larger scale, the results can presumably be even better.

## Conclusions

In this contribution, we investigated the use of Polarclean as a green and effective medium for the arylation of indoles. We found that optimal results can be achieved in the C2–H arylation of indoles using Pd/C as a heterogeneous catalyst and a mixture of Polarclean/water (1:4) as a reaction medium.

The procedure tolerates various substituents on both indole and diaryliodonium salt moieties, and it allows a simple separation of the products and the recovery and reuse of both the catalyst and the reaction medium for several runs without loss of activity and selectivity. Noteworthily, the best results are obtained using the combination of Polarclean and water, while their separate usage leads to less chemically and environmentally efficient protocols.

Most importantly, Pd/C is very stable in the adopted conditions, showing a very small loss of the metal in solution and therefore complete recoverability and reusability.

Besides the recoverability of the catalyst, green metrics calculations support the results obtained by showing that the waste generation associated with the Polarclean/water protocol is very small. In fact, the E-factor has been reduced from ca. 100, which is the average of the protocols reported in the literature (not considering column chromatography), to ca. 5. In addition, thanks to the recoverability of both catalyst and medium, it is also possible to define a gram-scale procedure allowing the recovery of the product by simple recrystallization and avoiding the classic wasteful column chromatographic purification.

## Supporting Information Available

Typical procedures; E-factor calculations; full characterization for all of the compounds; and copies of the ^1^H and ^13^C NMR spectra (PDF)
