# Supplementary material for: Polarclean/Water as a Safe and Recoverable Medium for Selective C2-Arylation of Indoles Catalyzed by Pd/C
Source: ACS Sustain Chem Eng. 2020 Oct 27;8(44):16441–50. doi: 10.1021/acssuschemeng.0c05049 (PMC8018292; doi:10.1021/acssuschemeng.0c05049)
Supplement: Supplementary file 1 — sc0c05049_si_001.pdf [file sc0c05049_si_001.pdf]

SUPPORTING INFORMATION

Polarclean/water as a safe and recoverable medium for the selective C2-arylation of indoles catalyzed by Pd/C

Filippo Campana, Beatrice Maria Massaccesi, Stefano Santoro, Oriana Piermatti, and Luigi Vaccaro**

Laboratory of Green S.O.C. – Dipartimento di Chimica, Biologia e Biotecnologie, Università degli Studi di Perugia, Via Elce di Sotto 8, 06123 – Perugia – I; E-mail: luigi.vaccaro@unipg.it; Web: www.dcbb.unipg.it/greensoc.

Table of Contents:

1. General Remarks SI 2
2. General Procedures and additional data SI 2-6
3. E-Factor calculations SI 7-11
4. Charts for Characterization Data of all the prepared compounds SI 12-30
5. Copies of the ^{1}H and ^{13}C NMR Spectra of all the prepared compounds SI 31-70

Total number of pages:70

Total number of tables: 5 (SI 3, SI 6, SI 11)

Total number of figures: 4 (SI 4, SI 5, SI 7, SI 10, SI 31-70)

General Remarks

All chemicals were purchased and used without any further purification. ^{1}H-NMR, ^{13}C-NMR spectra were recorded at 400 MHz and 100.6 MHz, respectively, on a Bruker DRX-ADVANCE 400 MHz. GLC analyses were performed by using the Hewlett-Packard HP 5890A equipped with a capillary column DB-35MS (30 m, 0.53 mm), an FID detector and helium as the gas carrier. Gas Chromatography with Electron Impact Mass Spectrometry (GC-EIMS) analyses were carried out by using a Hewlett-Packard HP 6890N Network GC system/5975 Mass Selective Detector equipped with an electron impact ionizer at 70 eV. Pd analyses were performed by using an Agilent 4210 MP-AES instrument. Thin layer chromatography analyses were performed with silica gel on aluminum plates (silica gel 60 F254, Fluka). Column chromatography purification was performed with silica gel (230–400 mesh) eluting with hexane/ethyl acetate (9:1–6:4).

Typical procedure for the direct C-H arylation of indole (1a) with diphenyliodonium tetrafluoroborate (2a)

In a screw capped vial equipped with a magnetic stirrer, indole (1a) (0.2 mmol, 23.4 mg), Pd/C (10 mol%, 21.3 mg), 1 mL of PC/H_2O (1:4) and diphenyliodonium tetrafluoroborate (**2a**) (0.25 mmol, 92 mg) were consecutively added and the resulting mixture was left under stirring at 70°C. After 4 h the reaction mixture was left to cool to room temperature, then centrifuged to separate and recover the medium, PC/H_2O. The solid residue was then washed with hot ethyl acetate (2x0.5 mL), which was recovered as supernatant after centrifugation (6500 rpm, 15 min). Recovered Pd/C was dried at 100 °C for 1h and reused in the following run. The combined organic layers were concentred under reduced pressure and the crude oil purified by flash chromatography on silica gel (PE/EtOAc 9:1) to afford the title compound (**3a**) as a white solid, 36 mg (93%).
Both the recovered reaction medium and catalyst were reused for six consecutive runs.

Protocol with the isolation of final product by 2-propanol re-crystallization (no column chromatography), (minimum 5 mmol).

In a screw capped vial equipped with a magnetic stirrer, indole (**1a**) (5 mmol, 585 mg), Pd/C (10 mol%, 532.5 mg), 25 mL of PC/H_2O (1:4) and diphenyliodonium tetrafluoroborate (**2a**) (6.25 mmol, 2300 mg) were consecutively added and the resulting mixture was left under stirring at 70°C. After 4 h the reaction mixture was cooled to room temperature and centrifuged to recover the reaction medium, PC/H_2O. To the solid residue, 2-propanol (10 mL) was added and the resulting mixture was left under stirring at 70 °C for 30 min. The catalyst was then filtered off, washed with additional hot 2-propanol (5x3 mL) and dried at 100 °C for 2 h before being reused for the following run. The combined organic layers were cooled to -20 °C for 24 h and the pure product re-crystallized was recovered in 83% yield (803 mg).
Both the reaction medium and catalyst recovered were reused for six consecutive runs.
90% of 2-propanol can be easily recovered by distillation.

Preparation of the samples for MP-AES analysis.

An aliquot (150 µL) of the reaction solvent, after separation from the heterogeneous catalyst was dissolved in 2 mL aqua regia and stirred for 1h at room temperature. Milli-Q water was added to reach a final volume of 10 mL. If present, residual solid was filtered off and the sample was analyzed by MP-AES 4210 instrument.

Table S1. Direct arylation of indole **(1a)** with diphenyliodonium salt (**2a)** without Pd/C catalyst at 70 °C for 4 h.

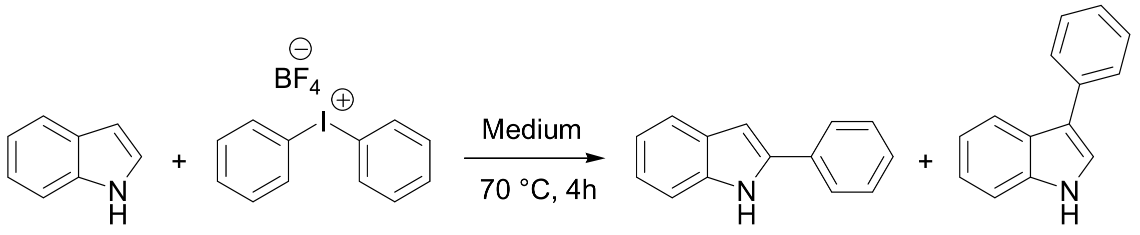

1a **2a** **3a (C2)** **4a (C3)**

Entry[a]	Medium	Conv. (%)[b]	C2 / C3[b]
1	Polarclean	0	-
2	PC/H_2O (4:1)	0	-
3	PC/H_2O (1:4)	0	-
4	H_2O	0	-
5	Medium recovered after one run	0	-
6	Medium recovered after six runs	0	-

[a] Reaction conditions: **1a** (0.2 mmol), **2a** (1.25 eq), PC/H_2O 1:4 (1mL) at 70 °C for 4 h. [b] Determined by GLC and ^{1}H-NMR analyses.

Table S2. TON and TOF calculation for the direct C2 arylation of NH-indole and comparison with other works.

	**TON**	**TOF (h^{-1})**
**This work**	46.5 (5 cycle)	2.3
Angew.Chem. Int. Ed. **2014**, *53*, 1809 –1813	8	0.36
ACS Catal., **2016**, *6*, 1062−1074, N-methyl indoles	98	8.2
Chem. Eur. J. ***2014***, *20*, 13531 – 13535	36.4	6.1

Figure S1. Stability test for 2-phenylindole **3a** and 3-phenylindole **4a** in DMC and CpOMe at 70 °C for 15 h in presence of HBF_4 and dimethylacetamide.

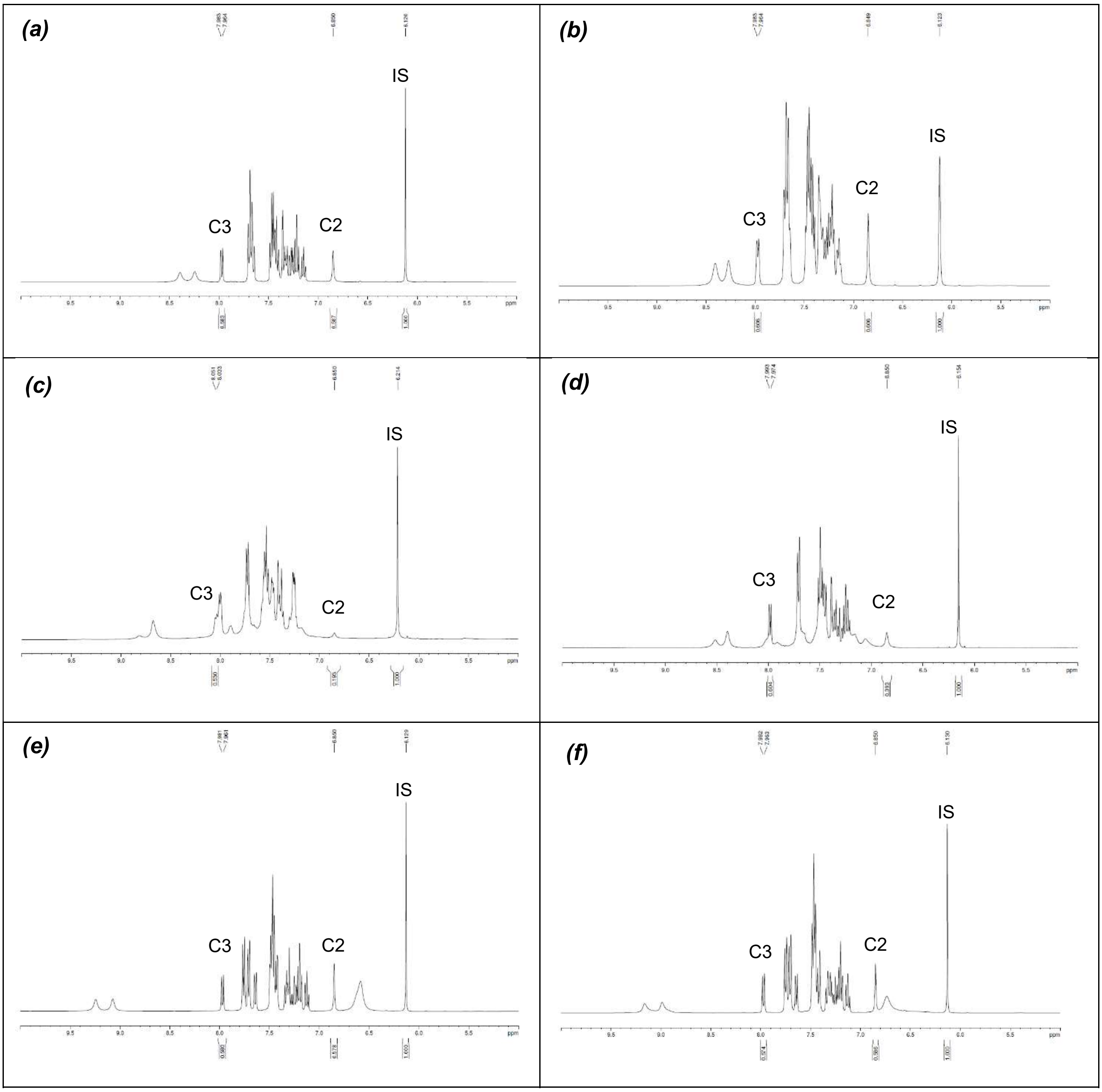

Conditions 1: 0.05 mmol of **3a**, 0.05 mmol of **4a**, 0.03 mmol of trimetoxybenzene as Internal Standard in 0.25 ml of DMC ***(a)*** or CpOMe ***(b)*** at 70 °C for 15 h.

Conditions 2: 0.05 mmol of **3a**, 0.05 mmol of **4a**, 0.05 mmol HBF_4, 0.03 mmol of trimetoxybenzene as Internal Standard, in 0.25 ml of DMC ***(c)*** or CpOMe ***(d)*** at 70 °C for 15 h.

Conditions 3: 0.05 mmol of **3a**, 0.05 mmol of **4a**, 0.05 mmol HBF_4, dimethylacetamide (5 equiv.), 0.03 mmol of trimetoxybenzene as Internal Standard, in 0.5 ml of DMC ***(e)*** or CpOMe ***(f)*** at 70 °C for 15 h.

Figure S2. Kinetic study of the reaction between indole (**1a**) and diphenyliodonium tetrafluoroborate (**2a**)[a]

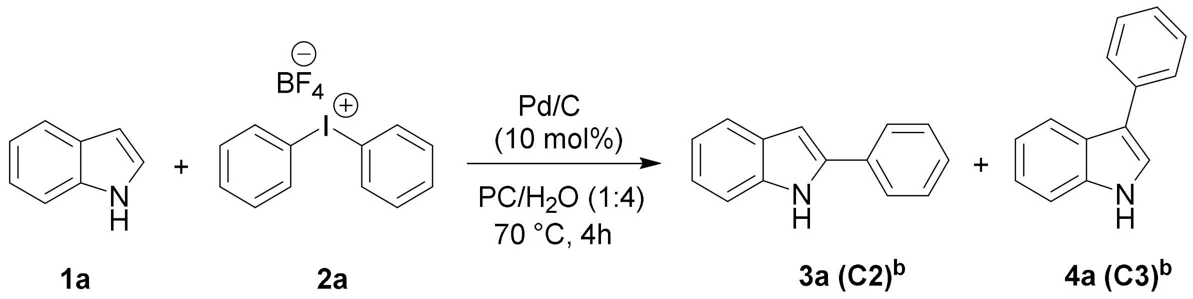

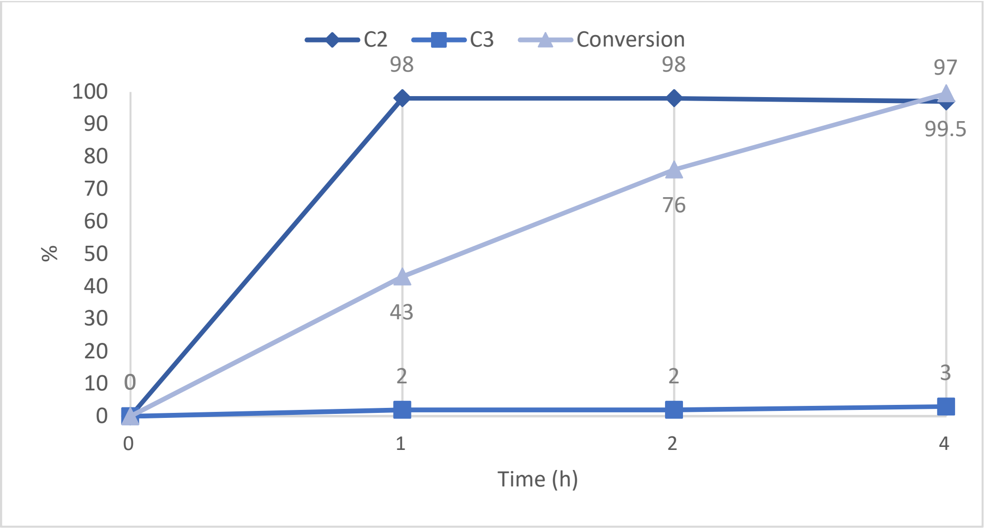

[a] Reaction conditions: **1a** (0.2 mmol), **2a** (1.25 eq), PC/H_2O 1:4 (1mL) at 70 °C for 4 h. [b] Determined by GLC and ^{1}H-NMR analyses.

Hot-filtration test

Two reactions between substrates indole **1a** and **2a** were run in parallel, in the optimized batch conditions. After 1 hours, one of the two reactions was removed from the heating and immediately subjected to cotton filtration and the reaction was allowed to stir at the reaction conditions for additional 3 hours. The two reactions were monitored by GLC analysis.

Table S3.

	**1h[b]**	**4h[b]**
Control reaction[a]	43%	99%
Filtered reaction[a]	46%	50%

[a] Reaction conditions: **1a** (0.2 mmol), **2a** (1.25 eq), PC/H_2O 1:4 (1mL) at 70 °C for 4 h. [b] Conversion determined by GLC analysis.

Hg-poisoning test

Two reactions between substrates **1a** and **2a** were run in parallel, in the optimized batch conditions. After 1 hours, in one of the two reactions 100 equivalents (relative to the catalyst) of Hg(0) were added and the reaction was allowed to stir at the reaction conditions for additional 3 hours. The two reactions were monitored by GLC analysis.

Table S4.

	**1h[b]**	**4h[b]**
Control reaction[a]	42%	99%
Hg-poisoned reaction[a]	45%	47%

[a] Reaction conditions: **1a** (0.2 mmol), **2a** (1.25 eq), PC/H_2O 1:4 (1mL) at 70 °C for 4 h. [b] Conversion determined by GLC analysis.

E-factor calculation for the typical procedure using Polarclean/water (4:1), Pd/C and 2-propanol for the isolation by re-crystallization (no column chromatography).

Reaction of Indole (1a) and diphenyliodonium tetrafluoroborate (2a)

E-factor = [585 mg (**1a**) + 2300 mg (**2a**) + 532.5 (Pd/C) + 20000 mg (H_2O) + 5200 mg (PC) + 19650 mg (2-propanol)] - [532.5 (Pd/C) + 20000 mg (H_2O) + 5200 mg (PC) + 17685 mg (2-propanol) + 803 mg (**product**)] / 803 mg (**product**) = 5.04

Figure S3. Radial Pentagon, for the protocol using 2-propanol.

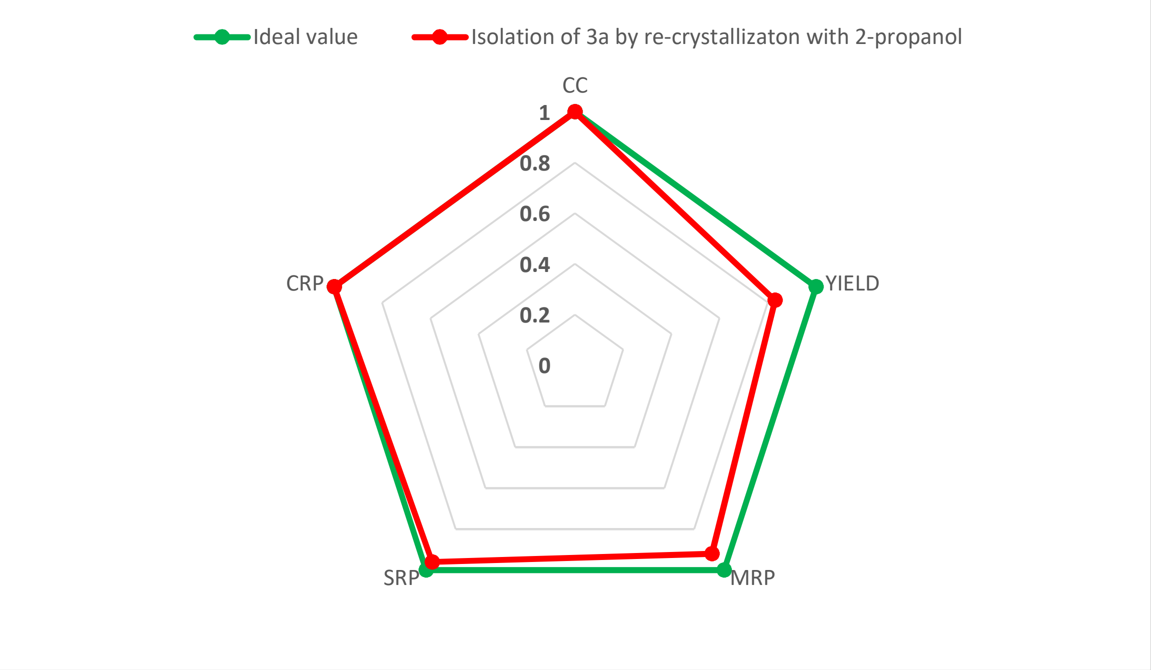

E-factor= Isolation of **3a** re-crystallization wiith 2-propanol (25mL) = 5.04

CC (column cromatography) = no = 1

Yield= 0.83

MRP = [803 (product) + 17685 (2-propanol recovered) + 5200 (PC recovered) + 20000 (H_20 recovered) + 532.5 (cat. recovered)] / [19650 (2-propanol) + 5200 (PC) + 20000 (H_20) + 532.5 (cat.) + 585 (indole) + 2300 (salt)] = 0.92

SRP: 44850 mg (total solvent used) : 100% = 42885 mg : X % = 0.96%

CRP (cat. recovery parameter) = complete reclaim = 1

E-factor calculation for the typical procedure using Polarclean/water (4:1), Pd/C and EtOAc work-up. 21.46 < E-factor values< 72.31

Reaction of Indole (1a) and diphenyliodonium tetrafluoroborate (2a)
E-factor = [23.4 mg (**1a**) + 92 mg (**2a**) + 21.3 (Pd/C) + 800 mg (H_2O) + 208 mg (PC) + 902 mg (EtOAc)] - [21.3 (Pd/C) + 800 mg (H_2O) + 208 mg (PC) + 36 mg (**product**)] / 36 mg (**product**) = 27.26

Reaction of 1-methyl-1*H*-indole (1b) and diphenyliodonium tetrafluoroborate (2a)
E-factor = [26.2 mg (**1b**) + 92 mg (**2a**) + 21.3 (Pd/C) + 800 mg (H_2O) + 208 mg (PC) + 902 mg (EtOAc)] - [21.3 (Pd/C) + 800 mg (H_2O) + 208 mg (PC) + 38 mg (**product**)] / 38 mg (**product**) = 25.84

Reaction of 5-methyl-1*H*-indole (1c) and diphenyliodonium tetrafluoroborate (2a)
E-factor = [26.2 mg (**1c**) + 92 mg (**2a**) + 21.3 (Pd/C) + 800 mg (H_2O) + 208 mg (PC) + 902 mg (EtOAc)] - [21.3 (Pd/C) + 800 mg (H_2O) + 208 mg (PC) + 28 mg (**product**)] / 28 mg (**product**) = 35.43

Reaction of 5-methoxy-1*H*-indole (1d) and diphenyliodonium tetrafluoroborate (2a)
E-factor = [28.8 mg (**1d**) + 92 mg (**2a**) + 21.3 (Pd/C) + 800 mg (H_2O) + 208 mg (PC) + 902 mg (EtOAc)] - [21.3 (Pd/C) + 800 mg (H_2O) + 208 mg (PC) + 36 mg (**product**)] / 36 mg (**product**) = 27.41

Reaction of 1*H*-indol-5-ol (1e) and diphenyliodonium tetrafluoroborate (2a)
E-factor = [26.6 mg (**1e**) + 92 mg (**2a**) + 21.3 (Pd/C) + 800 mg (H_2O) + 208 mg (PC) + 902 mg (EtOAc)] - [21.3 (Pd/C) + 800 mg (H_2O) + 208 mg (PC) + 23 mg (**product**)] / 23 mg (**product**) = 43.37

Reaction of 5-bromo-1*H*-indole (1f) and diphenyliodonium tetrafluoroborate (2a)
E-factor = [39.2 mg (**1f**) + 92 mg (**2a**) + 21.3 (Pd/C) + 800 mg (H_2O) + 208 mg (PC) + 902 mg (EtOAc)] - [21.3 (Pd/C) + 800 mg (H_2O) + 208 mg (PC) + 46 mg (**product**)] / 46 mg (**product**) = 21.46

Reaction of 5-cloro-1*H*-indole (1g) and diphenyliodonium tetrafluoroborate (2a)
E-factor = [30.3 mg (**1g**) + 92 mg (**2a**) + 21.3 (Pd/C) + 800 mg (H_2O) + 208 mg (PC) + 902 mg (EtOAc)] - [21.3 (Pd/C) + 800 mg (H_2O) + 208 mg (PC) + 37 mg (**product**)] / 37 mg (**product**) = 26.68

Reaction of 5-fluoro-1*H*-indole (1h) and diphenyliodonium tetrafluoroborate (2a)
E-factor = [26.8 mg (**1h**) + 92 mg (**2a**) + 21.3 (Pd/C) + 800 mg (H_2O) + 208 mg (PC) + 902 mg (EtOAc)] - [21.3 (Pd/C) + 800 mg (H_2O) + 208 mg (PC) + 35 mg (**product**)] / 35 mg (**product**) = 28.16

Reaction of 5-fluoro-1-methyl-1*H*-indole (1i) and diphenyliodonium tetrafluoroborate (2a)
E-factor = [29.8 mg (**1i**) + 92 mg (**2a**) + 21.3 (Pd/C) + 800 mg (H_2O) + 208 mg (PC) + 902 mg (EtOAc)] - [21.3 (Pd/C) + 800 mg (H_2O) + 208 mg (PC) + 22 mg (**product**)] / 22 mg (**product**) = 45.53

Reaction of indole-5-carbonitrile (1j) and diphenyliodonium tetrafluoroborate (2a)
E-factor = [28.4 mg (**1j**) + 92 mg (**2a**) + 21.3 (Pd/C) + 800 mg (H_2O) + 208 mg (PC) + 902 mg (EtOAc)] - [21.3 (Pd/C) + 800 mg (H_2O) + 208 mg (PC) + 17 mg (**product**)] / 17 mg (**product**) = 59.14

Reaction of 5-nitro-1*H*-indole (1k) and diphenyliodonium tetrafluoroborate (2a)
E-factor = [32.4 mg (**1k**) + 92 mg (**2a**) + 21.3 (Pd/C) + 800 mg (H_2O) + 208 mg (PC) + 902 mg (EtOAc)] - [21.3 (Pd/C) + 800 mg (H_2O) + 208 mg (PC) + 14 mg (**product**)] / 14 mg (**product**) = 72.31

Reaction of indole (1a) and di-*p*-tolyliodonium tetrafluoroborate (2b)
E-factor = [23.4 mg (**1a**) + 99 mg (**2b**) + 21.3 (Pd/C) + 800 mg (H_2O) + 208 mg (PC) + 902 mg (EtOAc)] - [21.3 (Pd/C) + 800 mg (H_2O) + 208 mg (PC) + 32 mg (**product**)] / 32 mg (**product**) = 31.01

Reaction of indole (1a) and bis(4-bromophenyl)iodonium tetrafluoroborate (2c)
E-factor = [23.4 mg (**1a**) + 131.4 mg (**2c**) + 21.3 (Pd/C) + 800 mg (H_2O) + 208 mg (PC) + 902 mg (EtOAc)] - [21.3 (Pd/C) + 800 mg (H_2O) + 208 mg (PC) + 45 mg (**product**)] / 45 mg (**product**) = 22.48

Reaction of indole (1a) and bis(4-chlorophenyl) tetrafluoroborate (2d)
E-factor = [23.4 mg (**1a**) + 109 mg (**2d**) + 21.3 (Pd/C) + 800 mg (H_2O) + 208 mg (PC) + 902 mg (EtOAc)] - [21.3 (Pd/C) + 800 mg (H_2O) + 208 mg (PC) + 36 mg (**product**)] / 36 mg (**product**) = 27.73

Reaction of indole (1a) and bis(4-cyanophenyl)iodonium tetrafluoroborate (2e)
E-factor = [23.4 mg (**1a**) + 104.5 mg (**2e**) + 21.3 (Pd/C) + 800 mg (H_2O) + 208 mg (PC) + 902 mg (EtOAc)] - [21.3 (Pd/C) + 800 mg (H_2O) + 208 mg (PC) + 37 mg (**product**)] / 37 mg (**product**) = 26.83

Reaction of indole (1a) and di(naphtalen-2-yl)iodonium tetrafluoroborate (2f)
E-factor = [23.4 mg (**1a**) + 117 mg (**2f**) + 21.3 (Pd/C) + 800 mg (H_2O) + 208 mg (PC) + 902 mg (EtOAc)] - [21.3 (Pd/C) + 800 mg (H_2O) + 208 mg (PC) + 39 mg (**product**)] / 39 mg (**product**) = 25.67

Reaction of indole (1a) and bis(4-methoxyphenyl)iodonium tetrafluoroborate (2g)

E-factor = [23.4 mg (**1a**) + 107 mg (**2g**) + 21.3 (Pd/C) + 800 mg (H_2O) + 208 mg (PC) + 902 mg (EtOAc)] - [21.3 (Pd/C) + 800 mg (H_2O) + 208 mg (PC) + 32 mg (**product**)] / 32 mg (**product**) = 31.26

Reaction of indole (1a) and bis(4-methoxyphenyl)iodonium tosylate (2h)

E-factor = [23.4 mg (**1a**) + 128 mg (**2h**) + 21.3 (Pd/C) + 800 mg (H_2O) + 208 mg (PC) + 902 mg (EtOAc)] - [21.3 (Pd/C) + 800 mg (H_2O) + 208 mg (PC) + 35 mg (**product**)] / 35 mg (**product**) = 29.09

Reaction of indole (1a) and di(thien-2-yl)iodonium tosylate (2i)

E-factor = [23.4 mg (**1a**) + 116 mg (**2i**) + 21.3 (Pd/C) + 800 mg (H_2O) + 208 mg (PC) + 902 mg (EtOAc)] - [21.3 (Pd/C) + 800 mg (H_2O) + 208 mg (PC) + 18 mg (**product**)] / 18 mg (**product**) = 56.85

Figure S4. Radial Pentagon, for the protocol using EtOAc.

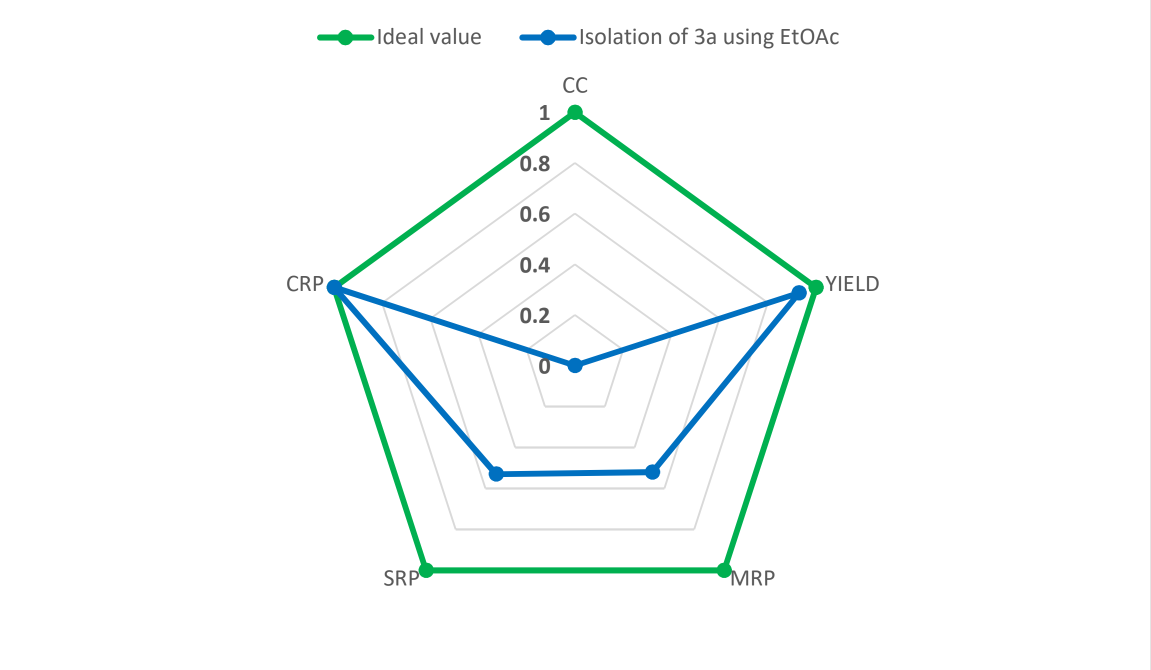

E-factor= Isolation of **3a** using EtOAc (1 mL) = 27.26

CC = yes = 0

Yield= 0.93

MRP = [36 (product) + 208 (PC recovered) + 800 (H_20 recovered) + 21.3 (cat. recovered)] / [902(EtOAc) + 208 (PC) + 800 (H_20) + 21.3 (cat.) + 23.4 (indole) + 92 (salt)] = 0.52

SRP: 1910:100 %= 1008 mg: x % = 0.53

CRP (cat. recovery parameter) = complete reclaim = 1

Table S5. E-Factor calculations and comparison with literature examples

Chem. Eur. J. **2014**, *20*, 13535-13531	E-factor = [58.5 mg (indole) + 368 (diphenyliodonium tetrafluoroborate) + 2000 mg (H_20) + 16.12 mg (catalyst) 4704 mg (acetone for washing) + 26600 mg (CH_2Cl_2)] – [16.12 mg (catalyst) + 87.82 mg (product) / 87.82 (product) = 383.08
J. Org. Chem. **2019***,* *84*, 10764−10774	E-factor = {117 mg (indole) +224.41 mg (iodobenzene) + 11.22 mg [$Pd(OAc)_2$] +183.6 (AgOAc)+ 30025 (AcOH) + 339 (PEG 400) + 9020 (EtOAc) + 10000 (H_2O)} – 189 mg (product) / 189 mg (product) = 104.42
Green Chem. **2019**, *21*, 1448-1454	E-factor = {23.4 mg (indole) + 47.10 mg (bromobenzene) + 1.04 mg [$(cinnamyl)PdCl]_2$ + 1.64 mg (DPPP) + 41.4 mg (K_2CO_3) + 980 mg (H_2O) + 20 mg (SPGS-500-M) + 1804 mg (EtOAc)} – [980 mg (H_2O) + 20 mg (SPGS-500-M) + 28.6 (product)] / 28.6 (product) = 66.08

Note: Isolation of the pure arylated indole products, required in all the reported cases a purification by column-chromatography. In all cases except for our protocol using 2-propanol which allow the isolation of pure product by re-crystallization.
Moreover, the re-crystallization procedure can be applied to all the C2-arylated indoles.
Accordingly, for the aim of this comparison, the E-factor values have been calculated without considering the waste associated to the column chromatography purification. it should be kept in mind anyway, that if chromatography is needed waste produced is hugely larger.

Chem. Name	2-Phenyl-1*H*-indole (3a)
Lit. Ref.	*Green Chem.* **2019**, *21*, 1448-1454

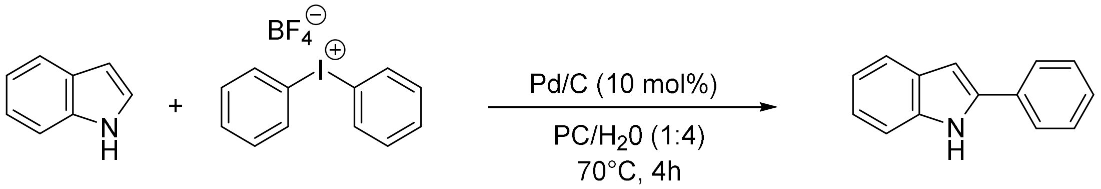

1a **2a** **3a**

Mol. Wt.: 193.249

METHOD:
In a screw capped vial equipped with a magnetic stirrer, indole (**1a**) (0.2 mmol, 23.4 mg), Pd/C (10 mol%, 21.3 mg), 1 mL of PC/H_2O (1:4) and diphenyliodonium tetrafluoroborate (**2a**) (0.25 mmol, 92 mg) were consecutively added and the resulting mixture was left under stirring at 70°C. After 4 h the reaction mixture was left to cool to room temperature, then centrifuged to separate and recover the medium, PC/H_2O. The solid residue was then washed with hot ethyl acetate (2x0.5 mL), which was recovered as supernatant after centrifugation (6500 rpm, 15 min). The combined organic layers were concentred under reduced pressure and the crude oil purified by flash chromatography on silica gel (PE/EtOAc 9:1) to afford the title compound (**3a**) as a white solid, 36 mg (93%).

Mol Formula	$C_{14}H_{11}N$	m.p.	190.5 °C

	δ value	No. H	Mult.	j value/Hz
**^{1}H NMR**	6.85	1	*dd*	2.2; 0.9
**400 MHz**	7.15	1	*ddd*	8.0, 7.1; 1.0
**$CDCl_3$**	7.22	1	*ddd*	8.1; 7.1; 1.3
	7.35	1	*ddt*	8.0; 6.7; 1.2
	7.41	1	*d*	8.1
	7.48-7.44	2	*m*	
	7.69-7.65	3	*m*	
	8.32	1	*bs*	

^{13}C NMR (100.6 MHz, $CDCl_3$) δ : 100.1, 111.0, 120.4, 120.8, 122.5, 125.3, 127.8, 129.2, 129.4, 132.5, 136.9, 138.0

GC-EIMS (m/z, %): 165 (20), 193 (100)

Chem. Name	1-Methyl-2-phenyl-1*H*-indole (3b)
Lit. Ref.	*New J. Chem.* **2019**, *43*, 9255-9259

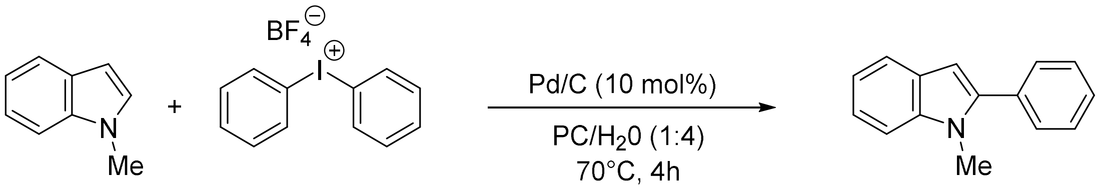

1b 2a 3b

Mol. Wt.: 207.276

METHOD:
In a screw capped vial equipped with a magnetic stirrer, 1-methyl-1*H*-indole (**1b**) (0.2 mmol, 26.2 mg), Pd/C (10 mol%, 21.3 mg), 1 mL of PC/H_2O (1:4) and diphenyliodonium tetrafluoroborate (**2a**) (0.25 mmol, 92 mg) were consecutively added and the resulting mixture was left under stirring at 70°C. After 4 h the reaction mixture was left to cool to room temperature, then centrifuged to separate and recover the medium, PC/H_2O. The solid residue was then washed with hot ethyl acetate (2x0.5 mL), which was recovered as supernatant after centrifugation (6500 rpm, 15 min). The combined organic layers were concentred under reduced pressure and the crude oil was purified by flash chromatography on silica gel (PE/EtOAc 9:1) to afford the title compound (**3b**) as a white solid, 38 mg (92%).

**Mol Formula**	$C_{15}H_{13}N$	**m.p.**	100-101 °C

	**δ value**	**No. H**	**Mult.**	**j value/Hz**
**^{1}H NMR**	3.79	3	*s*	
**400 MHz**	6.63	1	*d*	0.8
**$CDCl_3$**	7.21	1	*ddd*	8.0; 7.1; 1.0
	7.31	1	*ddd*	8.2; 7.1; 1.2
	7.42	1	*d*	8.2
	7.45	1	*ddt*	8.2; 6.2, 1.5
	7.54-7.49	2	*m*	
	7.58-7.55	2	*m*	
	7.71-7.69	1	*dt*	7.8; 1.0

^{13}C NMR (100.6 MHz, $CDCl_3$) δ : 31.3, 101.8, 109.7, 120.0, 120.6, 121.8, 128.0, 128.1, 128.6, 129.5, 133.0, 138.5, 141.7

GC-EIMS (m/z, %): 206 (98), 207 (100), 208 (39)

**Chem. Name**	**5-Methyl-2-phenyl-1*H*-indole (3c)**
**Lit. Ref.**	*Green Chem.* **2019**, *21*, 1448-1454

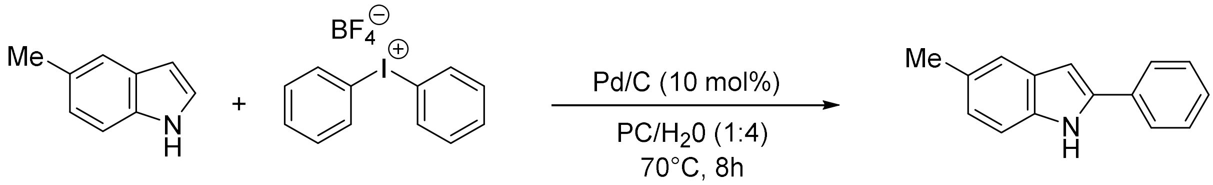

1c 2a 3c

Mol. Wt.: 207.276

METHOD:
In a screw capped vial equipped with a magnetic stirrer, 5-methyl-1*H*-indole (**1c**) (0.2 mmol, 26.2 mg), Pd/C (10 mol%, 21.3 mg), 1 mL of PC/H_2O (1:4) and diphenyliodonium tetrafluoroborate (**2a**) (0.25 mmol, 92 mg) were consecutively added and the resulting mixture was left under stirring at 70°C. After 8 h the reaction mixture was left to cool to room temperature, then centrifuged to separate and recover the medium, PC/H_2O. The solid residue was then washed with hot ethyl acetate (2x0.5 mL), which was recovered as supernatant after centrifugation (6500 rpm, 15 min). The combined organic layers were concentred under reduced pressure and the crude oil was purified by flash chromatography on silica gel (PE/EtOAc 9:1) to afford the title compound (**3c**) as a white solid, 28 mg (68%).

**Mol Formula**	$C_{15}H_{13}N$	**m.p.**	214-215 °C

	**δ value**	**No. H**	**Mult.**	**j value/Hz**
**^{1}H NMR**	2.48	3	*s*	
**400 MHz**	6.77	1	*dd*	2.1; 0.7
**$CDCl_3$**	7.04	1	*dd*	8.3; 1.5
	7.30	1	*d*	8.4
	7.33	1	*ddt*	8.0; 6.9; 1.2
	7.47-7.43	3	*m*	
	7.67-7.64	2	*m*	
	8.22	1	*bs*	

^{13}C NMR (100.6 MHz, $CDCl_3$) δ: 21.6, 99.7, 110.7, 120.4, 124.1, 125.2, 127.7. 129.1, 129.6, 129.7, 132.6, 135.3, 138.1

GC-EIMS (m/z, %): 206 (65), 207 (100)

**Chem. Name**	**5-Methoxy-2-phenyl-1*H*-indole (3d)**
**Lit. Ref.**	*Angew. Chem. Int. Ed.* **2019**, *58*, 11033–11038

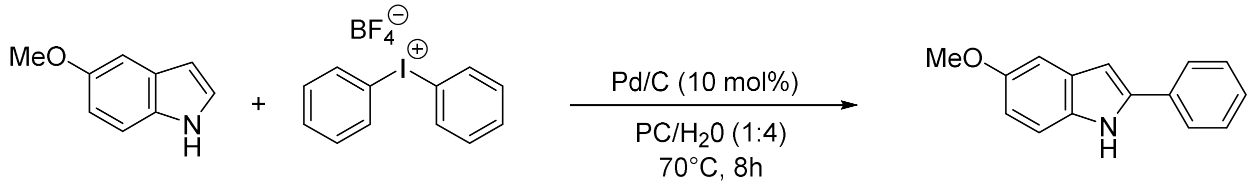

1d **2a** **3d**

Mol. Wt.: 223.275

METHOD:
In a screw capped vial equipped with a magnetic stirrer, 5-methoxy-1*H*-indole (**1d**) (0.2 mmol, 28.8 mg), Pd/C (10 mol%, 21.3 mg), 1 mL of PC/H_2O (1:4) and diphenyliodonium tetrafluoroborate (**2a**) (0.25 mmol, 92 mg) were consecutively added and the resulting mixture was left under stirring at 70°C. After 8h the reaction mixture was left to cool to room temperature, then centrifuged to separate and recover the medium, PC/H_2O. The solid residue was then washed with hot ethyl acetate (2x0.5 mL), which was recovered as supernatant after centrifugation (6500 rpm, 15 min). The combined organic layers were concentred under reduced pressure and the crude oil was purified by flash chromatography on silica gel (PE/EtOAc 9:1) to afford the title compound (**3d**) as a white solid, 36 mg (81%).

**Mol Formula**	$C_{15}H_{13}NO$	**m.p.**	172-174 °C

	**δ value**	**No. H**	**Mult.**	**j value/Hz**	
**^{1}H NMR**	3.88	3	*s*		
**400 MHz**	6.77	1	*dd*	2.2; 0.9	
**$CDCl_3$**	6.87	1	*dd*	8.8; 2.5	
	7.10	1	*d*	2.5	
	7.29	1	*d*	8.8	
	7.32	1	*ddt*	8.0; 6.8; 1.2	
	7.46-7.42	2	*m*		
	7.66-7.63	2	*m*		
	8.24	1	*bs*		

^{13}C NMR (100.6 MHz, $CDCl_3$) δ : 56.0, 100.0, 102.4, 111.8, 112.8, 125.2, 127.8, 129.1, 129.9, 132.1, 132.6, 138.7, 154.6

GC-EIMS (m/z, %): 180 (67), 208 (38), 223 (100)

**Chem. Name**	**2-Phenyl-1*H*-indol-5-ol (3e)**
**Lit. Ref.**	*Nat.Commun*. **2019**, *10*, 908

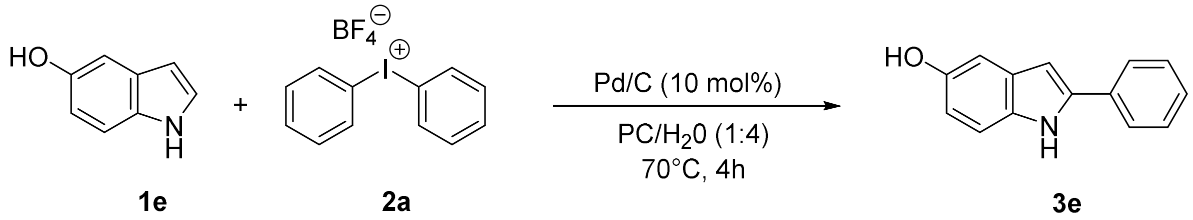

Mol. Wt.: 209.248

METHOD:
In a screw capped vial equipped with a magnetic stirrer, 1*H*-indol-5-ol (**1e**) (0.2 mmol, 26.6 mg), Pd/C (10 mol%, 21.3 mg), 1 mL of PC/H_2O (1:4) and diphenyliodonium tetrafluoroborate (**2a**) (0.25 mmol, 92 mg) were consecutively added and the resulting mixture was left under stirring at 70°C. After 4h the reaction mixture was left to cool to room temperature, then centrifuged to separate and recover the medium, PC/H_2O. The solid residue was then washed with hot ethyl acetate (2x0.5 mL), which was recovered as supernatant after centrifugation (6500 rpm, 15 min). The combined organic layers were concentred under reduced pressure and the crude oil was purified by flash chromatography on silica gel (PE/EtOAc 6:4) to afford the title compound (**3e**) as a white solid, 23 mg (55%).

**Mol Formula**	C_8H_7NO	**m.p.**	246-251 °C

	**δ value**	**No. H**	**Mult.**	**j value/Hz**
**^{1}H NMR 400 MHz DMSO-*d*$_6$**	6.61	1	*dd*	8.6, 1.9
	6.71	1	*s*	
	6.83	1	*d*	1.4
	7.18	1	*d*	8.6
	7.28	1	*t*	7.3
	7.43	2	*t*	7.7
	7.80	2	*d*	7.6
	8.66	1	*s*	
	11.19	1	*s*	

^{13}C NMR (100.6 MHz, DMSO-*d*$_6$) δ : 98.8, 104.7, 112.6, 112.7, 125.5, 128.1, 129.8, 130.2, 132.6, 133.0, 138.8, 151.3

GC-EIMS (m/z, %): 77 (21), 105 (25), 152 (33), 180 (70), 209 (100), 210 (63)

**Chem. Name**	**5-Bromo-2-phenyl-1*H*-indole (3f)**
**Lit. Ref.**	*Chem. Eur. J.* **2014**, *20*, 13531-13535

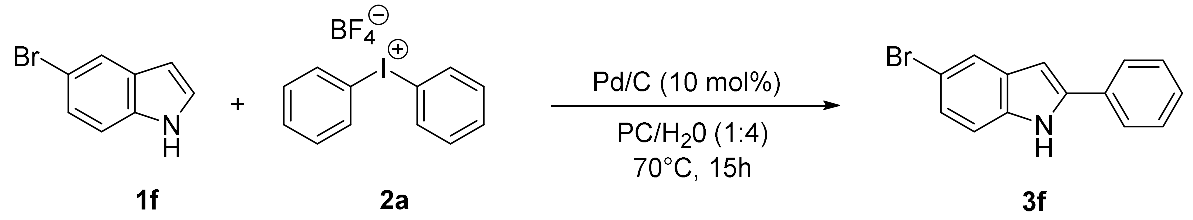

Mol. Wt.: 272.145

METHOD:
In a screw capped vial equipped with a magnetic stirrer, 5-bromo-1*H*-indole (**1f**) (0.2 mmol, 39.2 mg), Pd/C (10 mol%, 21.3 mg), 1 mL of PC/H_2O (1:4) and diphenyliodonium tetrafluoroborate (**2a**) (0.25 mmol, 92 mg) were consecutively added and the resulting mixture was left under stirring at 70°C. After 15h the reaction mixture was left to cool to room temperature, then centrifuged to separate and recover the medium, PC/H_2O. The solid residue was then washed with hot ethyl acetate (2x0.5 mL), which was recovered as supernatant after centrifugation (6500 rpm, 15 min). The combined organic layers were concentred under reduced pressure and the crude oil was purified by flash chromatography on silica gel (PE/EtOAc 9:1) to afford the title compound (**3f**) as a white solid, 46 mg (85%).

**Mol Formula**	$C_{14}H_{10}BrN$	**m.p.**	193-196°C

	**δ value**	**No. H**	**Mult.**	**j value/Hz**
**^{1}H NMR**	6.75	1	*d*	1.5
**400 MHz**	7.27	2	*s*	
**$CDCl_3$**	7.35	1	*t*	7.2
	7.46	2	*t*	7.6
	7.65	2	*d*	7.7
	7.75	1	*s*	
	8.37	1	*bs*	

^{13}C NMR (100.6 MHz, $CDCl_3$) δ : 99.6, 112.4, 113.6, 123.2, 125.3, 125.4, 128.3, 129.3, 131.1, 131.9, 135.5, 139.3

GC-EIMS (m/z, %): 165 (32), 191 (27), 271 (100), 273 (96)

**Chem. Name**	**5-Chloro-2-phenyl-1*H*-indole (3g)**
**Lit. Ref.**	*Angew. Chem. Int. Ed.* **2019**, *58*,11033–11038

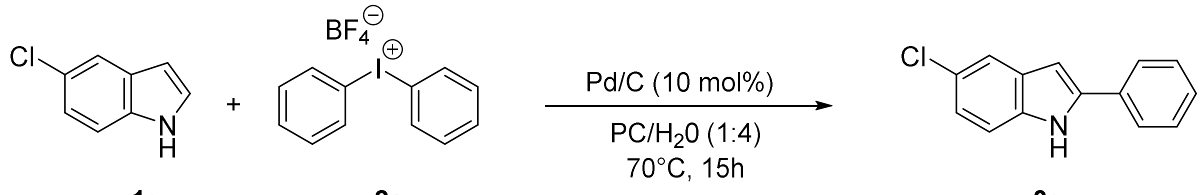

Mol. Wt.: 227.691

METHOD:
In a screw capped vial equipped with a magnetic stirrer, 5-cloro-1*H*-indole (**1g**) (0.2 mmol, 30.3 mg), Pd/C (10 mol%, 21.3 mg), 1 mL of PC/H_2O (1:4) and diphenyliodonium tetrafluoroborate (**2a**) (0.25 mmol, 92 mg) were consecutively added and the resulting mixture was left under stirring at 70°. After 15h the reaction mixture was left to cool to room temperature, then centrifuged to separate and recover the medium, PC/H_2O. The solid residue was then washed with hot ethyl acetate (2x0.5 mL), which was recovered as supernatant after centrifugation (6500 rpm, 15 min). The combined organic layers were concentred under reduced pressure and the crude oil was purified by flash chromatography on silica gel (PE/EtOAc 9:1) to afford the title compound (**3g**) as a white solid, 37 mg (81%).

**Mol Formula**	$C_{14}H_{10}ClN$	**m.p.**	191°C

	**δ value**	**No. H**	**Mult.**	**j value/Hz**
**^{1}H NMR**	6.76	1	*s*	
**400 MHz**	7.14	1	*dd*	8.5; 1.7
**$CDCl_3$**	7.30	1	*d*	8.5
	7.35	1	*t*	7.5
	7.46	2	*t*	7.7
	7.59	1	*s*	
	7.66-7.64	2	*d*	7.8
	8.34	1	*bs*	

^{13}C NMR (100.6 MHz, $CDCl_3$) δ : 99.7, 112.0, 120.1, 122.7, 125.4, 126.0, 128.3, 129.2, 130.5, 132.0, 135.2, 139.4

GC-EIMS (m/z, %): 165 (21), 227 (100), 228 (23), 229 (38)

**Chem. Name**	**5-Fluoro-2-phenyl-1*H*-indole (3h)**
**Lit. Ref.**	*Angew. Chem. Int. Ed.* **2019**, *58,*11033–11038

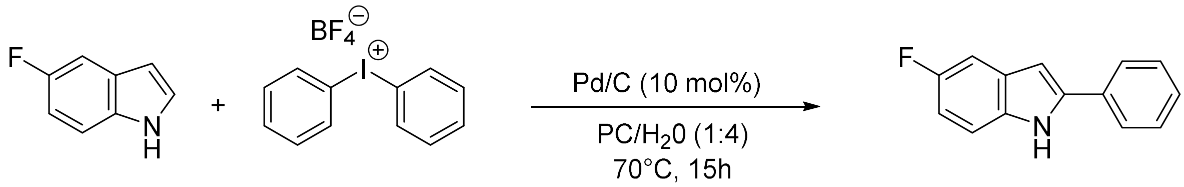

1h **2a** **3h**

Mol. Wt.: 211.239

METHOD:
In a screw capped vial equipped with a magnetic stirrer, 5-fluoro-1*H*-indole (**1h**) (0.2 mmol, 26.8 mg), Pd/C (10 mol%, 21.3 mg), 1 mL of PC/H_2O (1:4) and diphenyliodonium tetrafluoroborate (**2a**) (0.25 mmol, 92 mg) were consecutively added and the resulting mixture was left under stirring at 70°C. After 15h the reaction mixture was left to cool to room temperature, then centrifuged to separate and recover the medium, PC/H_2O. The solid residue was then washed with hot ethyl acetate (2x0.5 mL), which was recovered as supernatant after centrifugation (6500 rpm, 15 min). The combined organic layers were concentred under reduced pressure and the crude oil was purified by flash chromatography on silica gel (PE/EtOAc 9:1) to afford the title compound (**3h**) as a white solid, 35 mg (83%).

**Mol Formula**	$C_{14}H_{10}FN$	**m.p.**	178-179 °C

	**δ value**	**No. H**	**Mult.**	**j value/Hz**
**^{1}H NMR 400 MHz $CDCl_3$**	6.79	1	*s*	
	6.94	1	*td*	9.0; 1.9
	7.37-7.28	3	*m*	
	7.46	2	*t*	7.6
	7.65	2	*d*	7.8
	8.33	1	*bs*	

^{13}C NMR (100.6 MHz, $CDCl_3$) δ : 100.2 (d, J^4_{C-F} = 4.6),105.5 (d, J^2_{C-F} = 23.5), 110.8 (d, J^2_{C-F} = 26.4), 111.6 (d, J^3_{C-F} = 9.7), 125.3, 128.2, 129.2, 129.8 (d, J^3_{C-F} = 10.4), 132.2, 133.5, 139.8, 158.3 (d, J^1_{C-F} = 234.8)

GC-EIMS (m/z, %): 183 (22), 211 (100)

**Chem. Name**	**5-Fluoro-1-methyl-2-phenyl-1*H*-indole (3i)**
**Lit. Ref.**	*Organometallics* **2017**, *36*, 767-776

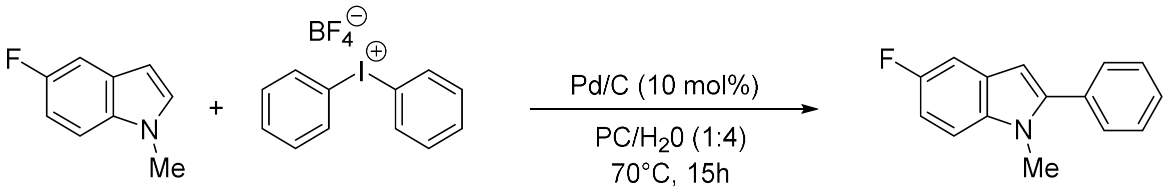

1i 2a 3i

Mol. Wt.: 225.266

METHOD:
In screw capped vial equipped with a magnetic stirrer, 5-fluoro-1-methyl-1*H*-indole (**1i**) (0.2 mmol, 29.8 mg), Pd/C (10 mol%, 21.3 mg), 1 mL of PC/H_2O (1:4) and diphenyliodonium tetrafluoroborate (**2a**) (0.25 mmol, 92 mg) were consecutively added and the resulting mixture was left under stirring at 70°C. After 15h the reaction mixture was left to cool to room temperature, then centrifuged to separate and recover the medium, PC/H_2O. The solid residue was then washed with hot ethyl acetate (2x0.5 mL), which was recovered as supernatant after centrifugation (6500 rpm, 15 min). The combined organic layers were concentred under reduced pressure and the crude oil was purified by flash chromatography on silica gel (PE/EtOAc 9:1) to afford the title compound (**3i**) as a white solid, 22 mg (50%).

**Mol Formula**	$C_{15}H_{12}FN$	**m.p.**	107 °C

	**δ value**	**No. H**	**Mult.**	**j value/Hz**
**^{1}H NMR 400 MHz $CDCl_3$**	3.75	3	*s*	
	6.53	1	*d*	0.7
	7.00	1	*td*	8.9; 2.5
	7.31-7.26	2	*m*	
	7.45-7.41	1	*m*	
	7.53-7.46	4	*m*	

^{13}C NMR (100.6 MHz, $CDCl_3$) δ : 31.5, 101.7 (d, J^4_{C-F} = 4.6), 105.3 (d, J^2_{C-F} = 23.4), 110.0 (d, J^2_{C-F} = 26.3), 110.3 (d, J^3_{C-F} = 9.8), 128.2 (d, J^3_{C-F} = 10.3), 128.2, 128.7, 129.5, 132.7, 135.1, 143.3, 158.2 (d, J^1_{C-F} = 234.3),

GC-EIMS (m/z, %): 224 (69), 225 (100), 226 (22)

**Chem. Name**	**2-Phenyl-1*H*-indole-5-carbonitrile (3j)**
**Lit. Ref.**	*Catal. Sci. Technol*. **2014**, *4*, 1979–1988

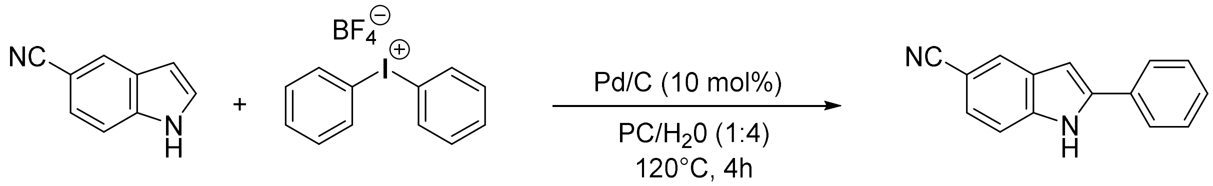

1j 2a 3j

Mol. Wt.: 218.259

METHOD:
In a screw capped vial equipped with a magnetic stirrer, indole-5-carbonitrile (**1j**) (0.2 mmol, 28.4 mg), Pd/C (10 mol%, 21.3 mg), 1 mL of PC/H_2O (1:4) and diphenyliodonium tetrafluoroborate (**2a**) (0.25 mmol, 92 mg) were consecutively added and the resulting mixture was left under stirring at 120°. After 4h the reaction mixture was left to cool to room temperature, then centrifuged to separate and recover the medium, PC/H_2O. The solid residue was then washed with hot ethyl acetate (2x0.5 mL), which was recovered as supernatant after centrifugation (6500 rpm, 15 min). The combined organic layers were concentred under reduced pressure and the crude oil was purified by flash chromatography on silica gel (PE/EtOAc 6:4) to afford the title compound (**3j**) as a white solid, 17 mg (39%).

**Mol Formula**	$C_{15}H_{10}N_2$	**m.p.**	193-195 °C

	**δ value**	**No. H**	**Mult.**	**j value/Hz**
**^{1}H NMR 400 MHz $CDCl_3$**	6.88	1	*dd*	1.6
	7.50-7.37	5	*m*	
	7.69-7.67	2	*m*	
	7.97	1	*s*	
	8.70	1	*bs*	

^{13}C NMR (100.6 MHz, $CDCl_3$) δ : 100.4, 103.5, 111.9, 120.8, 125.4, 125.6, 126.2, 128.8, 129.1, 129.4, 131.3, 138.5, 140.4

GC-EIMS (m/z, %): 190 (22), 218 (100)

**Chem. Name**	**5-Nitro-2-phenyl-1*H*-indole (3k)**
**Lit. Ref.**	*Chem. Eur. J.* **2014**, *20,* 13531 – 13535

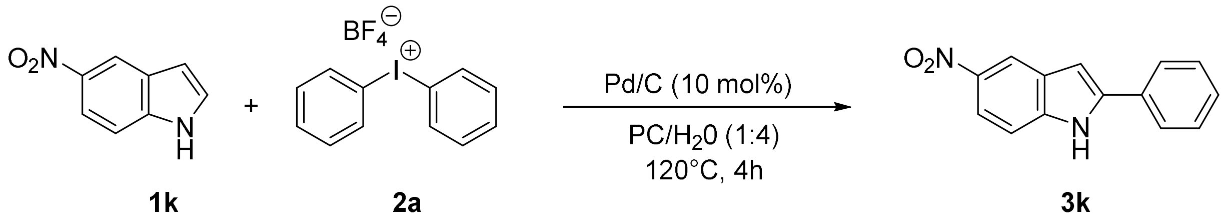

Mol. Wt.: 225.266

METHOD:
In a screw capped vial equipped with a magnetic stirrer, 5-nitro-1*H*-indole (**1k**) (0.2 mmol, 32.4 mg), Pd/C (10 mol%, 21.3 mg), 1 mL of PC/H_2O (1:4) and diphenyliodonium tetrafluoroborate (**2a**) (0.25 mmol, 92 mg) were consecutively added and the resulting mixture was left under stirring at 120°C. After 4h the reaction mixture was left to cool to room temperature, then centrifuged to separate and recover the medium, PC/H_2O. The solid residue was then washed with hot ethyl acetate (2x0.5 mL), which was recovered as supernatant after centrifugation (6500 rpm, 15 min). The combined organic layers were concentred under reduced pressure and the crude oil was purified by flash chromatography on silica gel (PE/EtOAc 8:2) to afford the title compound (**3k**) as a yellow solid, 14 mg (30%).

**Mol Formula**	$C_{14}H_{10}N_2O_2$	**m.p.**	234-235°C

	**δ value**	**No. H**	**Mult.**	**j value/Hz**	
**^{1}H NMR**	7.18	1	*s*		
**400 MHz**	7.40	1	*t*	7.3	
**Acetone-*d₆***	7.51	2	*t*	7.6	
	7.58	1	*d*	8.9	
	7.91	2	*d*	7.5	
	8.04	1	*dd*	8.9,1.9	
	8.57	1	*d*	1.4	
	11.37	1	*bs*		

^{13}C NMR (100.6 MHz, Acetone-d_6) δ : 101.9, 112.3, 117.9, 118.0, 126.4, 129.36, 129.44, 130.0, 132.3, 141.3, 142.6, 142.8

GC-EIMS (m/z, %): 191 (34), 192 (48), 238 (100)

**Chem. Name**	**2-(p-Tolyl)-1*H*-indole (3l)**
**Lit. Ref.**	*Angew. Chem. Int. Ed.* **2019**, *58*,11033–11038

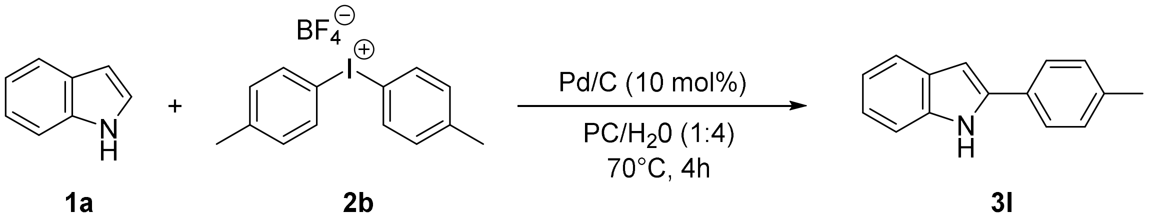

Mol. Wt.: 207.276

METHOD:
In a screw capped vial equipped with a magnetic stirrer, indole (**1a**) (0.2 mmol, 23.4 mg), Pd/C (10 mol%, 21.3 mg), 1 mL of PC/H_2O (1:4) and di-*p*-tolyliodonium tetrafluoroborate (**2b**) (0.25 mmol, 99 mg) were consecutively added and the resulting mixture was left under stirring at 70°C. After 4h the reaction mixture was left to cool to room temperature, then centrifuged to separate and recover the medium, PC/H_2O. The solid residue was then washed with hot ethyl acetate (2x0.5 mL), which was recovered as supernatant after centrifugation (6500 rpm, 15 min). The combined organic layers were concentred under reduced pressure and the crude oil was purified by flash chromatography on silica gel (PE/EtOAc 9:1) to afford the title compound (**3l**) as a white solid, 32 mg (77%).

**Mol Formula**	$C_{15}H_{13}N$	**m.p.**	214-215 °C

	**δ value**	**No. H**	**Mult.**	**j value/Hz**
**^{1}H NMR**	2.42	3	*s*	
**400 MHz**	6.81	1	*dd*	2.1; 0.8
**$CDCl_3$**	7.15	1	*ddd*	8.1; 7.2; 1.1
	7.21	1	*ddd*	8.2; 7.1; 1.2
	7.27	2	*d*	7.9
	7.40	1	*d*	7.9
	7.58-7.56	2	*m*	
	7.66-7.64	1	*d*	7.8
	8.30	1	*bs*	

^{13}C NMR (100.6 MHz, $CDCl_3$) δ: 21.4, 99.5, 110.9, 120.3, 120.6, 122.2, 125.2, 129.5, 129.7, 129.8, 136.8, 137.8, 138.2

GC-EIMS (m/z, %): 206 (31), 207 (100)

Chem. Name	2-(4-Bromophenyl)-1H-indole (3m)
Lit. Ref.	*Chem. Eur. J.* **2014**, *20*, 13531 – 13535

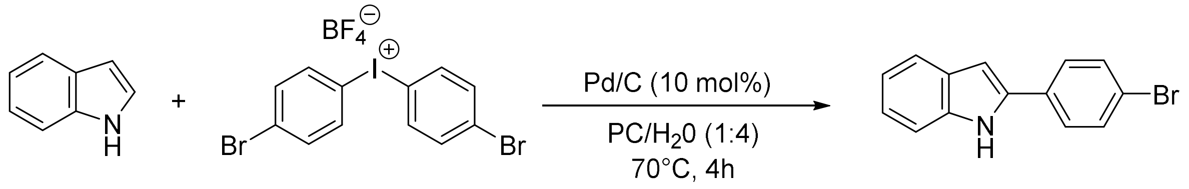

1a **2c** **3m**

Mol. Wt.: 272.145

METHOD:
In a screw capped vial equipped with a magnetic stirrer, indole (**1a**) (0.2 mmol, 23.4 mg), Pd/C (10 mol%, 21.3 mg), 1 mL of PC/H_2O (1:4) and bis(4-bromophenyl)iodonium tetrafluoroborate (**2c**) (0.25 mmol, 131.4 mg) were consecutively added and the resulting mixture was left under stirring at 70°C. After 4h the reaction mixture was left to cool to room temperature, then centrifuged to separate and recover the medium, PC/H_2O. The solid residue was then washed with hot ethyl acetate (2x0.5 mL), which was recovered as supernatant after centrifugation (6500 rpm, 15 min). The combined organic layers were concentred under reduced pressure and the crude oil was purified by flash chromatography on silica gel (PE/EtOAc 9:1) to afford the title compound (**3m**) as a white solid, 45 mg (83%).

Mol Formula	$C_{14}H_{10}BrN$	m.p.	211°C

	δ value	No. H	Mult.	j value/Hz
**^{1}H NMR**	6.83	1	*dd*	2.1; 0.8
**400 MHz**	7.14	1	*ddd*	8.0; 7.1; 1.0
**$CDCl_3$**	7.22	1	*ddd*	8.2; 7.1; 1.2
	7.40	1	*d*	8.1
	7.58-7.51	4	*m*	
	7.64	1	*d*	7.8
	8.27	1	*bs*	

^{13}C NMR (100.6 MHz, $CDCl_3$) δ: 100.7, 111.1, 120.6, 120.9, 121.7, 122.9, 126.7, 129.3, 131.5, 132.3, 136.8, 137.0

GC-EIMS (m/z, %): 165 (34), 191 (26), 271 (100), 273 (98)

Chem. Name	2-(4-Chlorophenyl)-1*H*-indole (3n)
Lit. Ref.	*Chem. Eur. J.* **2014**, *20*, 13531 – 13535

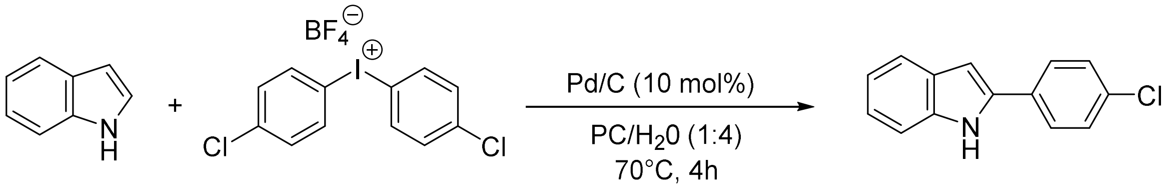

1a **2d** **3n**

Mol. Wt.: 227.691

METHOD:
In a screw capped vial equipped with a magnetic stirrer, indole (**1a**) (0.2 mmol, 23.4 mg), Pd/C (10 mol%, 21.3 mg), 1 mL of PC/H_2O (1:4) and bis(4-chlorophenyl) tetrafluoroborate (**2d**) (0.25 mmol, 109 mg) were consecutively added and the resulting mixture was left under stirring at 70°C. After 4h the reaction mixture was left to cool to room temperature, then centrifuged to separate and recover the medium, PC/H_2O. The solid residue was then washed with hot ethyl acetate (2x0.5 mL), which was recovered as supernatant after centrifugation (6500 rpm, 15 min). The combined organic layers were concentred under reduced pressure and the crude oil was purified by flash chromatography on silica gel (PE/EtOAc 9:1) to afford the title compound (**3n**) as a white solid, 36 mg (79%).

Mol Formula	$C_{14}H_{10}ClN$	m.p.	195-196°C

	δ value	No. H	Mult.	j value/Hz
**^{1}H NMR 400 MHz $CDCl_3$**	6.82	1	*dd*	2.2; 0.9
	7.14	1	*ddd*	8.1; 7.1; 1.1
	7.21	1	*ddd*	8.2; 7.1; 1.2
	7.43-7.39	3	*m*	
	7.60-7.57	2	*m*	
	7.63	1	*d*	7.9
	8.28	1	*bs*	

^{13}C NMR (100.6 MHz, $CDCl_3$) δ: 100.6, 111.1, 120.6, 120.9, 122.8, 126.5, 129.3, 129.4, 131.0, 133.6, 136.8, 137.0

GC-EIMS (m/z, %): 165 (25), 227 (100), 229 (31)

Chem. Name	4-(1*H*-Indol-2-yl)benzonitrile (3o)
Lit. Ref.	*Tetrahedron Letters* **2011**, *52,* 5625–5628

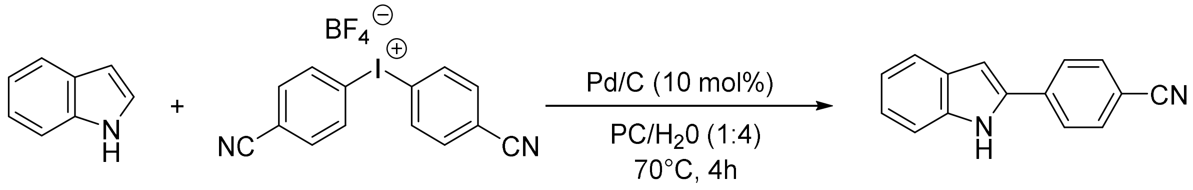

1a **2e** **3o**

Mol. Wt.: 233.294

METHOD:
In a screw capped vial equipped with a magnetic stirrer, indole (**1a**) (0.2 mmol, 23.4 mg), Pd/C (10 mol%, 21.3 mg), 1 mL of PC/H_2O (1:4) and bis(4-cyanophenyl)iodonium tetrafluoroborate (**2e**) (0.25 mmol, 104.5 mg) were consecutively added and the resulting mixture was left under stirring at 70°C. After 4h the reaction mixture was left to cool to room temperature, then centrifuged to separate and recover the medium, PC/H_2O. The solid residue was then washed with hot ethyl acetate (2x0.5 mL), which was recovered as supernatant after centrifugation (6500 rpm, 15 min). The combined organic layers were concentred under reduced pressure and the crude oil was purified by flash chromatography on silica gel (PE/EtOAc 9:1→6:4) to afford the title compound (**3o**) as a white solid, 37 mg (85%).

Mol Formula	$C_{15}H_{10}N_2$	m.p.	190-191°C

	δ value	No. H	Mult.	j value/Hz
**^{1}H NMR 400 MHz $CDCl_3$**	6.97-6.96	1	*s*	
	7.18-7.14	1	*t*	7.4
	7.28-7.24	1	*t*	7.1
	7.43	1	*d*	8.2
	7.66	1	*d*	7.9
	7.76-7.70	4	*m*	
	8.41	1	*bs*	

^{13}C NMR (100.6 MHz, $CDCl_3$) δ: 102.8, 110.7, 111.3, 119.0, 121.0, 121.4, 123.8, 125.4, 129.1, 133.0, 135.6, 136.7, 137.5

GC-EIMS (m/z, %): 190 (21), 218 (100)

Chem. Name	**2-(Naphthalen-1-yl)-1*H*-indole (3p)**
**Lit. Ref.**	*Org. Lett.* **2019**, *21,* 3053−3056

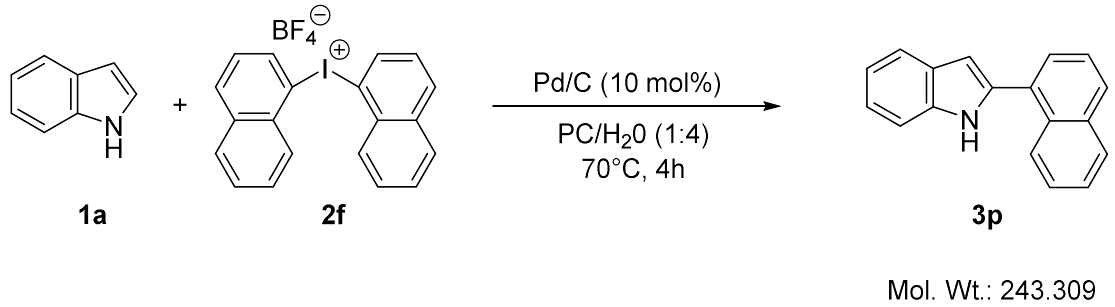

METHOD:
In a screw capped vial equipped with a magnetic stirrer, indole (**1a**) (0.2 mmol, 23.4 mg), Pd/C (10 mol%, 21.3 mg), 1 mL of PC/H_2O (1:4) and di(naphtalen-2-yl)iodonium tetrafluoroborate (**2f**) (0.25 mmol, 117 mg) were consecutively added and the resulting mixture was left under stirring at 70°C. After 4h the reaction mixture was left to cool to room temperature, then centrifuged to separate and recover the medium, PC/H_2O. The solid residue was then washed with hot ethyl acetate (2x0.5 mL), which was recovered as supernatant after centrifugation (6500 rpm, 15 min). The combined organic layers were concentred under reduced pressure and the crude oil was purified by flash chromatography on silica gel (PE/EtOAc 9:1) to afford the title compound (**3p**) as a white solid, 39 mg (80%).

**Mol Formula**	$C_{18}H_{13}N$	**m.p.**	97-99 °C

	**δ value**	**No. H**	**Mult.**	**j value/Hz**
**^{1}H NMR**	6.81	1	*d*	0.9
**400 MHz**	7.19	1	*t*	7.2
**$CDCl_3$**	7.25	1	*t*	7.2
	7.44	1	*d*	8.0
	7.56-7.50	3	*m*	
	7.64	1	*d*	7.0
	7.72	1	*d*	7.8
	7.94-7.88	2	*m*	
	8.30	1	*bs*	
	8.33-8.31	1	*m*	

^{13}C NMR (100.6 MHz, $CDCl_3$) δ: 103.9, 111.0, 120.3, 120.8, 122.4, 125.5, 125.8, 126.3, 126.8, 127.3, 128.6, 128.7, 129.0, 131.3, 131.7, 134.1, 136.5, 136.9

GC-EIMS (m/z, %): 241 (33), 242 (62), 243 (100)

Chem. Name	2-(4-Methoxyphenyl)-1*H*-indole (3q)
Lit. Ref.	*Catal. Sci. Techno*l. **2014**, *4*, 1979–1988

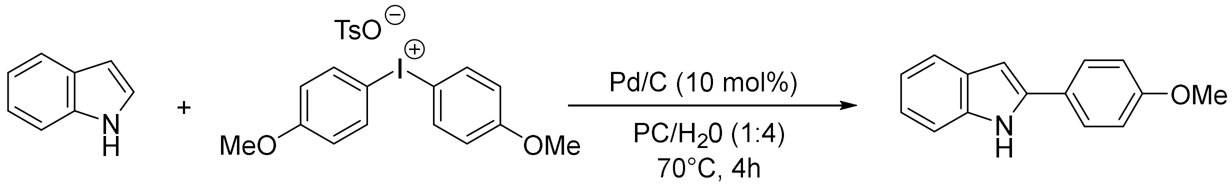

1a **2h** **3q**

Mol. Wt.: 223.275

METHOD:
In a screw capped vial equipped with a magnetic stirrer, indole (**1a**) (0.2 mmol, 23.4 mg), Pd/C (10 mol%, 21.3 mg), 1 mL of PC/H_2O (1:4) and bis(4-methoxyphenyl)iodonium tosylate (**2h**) (0.25 mmol, 128 mg) were consecutively added and the resulting mixture was left under stirring at 70°C. After 4h the reaction mixture was left to cool to room temperature, then centrifuged to separate and recover the medium, PC/H_2O. The solid residue was then washed with hot ethyl acetate (2x0.5 mL), which was recovered as supernatant after centrifugation (6500 rpm, 15 min). The combined organic layers were concentred under reduced pressure and the crude oil was purified by flash chromatography on silica gel (PE/EtOAc 9:1) to afford the title compound (**3q**) as a white solid, 35 mg (78%).

Mol Formula	$C_{15}H_{13}NO$	m.p.	228-229°C

	δ value	No. H	Mult.	j value/Hz
**^{1}H NMR**	3.84	3	*s*	
**400 MHz**	6.76	1	*d*	1.5
**Acetone-*d*$_6$**	7.03-6.98	3	*m*	
	7.07	1	*t*	7.9
	7.38	1	*d*	8.0
	7.52	1	*d*	7.8
	7.78	2	*d*	8.8
	10.53	1	*bs*	

^{13}C NMR (100.6 MHz, Acetone-*d*$_6$) δ: 55.6, 98.7, 111.8, 115.2, 120.3, 120.8, 122.1, 126.3, 127.3, 130.4, 138.2, 139.0, 160.3

GC-EIMS (m/z, %): 63 (25), 152 (27), 180 (29), 208 (65), 223 (100), 224 (21)

Chem. Name	2-(Thien-2-yl)-1*H*-indole (3r)
Lit. Ref.	*Org. Lett.* **2019**, *21*, 3053−3056

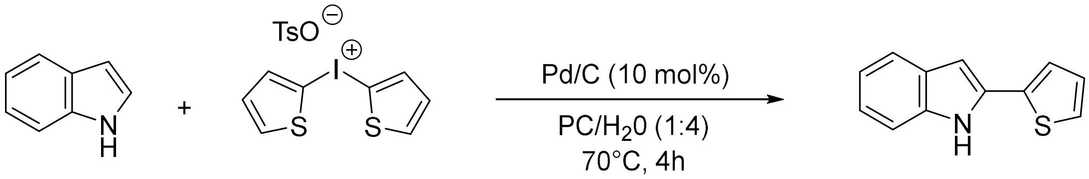

1a **2i** **3r**

Mol. Wt.: 199.271

METHOD:
In a screw capped vial equipped with a magnetic stirrer, with indole (**1a**) (0.2 mmol, 23.4 mg), Pd/C (10 mol%, 21.3 mg), 1 mL of PC/H_2O (1:4) and di(thien-2-yl)iodonium tosylate (**2i**) (0.25 mmol, 116 mg) were consecutively added and the resulting mixture was left under stirring at 70°C. After 4h the reaction mixture was left to cool to room temperature, then centrifuged to separate and recover the medium, PC/H_2O. The solid residue was then washed with hot ethyl acetate (2x0.5 mL), which was recovered as supernatant after centrifugation (6500 rpm, 15 min). The combined organic layers were concentred under reduced pressure and the crude oil was purified by flash chromatography on silica gel (PE→PE/EtOAc 9:1) and the obtained solid was washed with cold PE (5 x 1 mL) to remove traces of residual indole, to afford the title compound (**3r**) as a pale yellow solid, 18 mg (45%).

Mol Formula	$C_{12}H_9NS$	m.p.	160-161°C

	δ value	No. H	Mult.	j value/Hz
**^{1}H NMR 400 MHz $CDCl_3$**	6.74	1	*d*	1.0
	7.10	1	*dd*	4.9; 3.8
	7.13	1	t	7.5
	7.20	1	t	7.4
	7.27	1	*d*	3.7
	7.29	1	*d*	5.1
	7.37	1	*d*	8.0
	7.60	1	*d*	7.8
	8.20	1	*bs*	

^{13}C NMR (100.6 MHz, $CDCl_3$) δ: 100.6, 110.9, 120.6, 120.7, 122.7, 123.1, 124.7, 128.0, 129.2, 132.5, 135.8, 136.6

GC-EIMS (m/z, %): 198 (30), 199 (100), 200 (28)

Chem. Name	3-Methyl-2-phenyl-1*H*-indole (3s)
**Lit. Ref.**	*RSC Adv.*, **2015**, *5*, 70329-70332

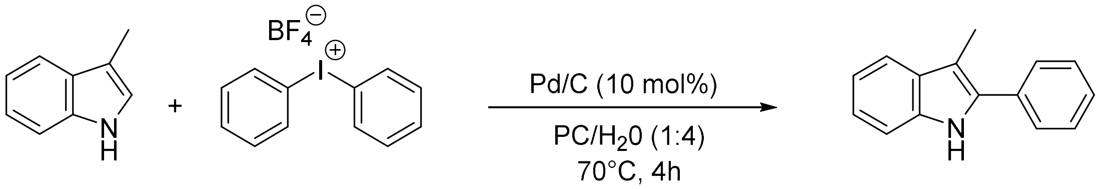

1l **2a** **3s**

Mol. Wt.: 207.28

METHOD:
In a screw capped vial equipped with a magnetic stirrer, indole (**1l**) (0.2 mmol, 26.23 mg), Pd/C (10 mol%, 21.3 mg), 1 mL of PC/H_2O (1:4) and diphenyliodonium tetrafluoroborate (**2a**) (0.25 mmol, 92 mg) were consecutively added and the resulting mixture was left under stirring at 70°C. After 4 h the reaction mixture was left to cool to room temperature, then centrifuged to separate and recover the medium, PC/H_2O. The solid residue was then washed with hot ethyl acetate (2x0.5 mL), which was recovered as supernatant after centrifugation (6500 rpm, 15 min). The combined organic layers were concentred under reduced pressure and the crude oil purified by flash column chromatography on activated neutral alumina gel (PE/EtOAc 9:1 → 8:2) to afford the title compound (**3s**) as a white solid, 8 mg (20%).

Mol Formula	$C_{15}H_{13}N$	m.p.	91.5 °C

	δ value	No. H	Mult.	j value/Hz
**^{1}H NMR**	2.47	3	*s*	
**400 MHz**	7.06	1	*t*	7.4
**Acetone-*d*$_6$**	7.13	1	*t*	7.5
	7.35	1	*t*	7.4
	7.40	1	*d*	8.0
	7.49	2	*t*	7.8
	7.57	1	*d*	7.8
	7.71-7.69	2	*m*	
	10.25	1	*bs*	

^{13}C NMR (100.6 MHz, Acetone-*d*$_6$) δ : 10.0, 108.3, 111.8, 119.4, 119.7, 122.6, 127.9, 128.5, 129.5, 130.9, 134.5, 134.9, 137.3

GC-EIMS (m/z, %): 130 (24), 206 (96), 207 (100)

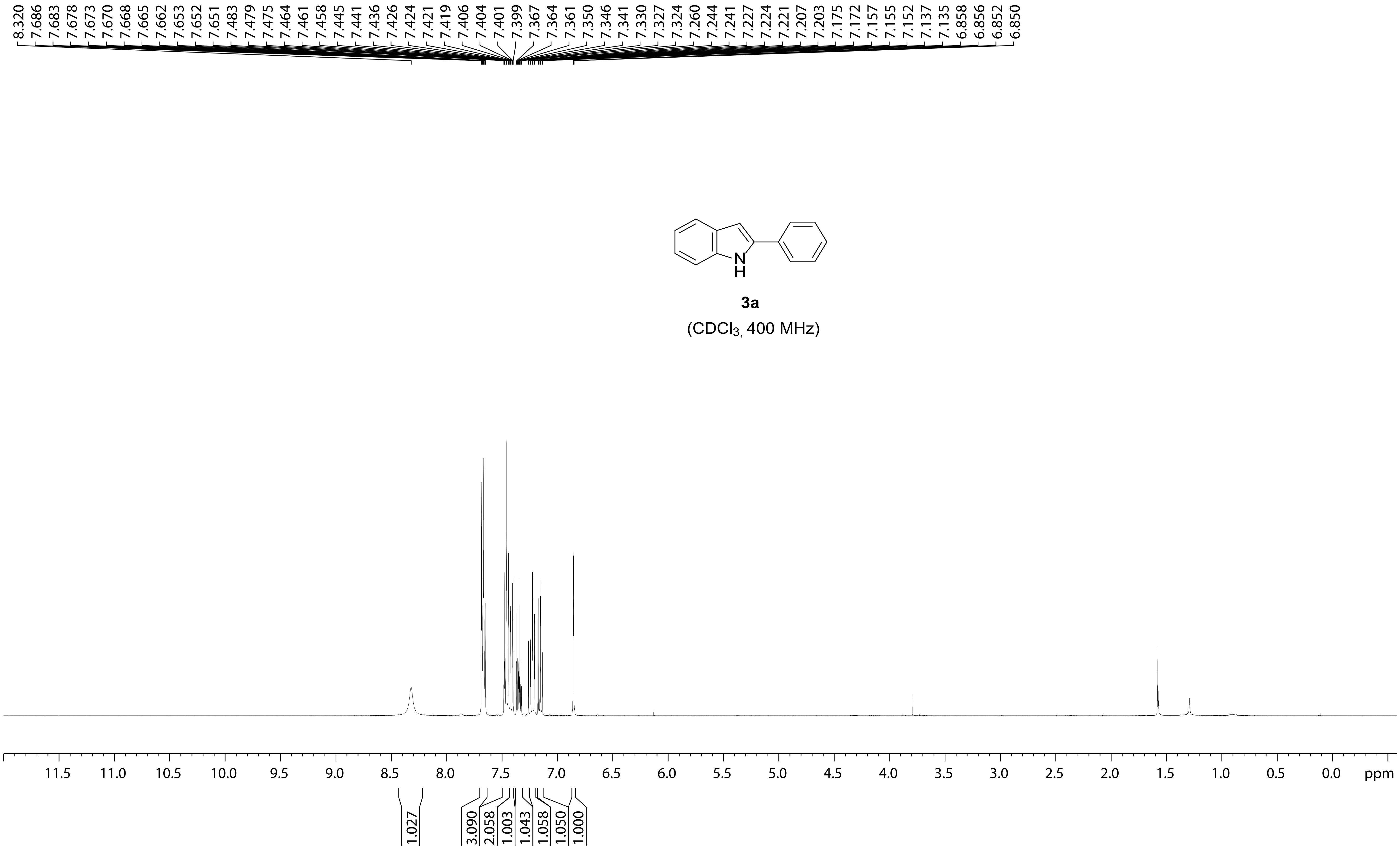

3a

($CDCl_3$, 400 MHz)

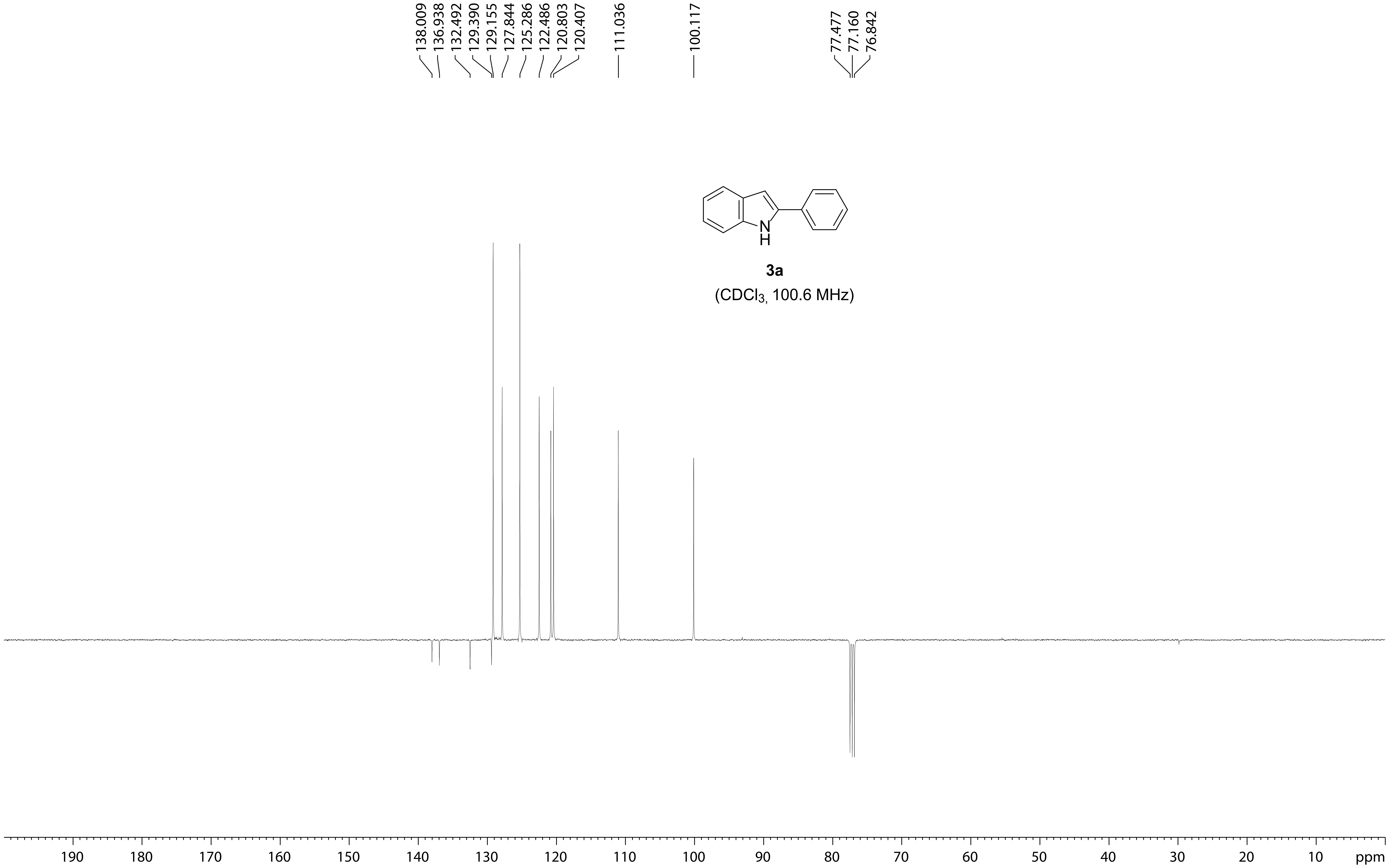

3a

($CDCl_3$, 100.6 MHz)

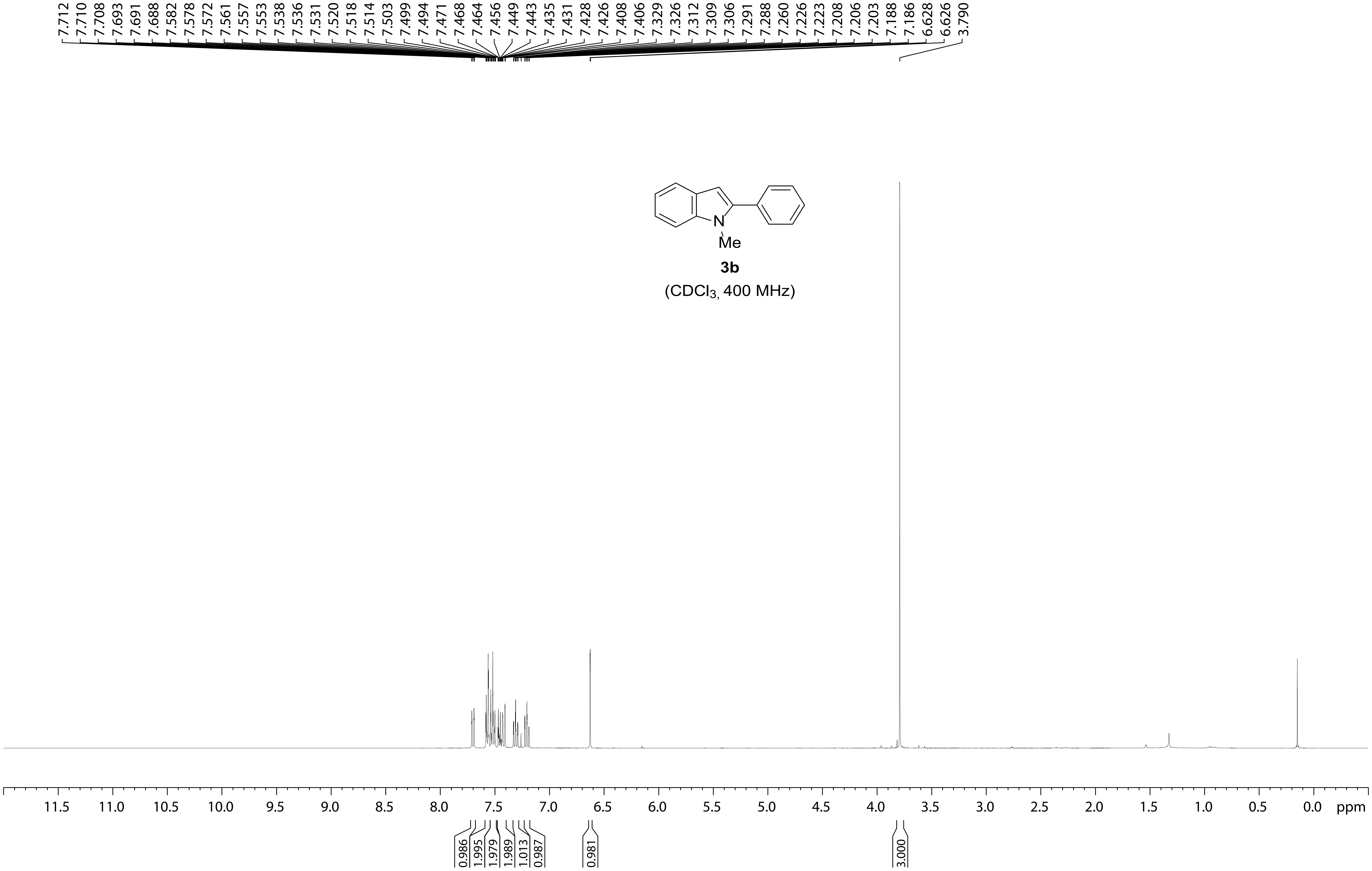

3b

($CDCl_3$, 400 MHz)

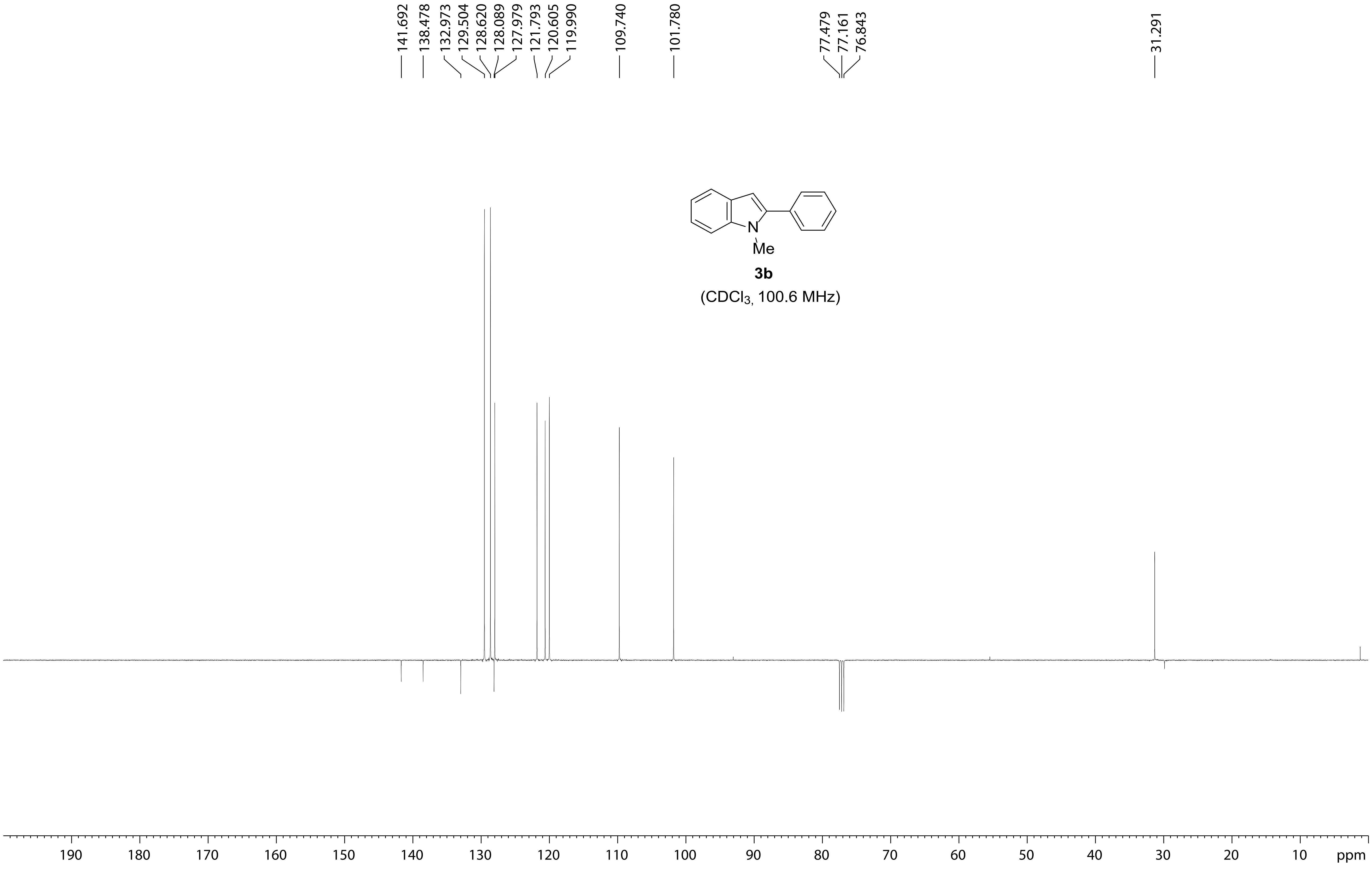

3b

($CDCl_3$, 100.6 MHz)

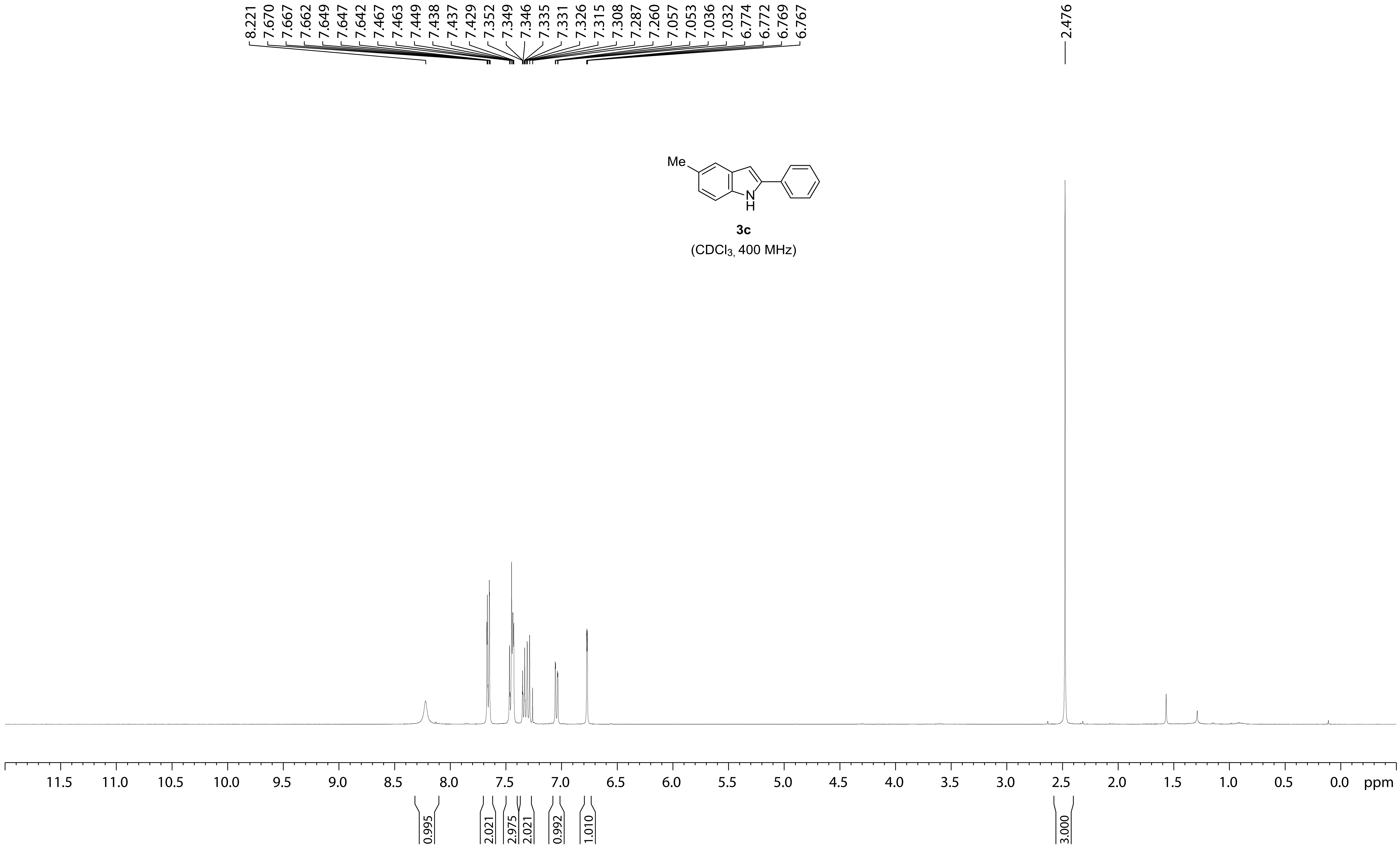

3c

($CDCl_3$, 400 MHz)

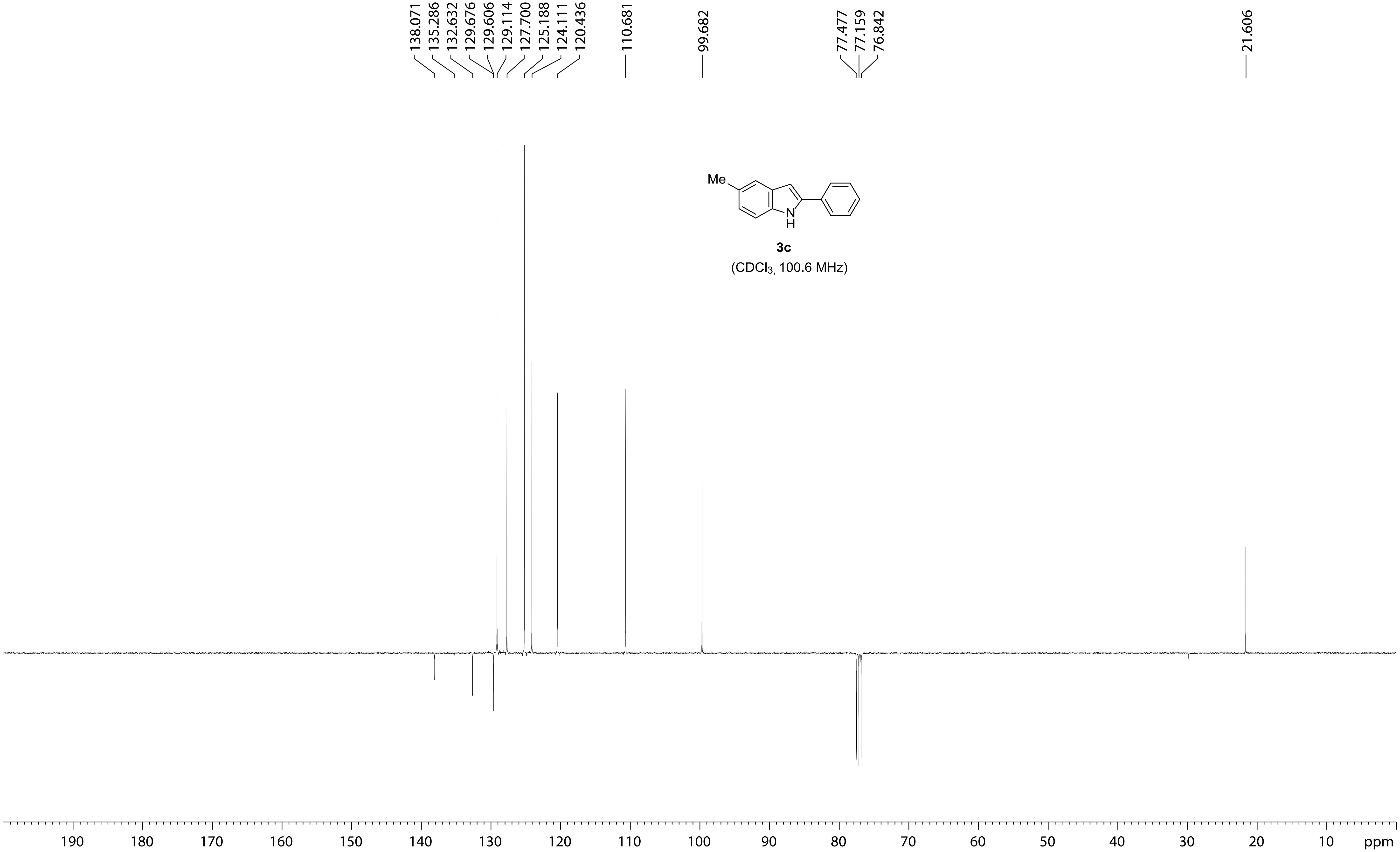

3c

($CDCl_3$, 100.6 MHz)

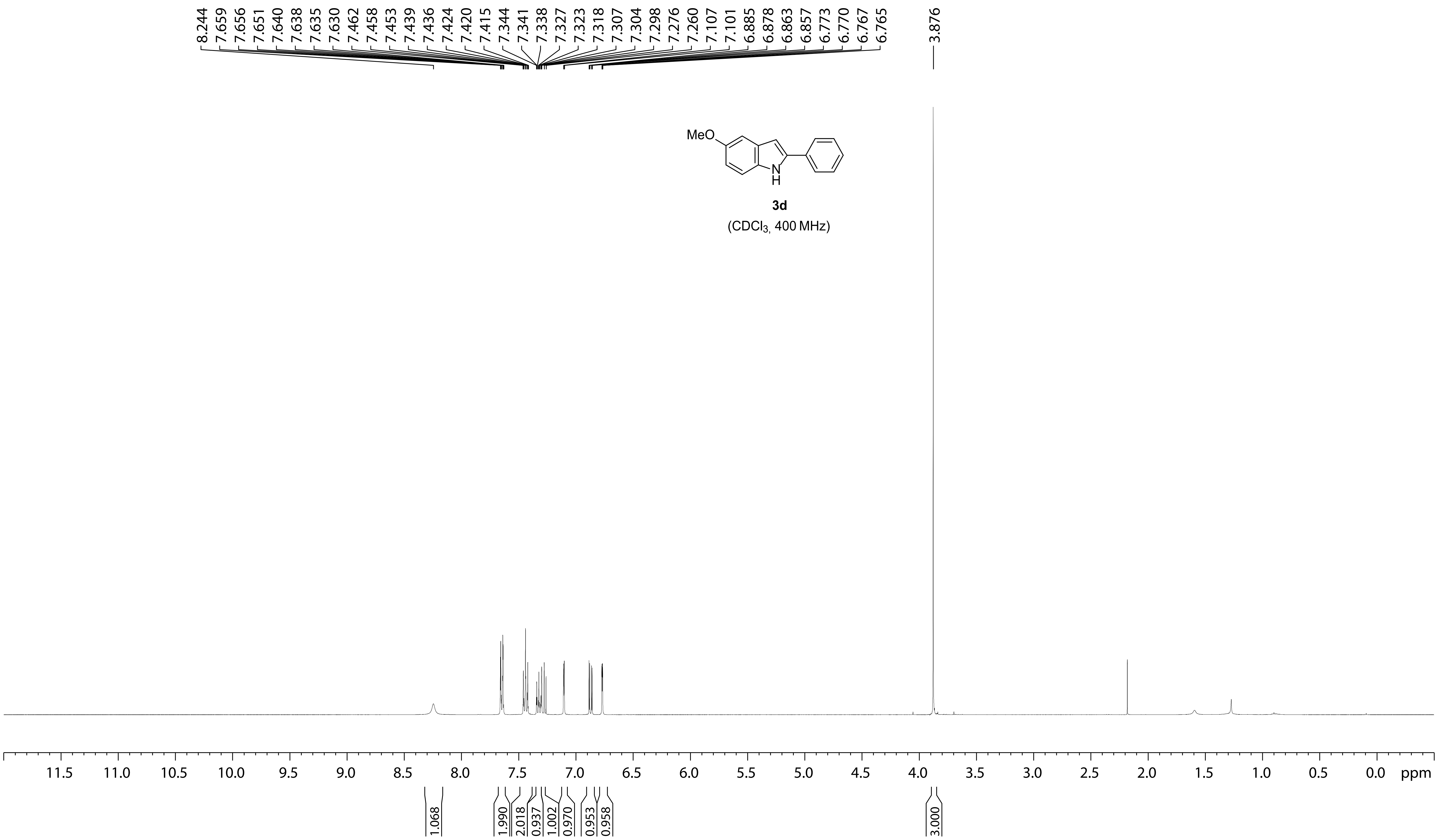
8.244
7.659
7.656
7.651
7.640
7.638
7.635
7.630
7.462
7.458
7.453
7.439
7.436
7.424
7.420
7.415
7.344
7.341
7.338
7.327
7.323
7.318
7.307
7.304
7.298
7.276
7.260
7.107
7.101
6.885
6.878
6.863
6.857
6.773
6.770
6.767
6.765
3.876
MeO
N
H
3d
(CDCl3, 400 MHz)
11.5 11.0 10.5 10.0 9.5 9.0 8.5 8.0 7.5 7.0 6.5 6.0 5.5 5.0 4.5 4.0 3.5 3.0 2.5 2.0 1.5 1.0 0.5 0.0 ppm
1.068
1.990
2.018
0.937
1.002
0.970
0.953
0.958
3.000

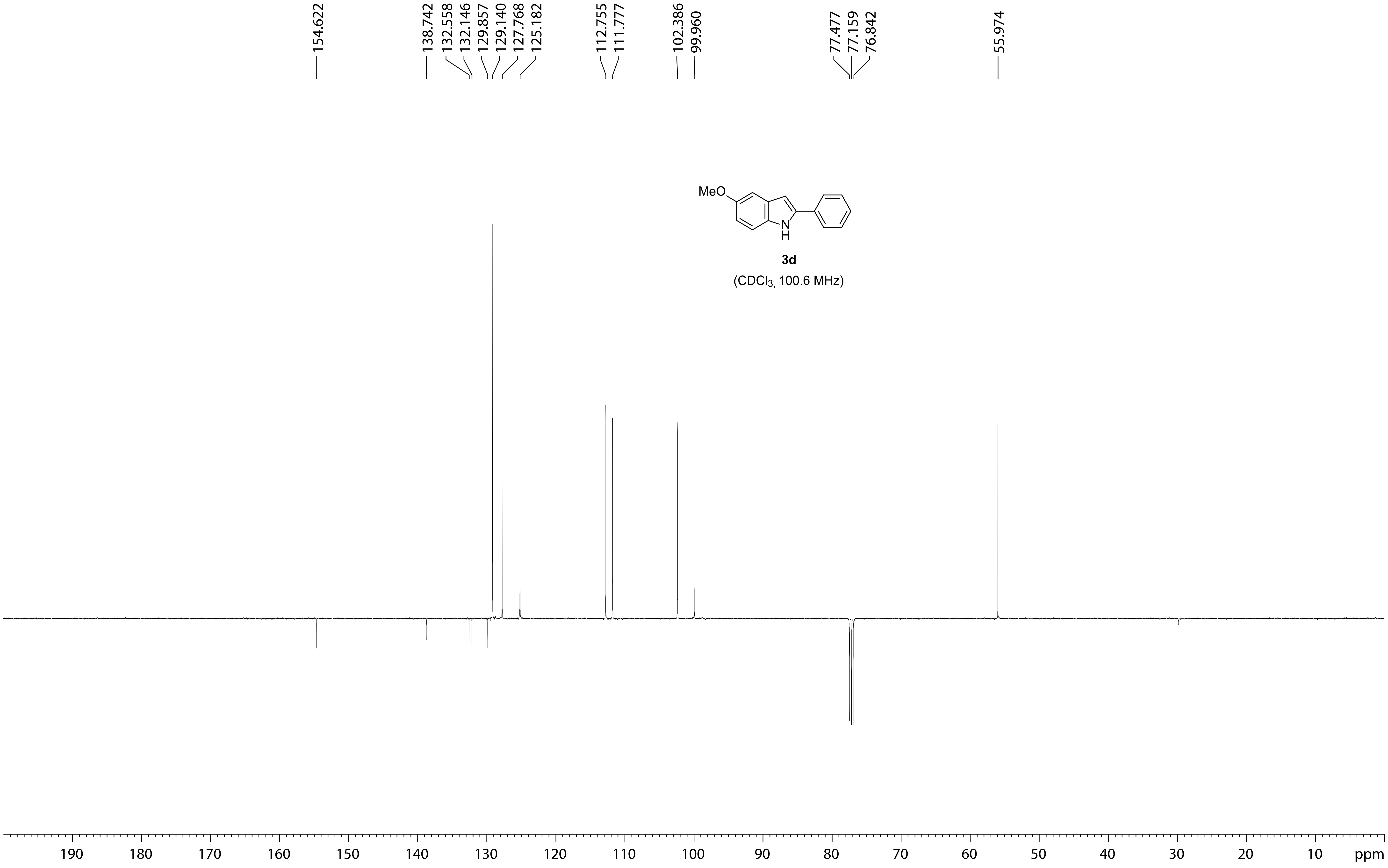

3d

($CDCl_3$, 100.6 MHz)

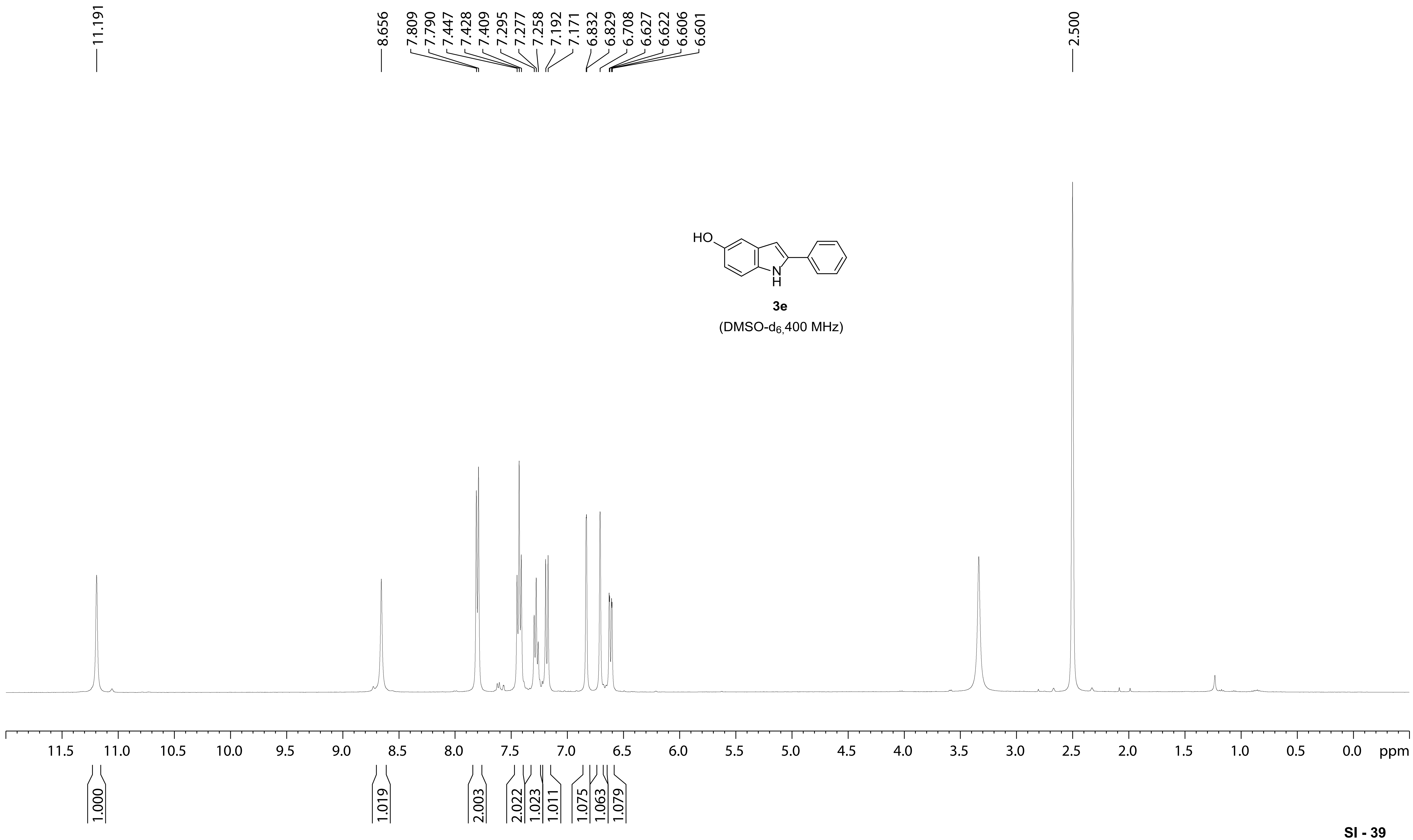
11.191
8.656
7.809
7.790
7.447
7.428
7.409
7.295
7.277
7.258
7.192
7.171
6.832
6.829
6.708
6.627
6.622
6.606
6.601
2.500
HO
N
H
3e
(DMSO-d6,400 MHz)
11.5 11.0 10.5 10.0 9.5 9.0 8.5 8.0 7.5 7.0 6.5 6.0 5.5 5.0 4.5 4.0 3.5 3.0 2.5 2.0 1.5 1.0 0.5 0.0 ppm
1.000
1.019
2.003
2.022
1.023
1.011
1.075
1.063
1.079

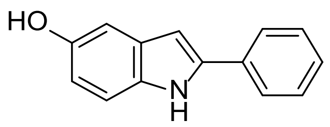

3e

(DMSO-d_6, 100.6 MHz)

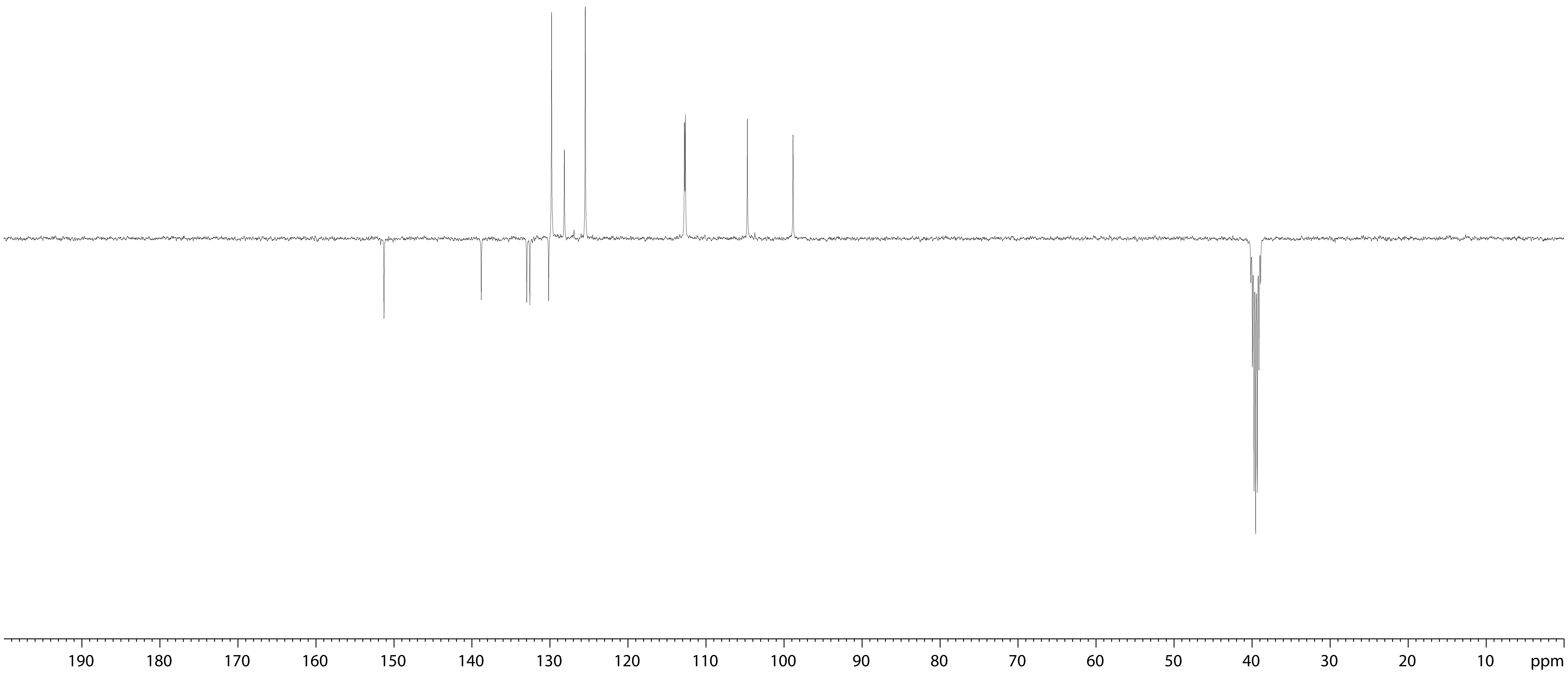

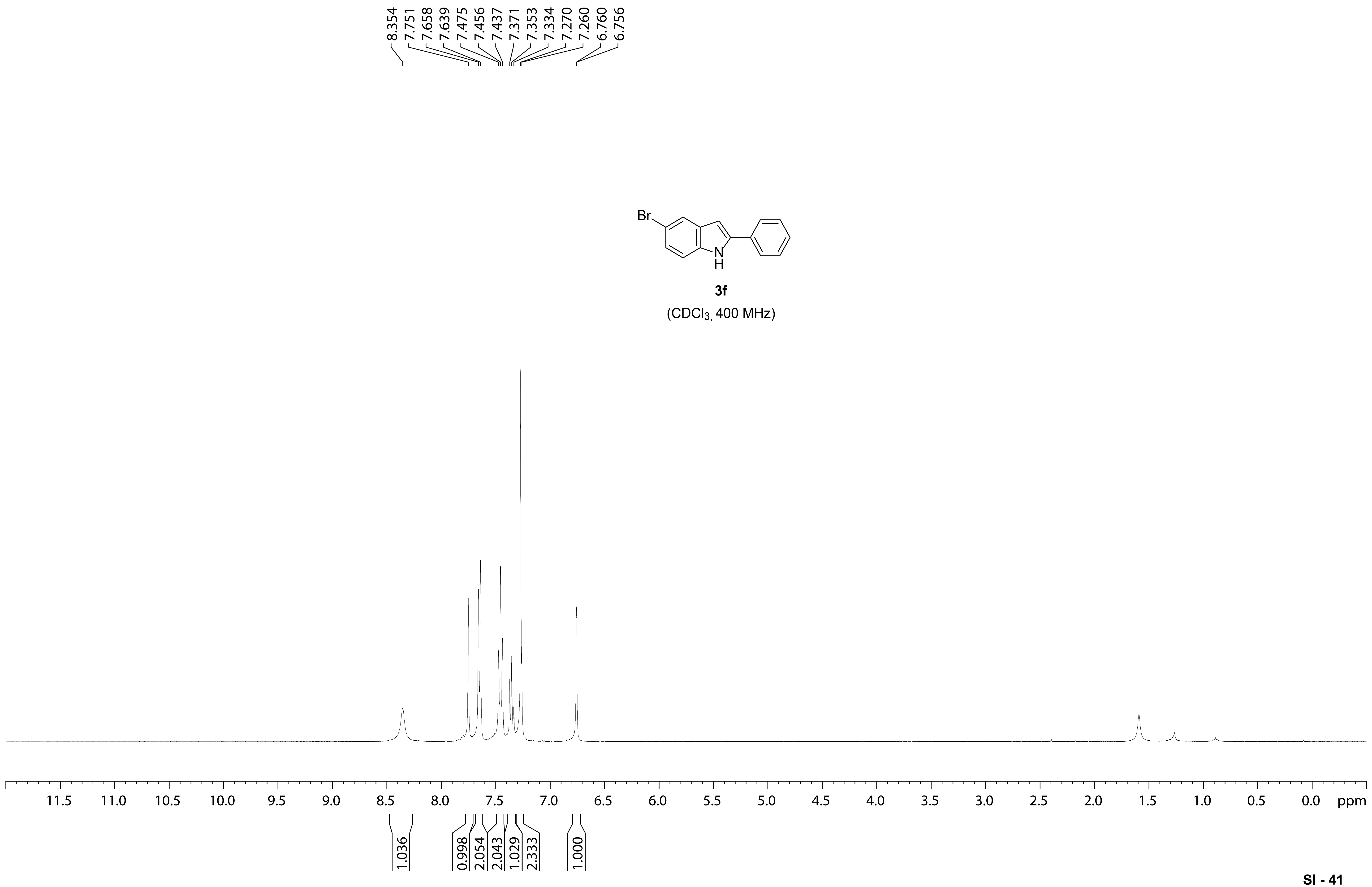
8.354
7.751
7.658
7.639
7.475
7.456
7.437
7.371
7.353
7.334
7.270
7.260
6.760
6.756
Br
N
H
3f
(CDCl3, 400 MHz)
11.5 11.0 10.5 10.0 9.5 9.0 8.5 8.0 7.5 7.0 6.5 6.0 5.5 5.0 4.5 4.0 3.5 3.0 2.5 2.0 1.5 1.0 0.5 0.0 ppm
1.036
0.998
2.054
2.043
1.029
2.333
1.000

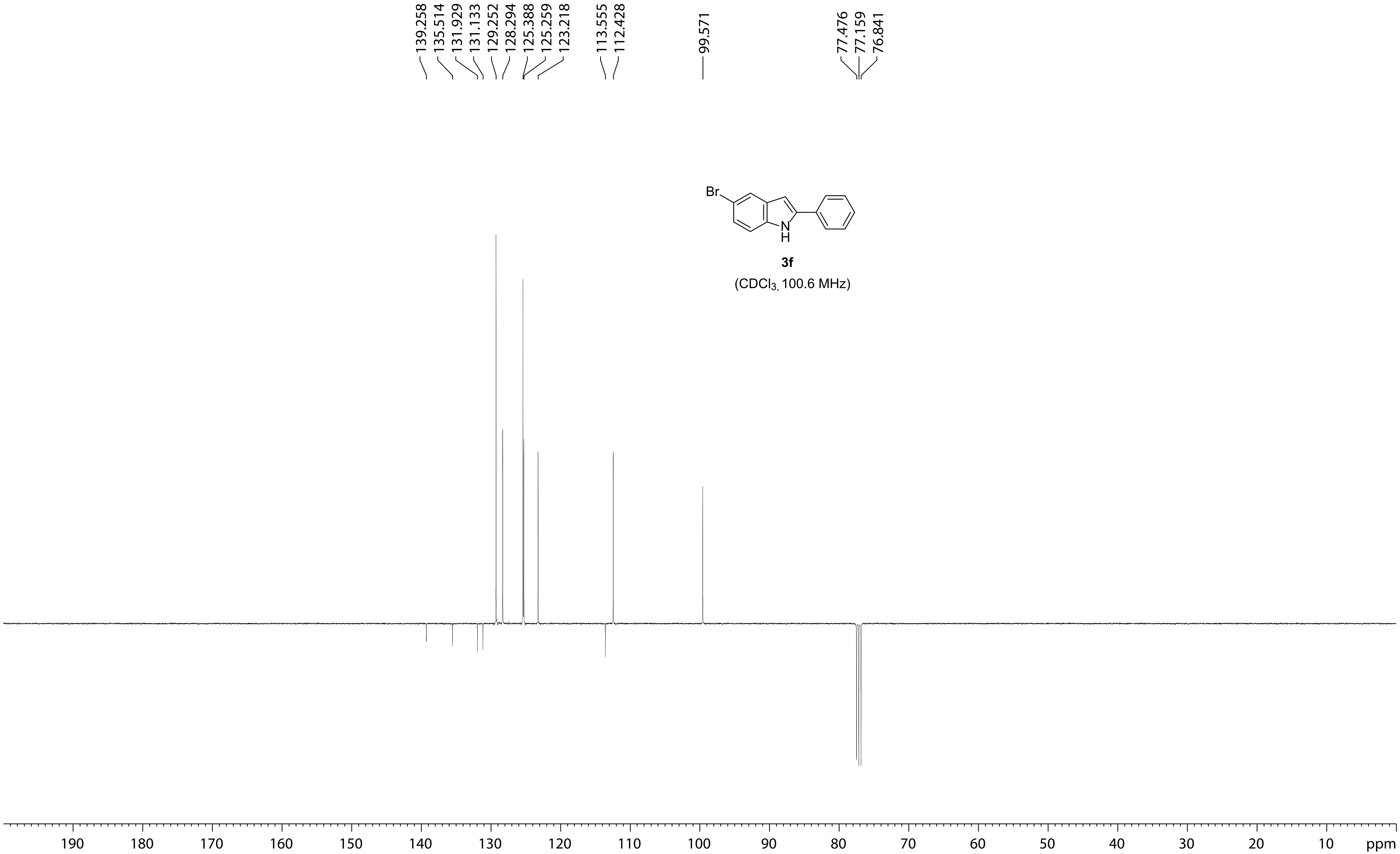
139.258
135.514
131.929
131.133
129.252
128.294
125.388
125.259
123.218
113.555
112.428
99.571
77.476
77.159
76.841
Br
N
H
3f
(CDCl$_3$, 100.6 MHz)
190 180 170 160 150 140 130 120 110 100 90 80 70 60 50 40 30 20 10 ppm

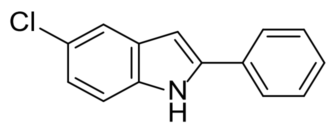

3g
($CDCl_3$, 400 MHz)

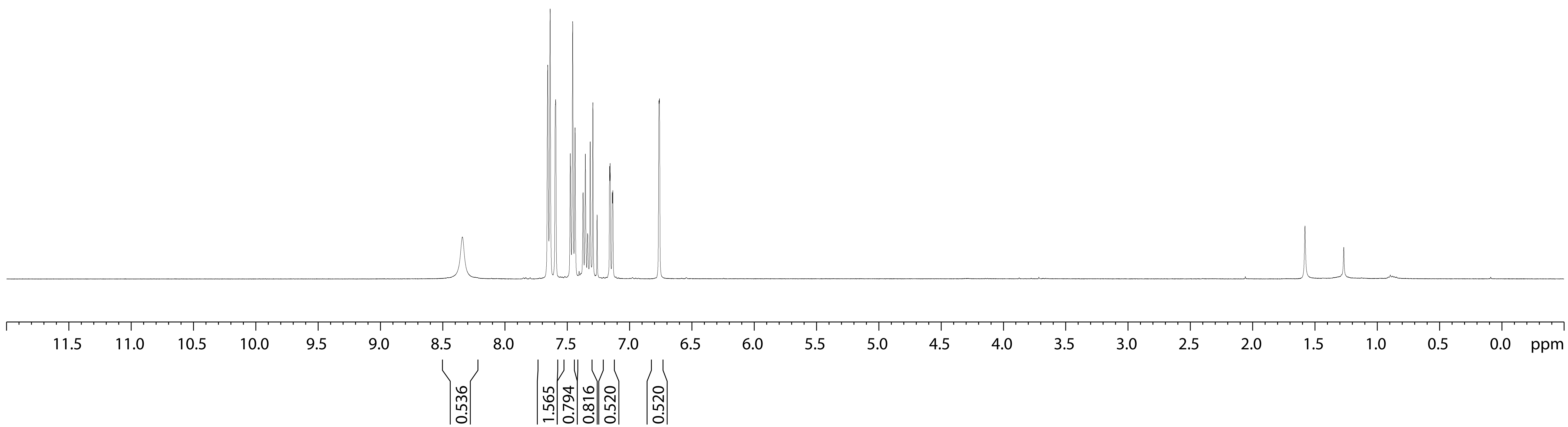

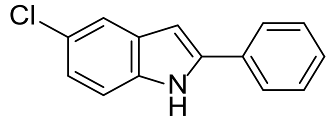

3g
($CDCl_3$, 100.6 MHz)

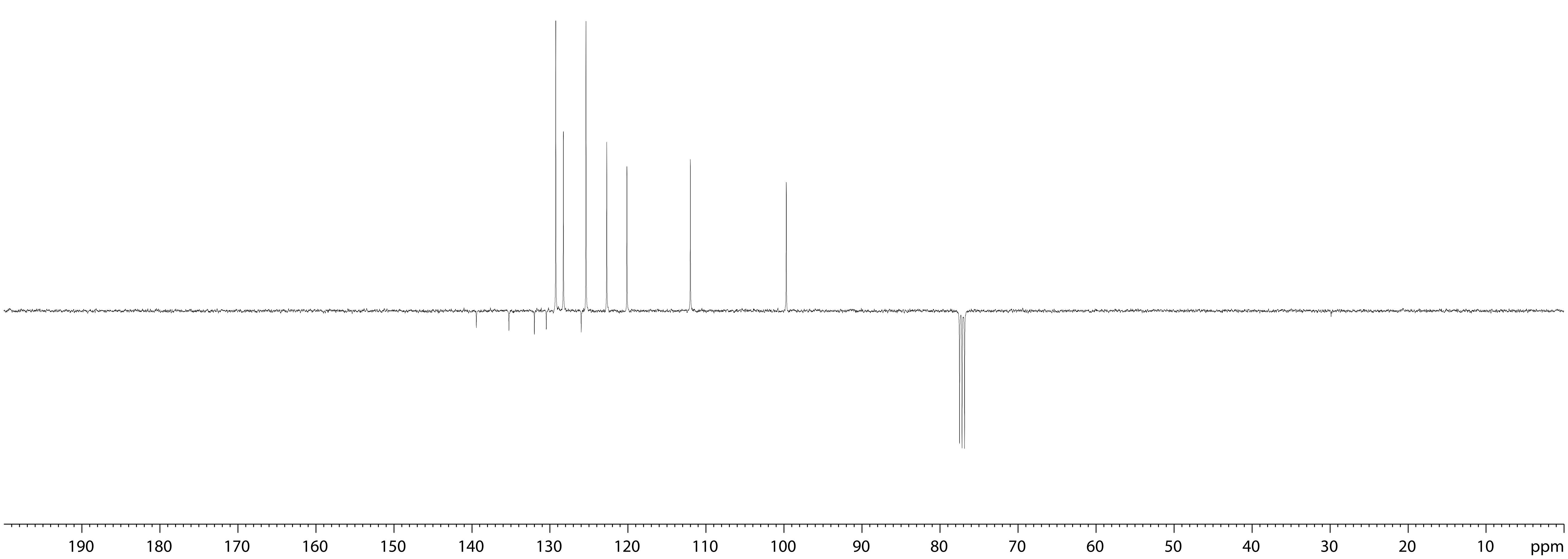

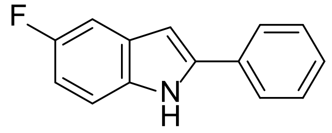

3h

($CDCl_3$, 400 MHz)

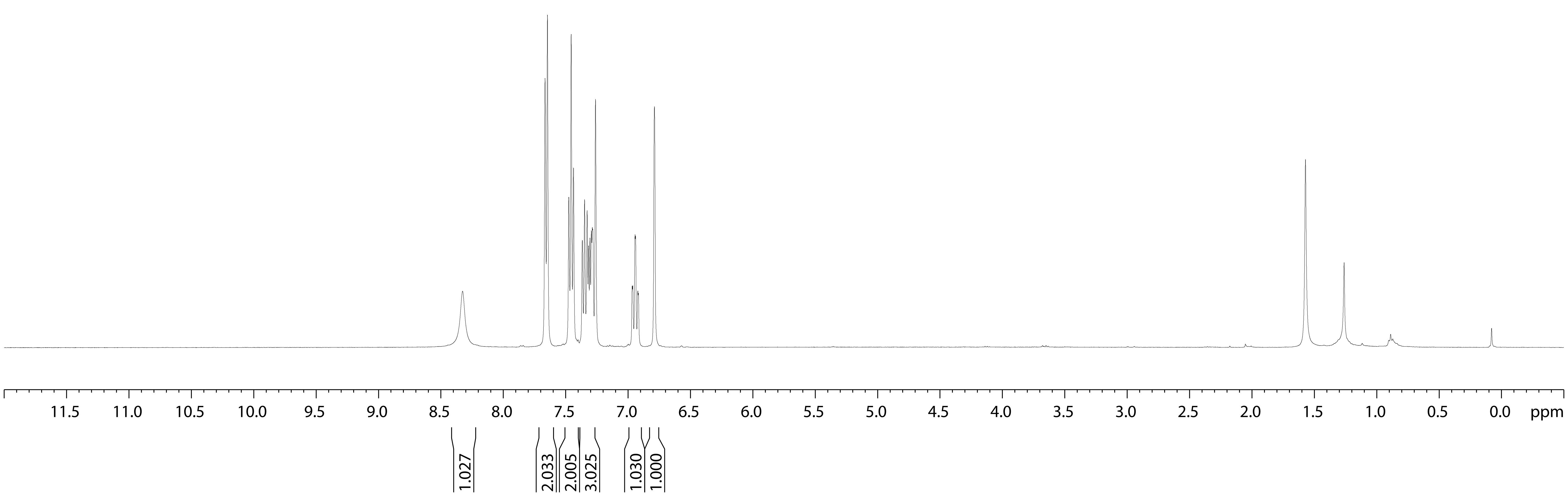

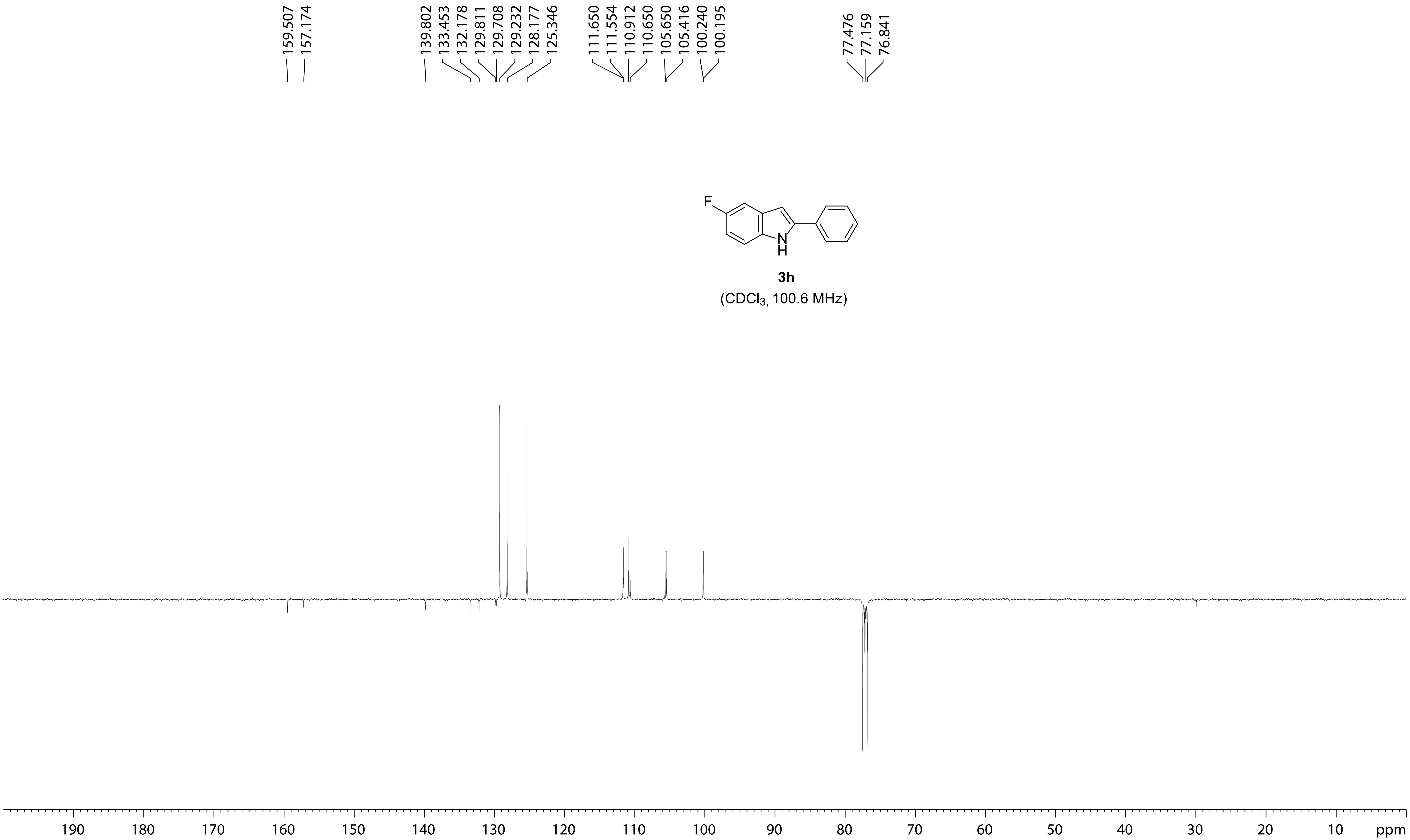
159.507
157.174
139.802
133.453
132.178
129.811
129.708
129.232
128.177
125.346
111.650
111.554
110.912
110.650
105.650
105.416
100.240
100.195
77.476
77.159
76.841
F
N
H
3h
(CDCl$_3$, 100.6 MHz)
190 180 170 160 150 140 130 120 110 100 90 80 70 60 50 40 30 20 10 ppm

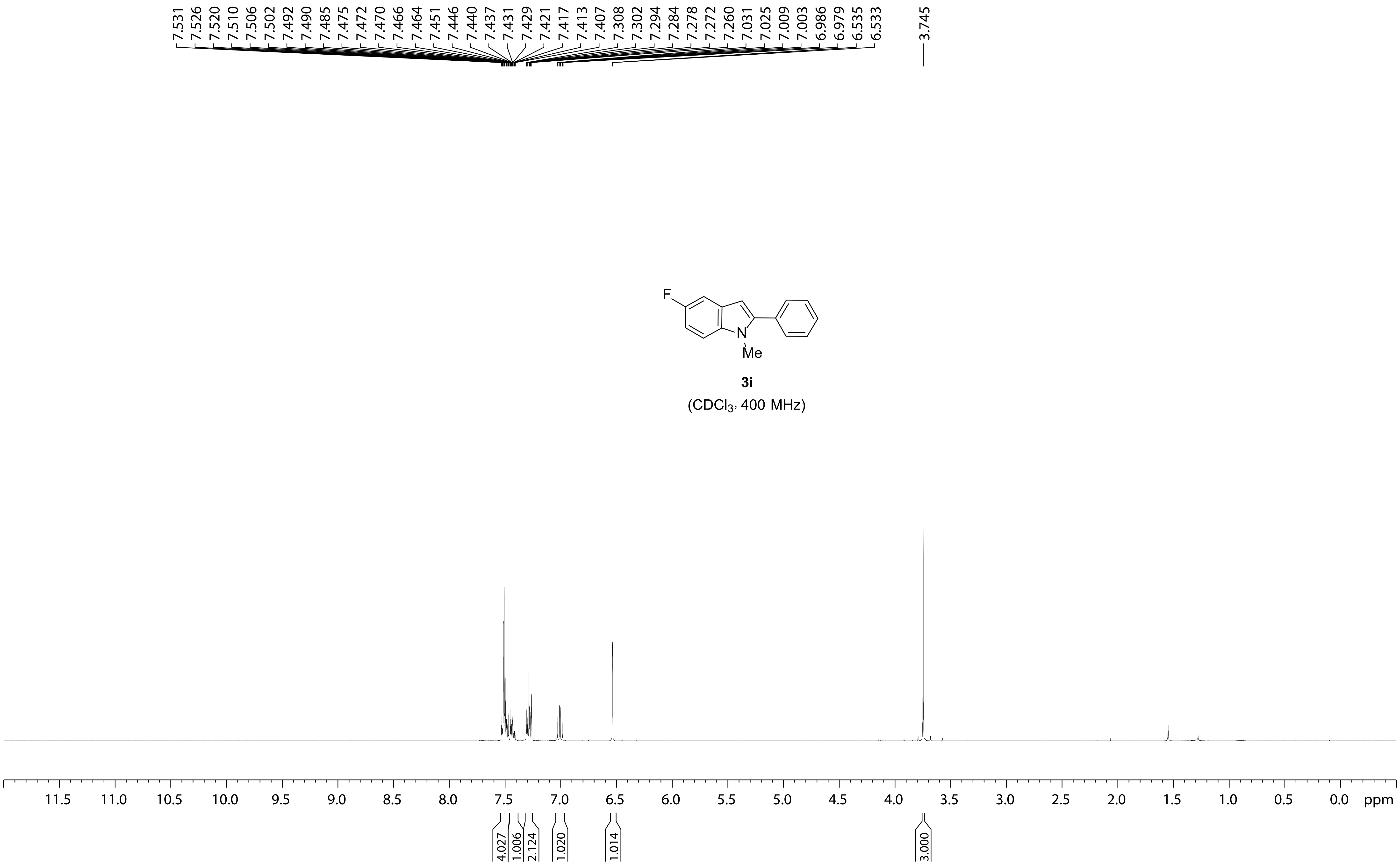
7.531
7.526
7.520
7.510
7.506
7.502
7.492
7.490
7.485
7.475
7.472
7.470
7.466
7.464
7.451
7.446
7.440
7.437
7.431
7.429
7.421
7.417
7.413
7.407
7.308
7.302
7.294
7.284
7.278
7.272
7.260
7.031
7.025
7.009
7.003
6.986
6.979
6.535
6.533
3.745
F
N
Me
3i
(CDCl$_3$, 400 MHz)
11.5 11.0 10.5 10.0 9.5 9.0 8.5 8.0 7.5 7.0 6.5 6.0 5.5 5.0 4.5 4.0 3.5 3.0 2.5 2.0 1.5 1.0 0.5 0.0 ppm
4.027
1.006
2.124
1.020
1.014
3.000

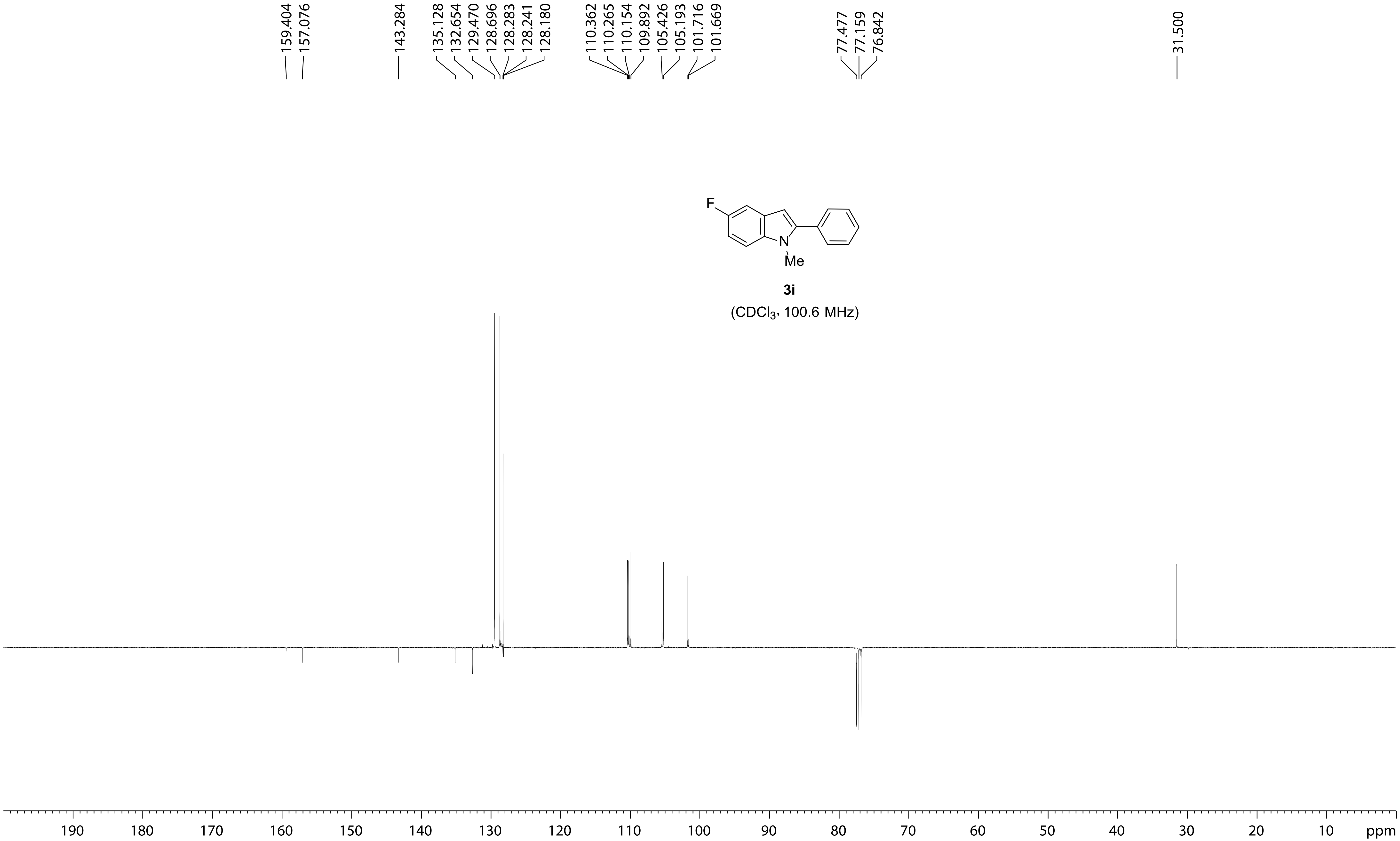
159.404
157.076
143.284
135.128
132.654
129.470
128.696
128.283
128.241
128.180
110.362
110.265
110.154
109.892
105.426
105.193
101.716
101.669
77.477
77.159
76.842
31.500
F
N
Me
3i
($CDCl_3$, 100.6 MHz)
190
180
170
160
150
140
130
120
110
100
90
80
70
60
50
40
30
20
10
ppm

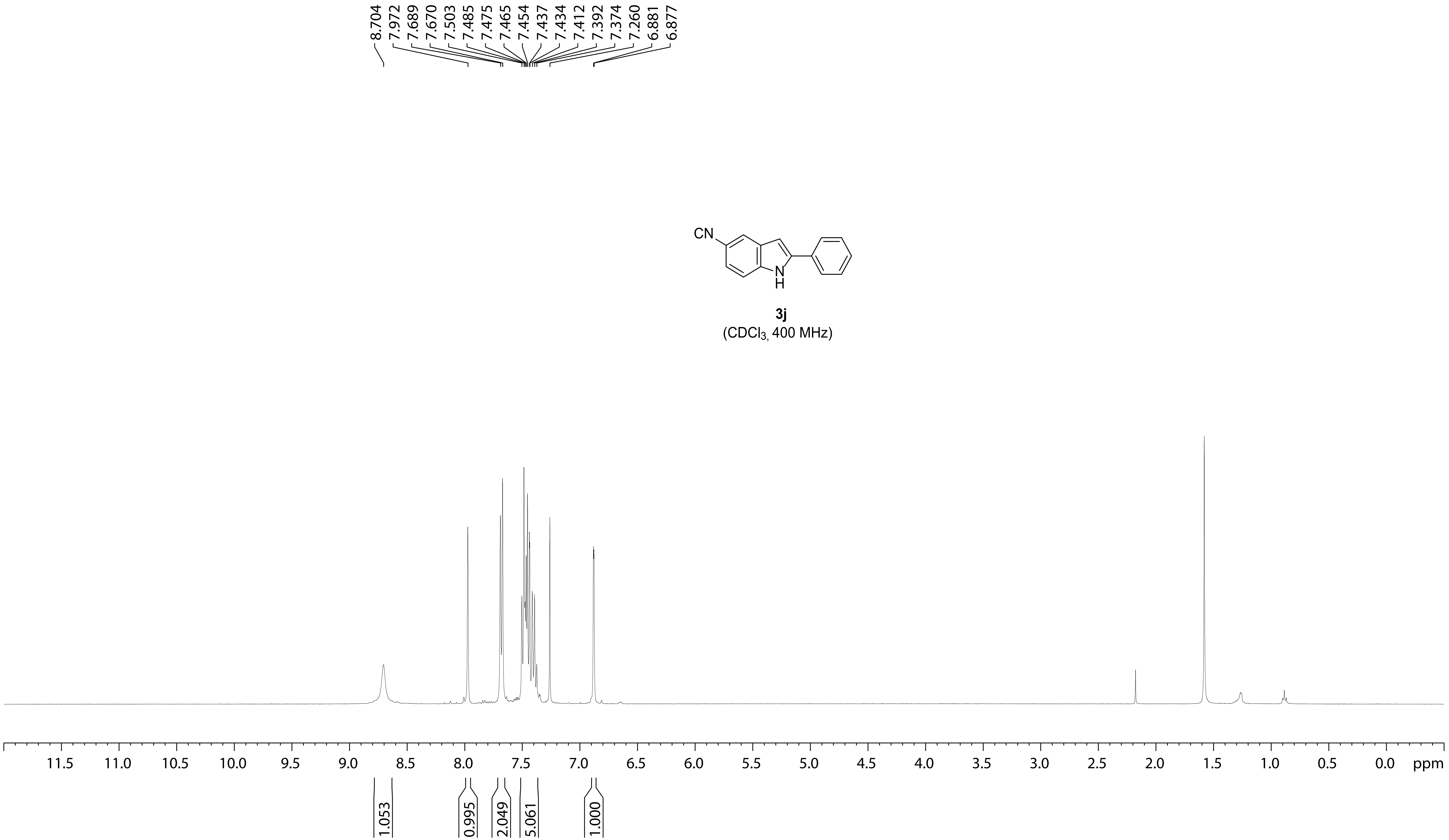

3j
($CDCl_3$, 400 MHz)

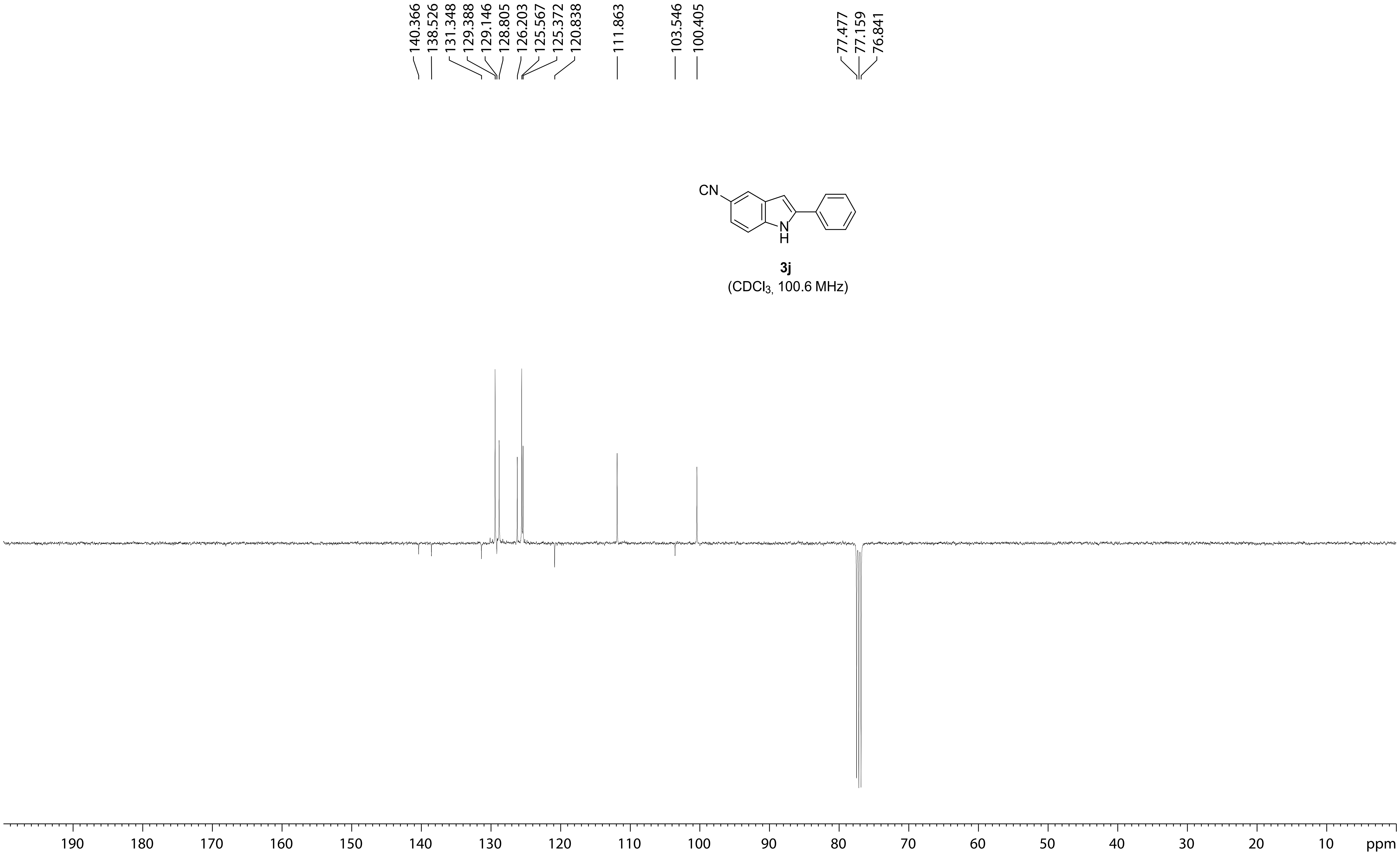

3j
($CDCl_3$, 100.6 MHz)

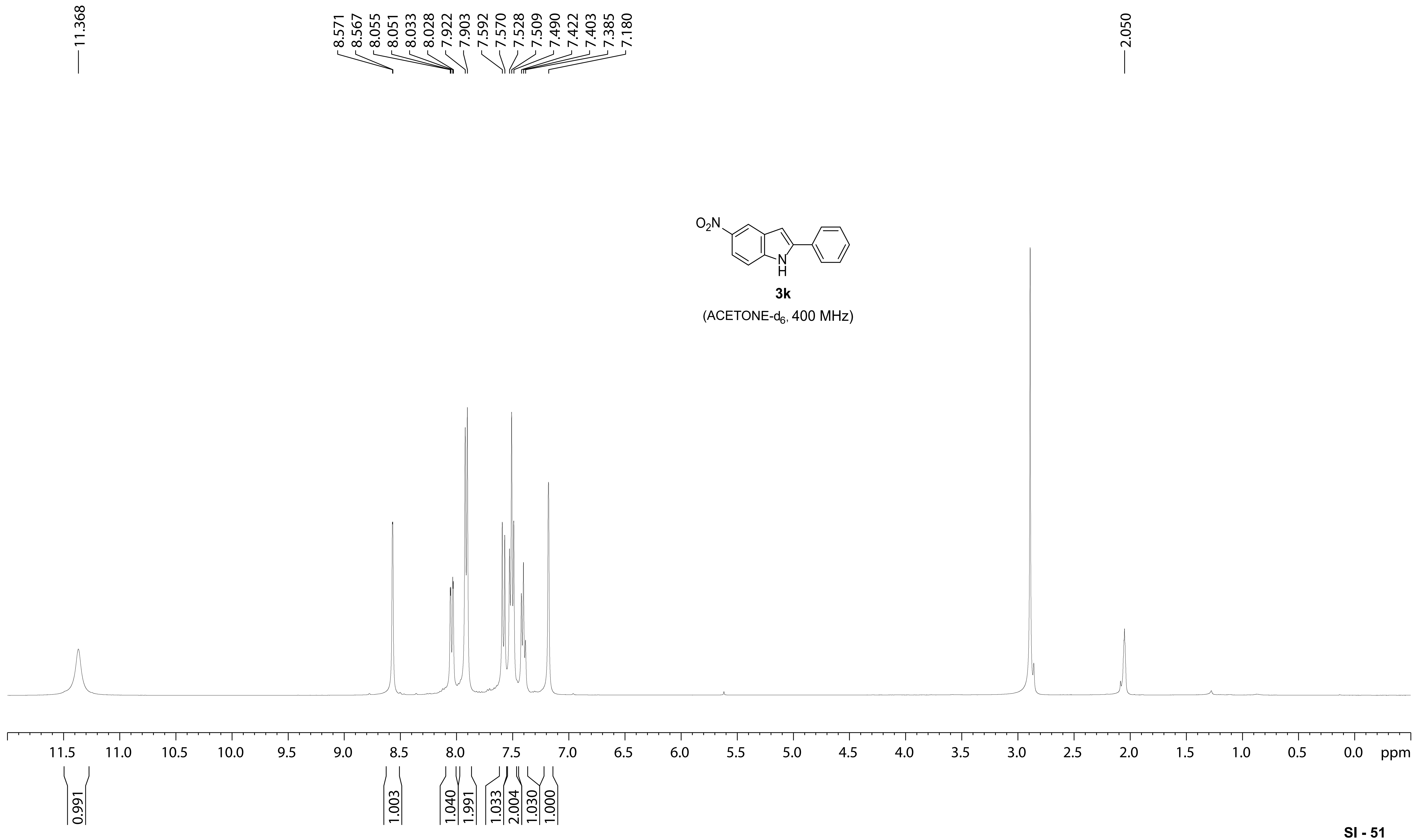

3k

(ACETONE-d_6, 400 MHz)

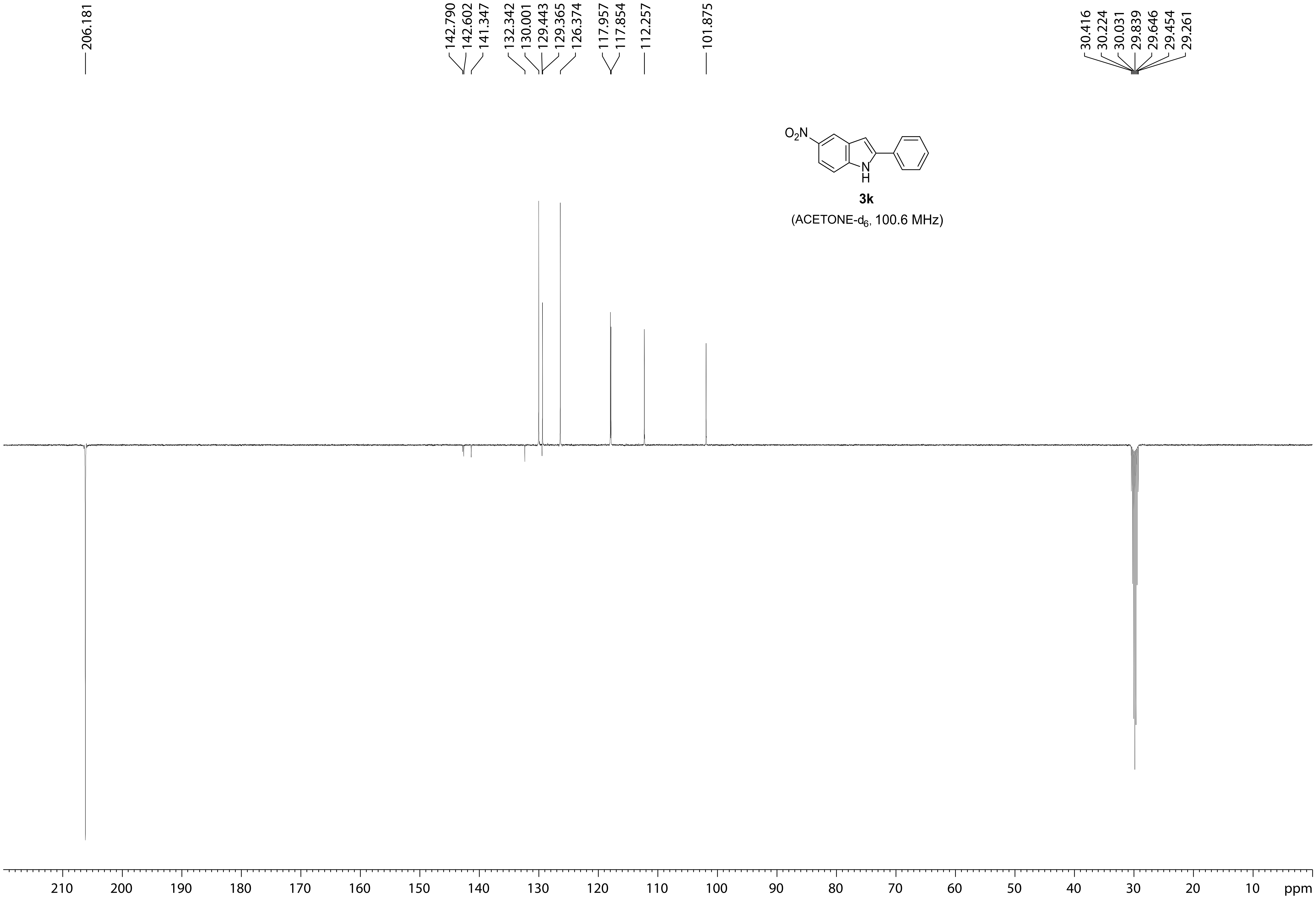
206.181
142.790
142.602
141.347
132.342
130.001
129.443
129.365
126.374
117.957
117.854
112.257
101.875
30.416
30.224
30.031
29.839
29.646
29.454
29.261
O2N
N
H
3k
(ACETONE-d6, 100.6 MHz)
210 200 190 180 170 160 150 140 130 120 110 100 90 80 70 60 50 40 30 20 10 ppm

2-p-TOLILINDOLO IN CDCl3

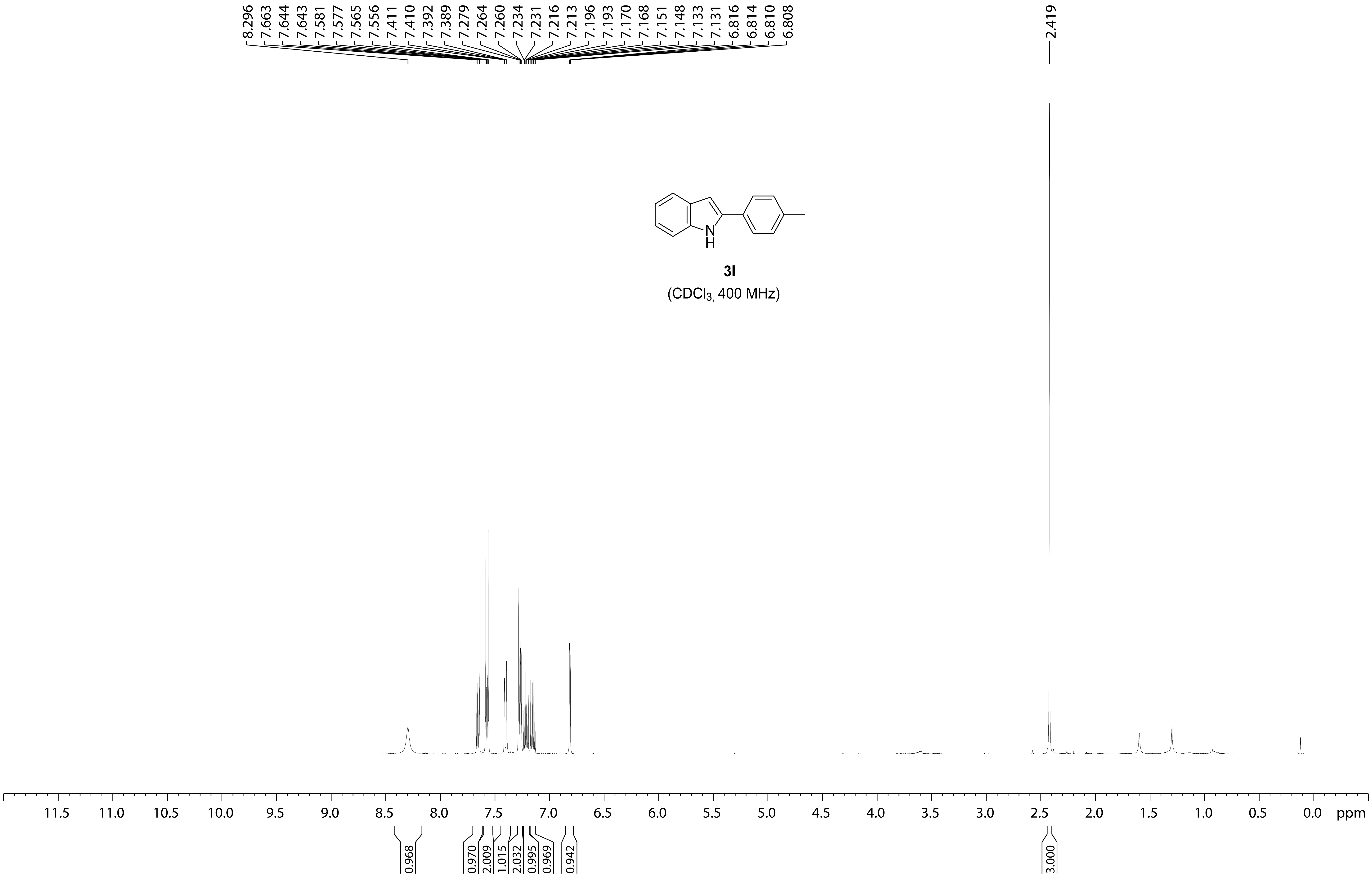

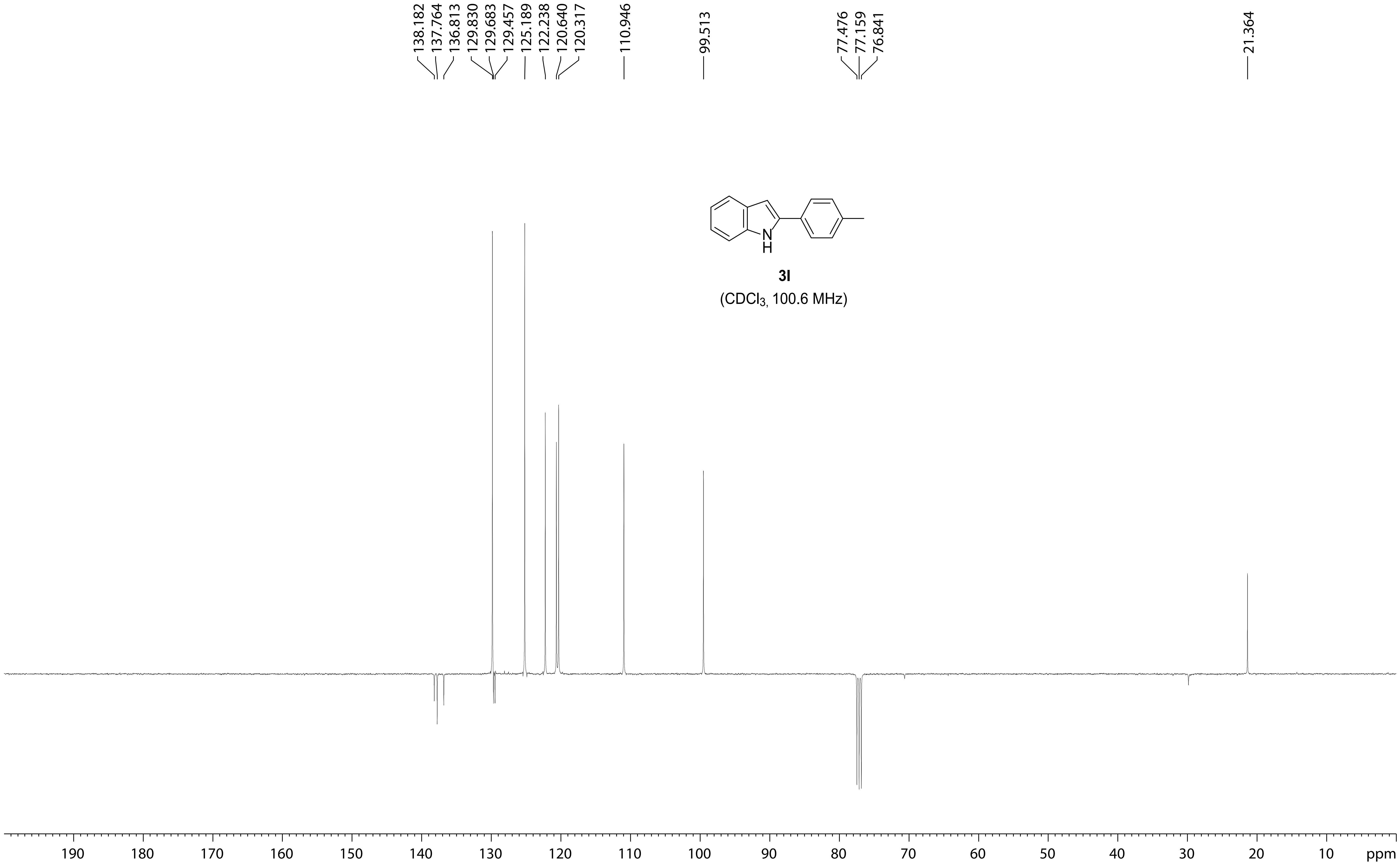

3l

($CDCl_3$, 100.6 MHz)

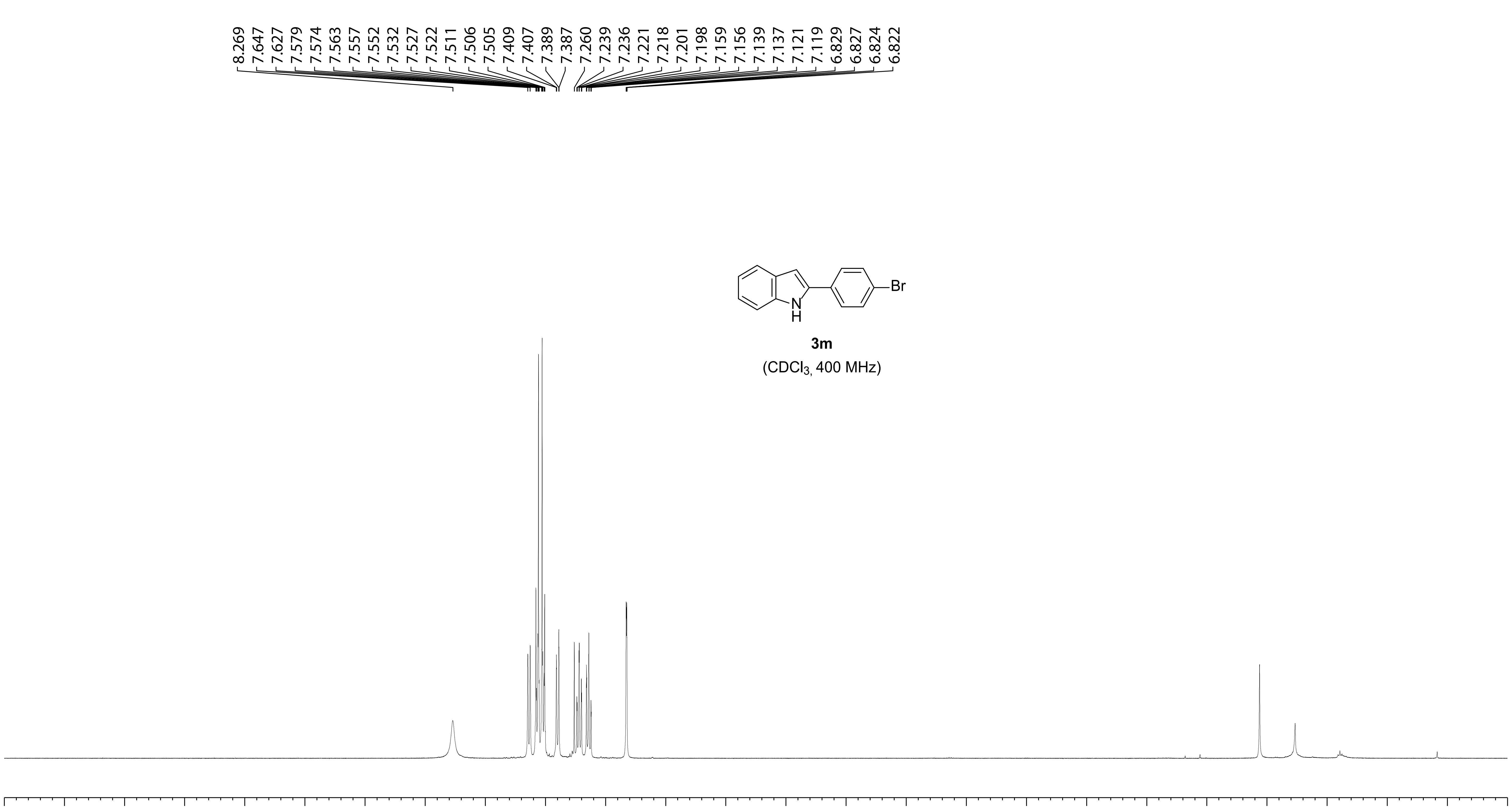

3m

($CDCl_3$, 400 MHz)

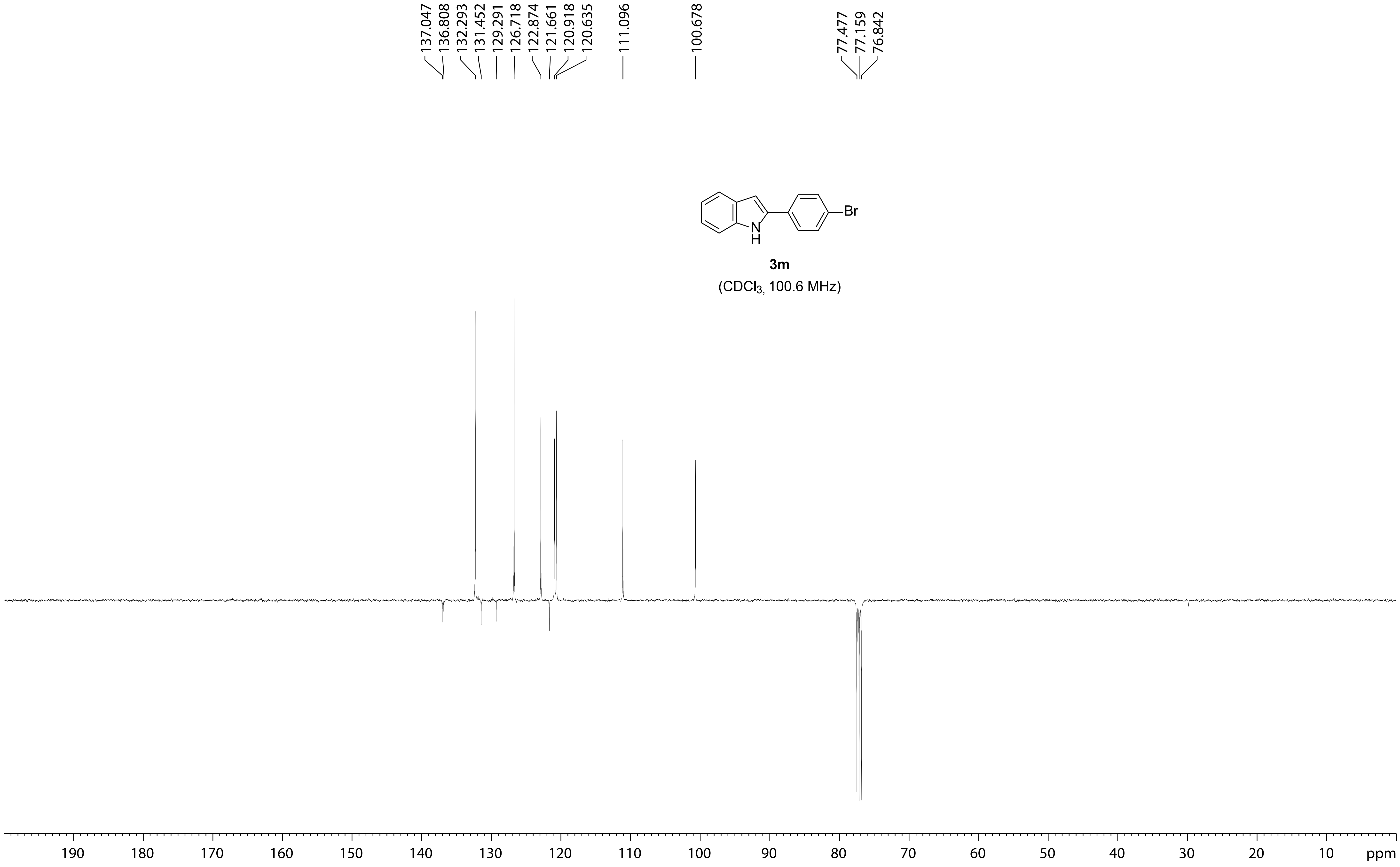
137.047
136.808
132.293
131.452
129.291
126.718
122.874
121.661
120.918
120.635
111.096
100.678
77.477
77.159
76.842
Br
N
H
3m
(CDCl$_3$, 100.6 MHz)
190 180 170 160 150 140 130 120 110 100 90 80 70 60 50 40 30 20 10 ppm

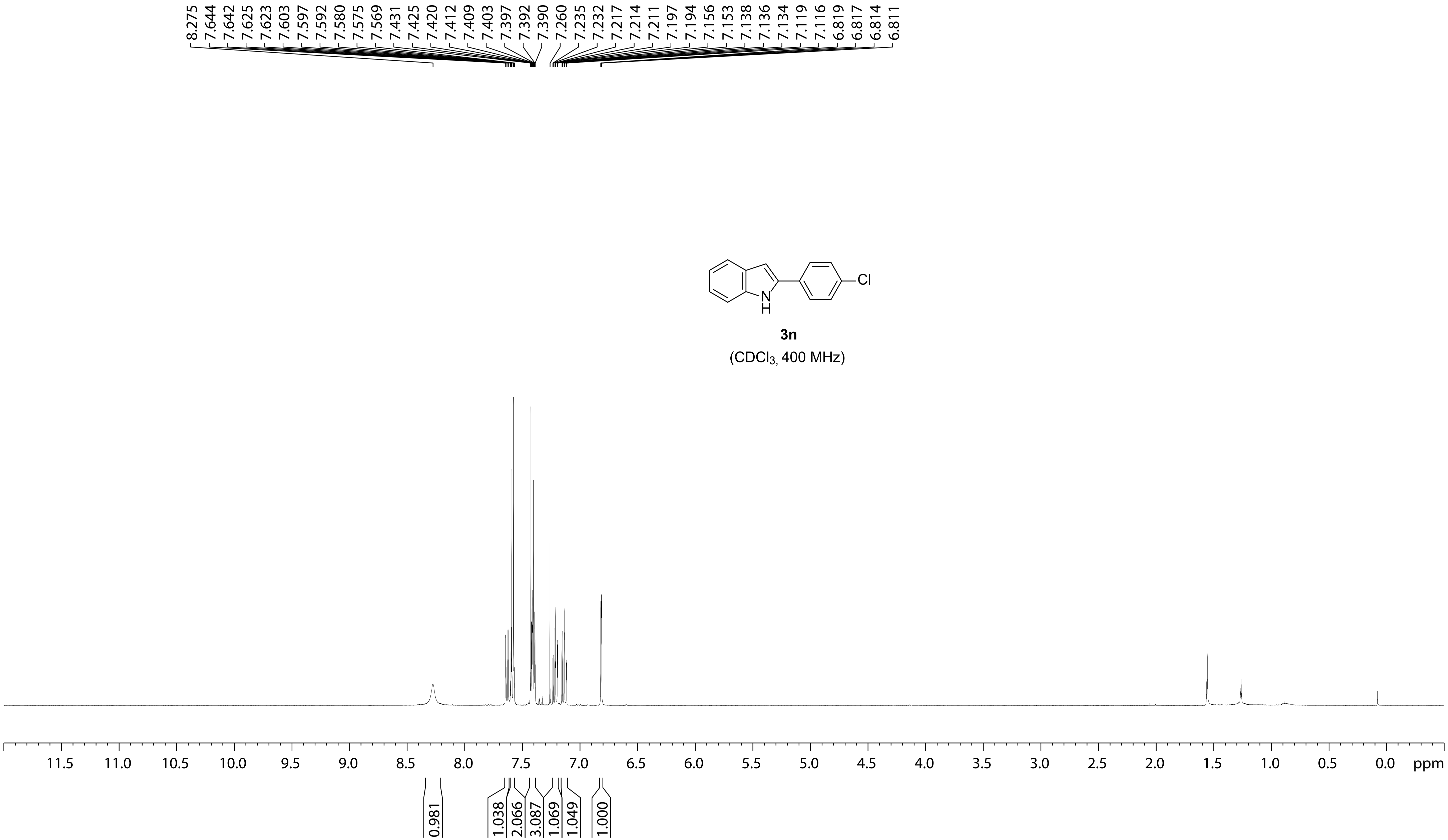
8.275
7.644
7.642
7.625
7.623
7.603
7.597
7.592
7.580
7.575
7.569
7.431
7.425
7.420
7.412
7.409
7.403
7.397
7.392
7.390
7.260
7.235
7.232
7.217
7.214
7.211
7.197
7.194
7.156
7.153
7.138
7.136
7.134
7.119
7.116
6.819
6.817
6.814
6.811
Cl
N
H
3n
(CDCl$_3$, 400 MHz)
11.5 11.0 10.5 10.0 9.5 9.0 8.5 8.0 7.5 7.0 6.5 6.0 5.5 5.0 4.5 4.0 3.5 3.0 2.5 2.0 1.5 1.0 0.5 0.0 ppm
0.981
1.038
2.066
3.087
1.069
1.049
1.000

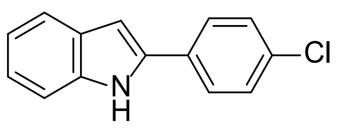

3n

(CDCl$_3$, 100.6 MHz)

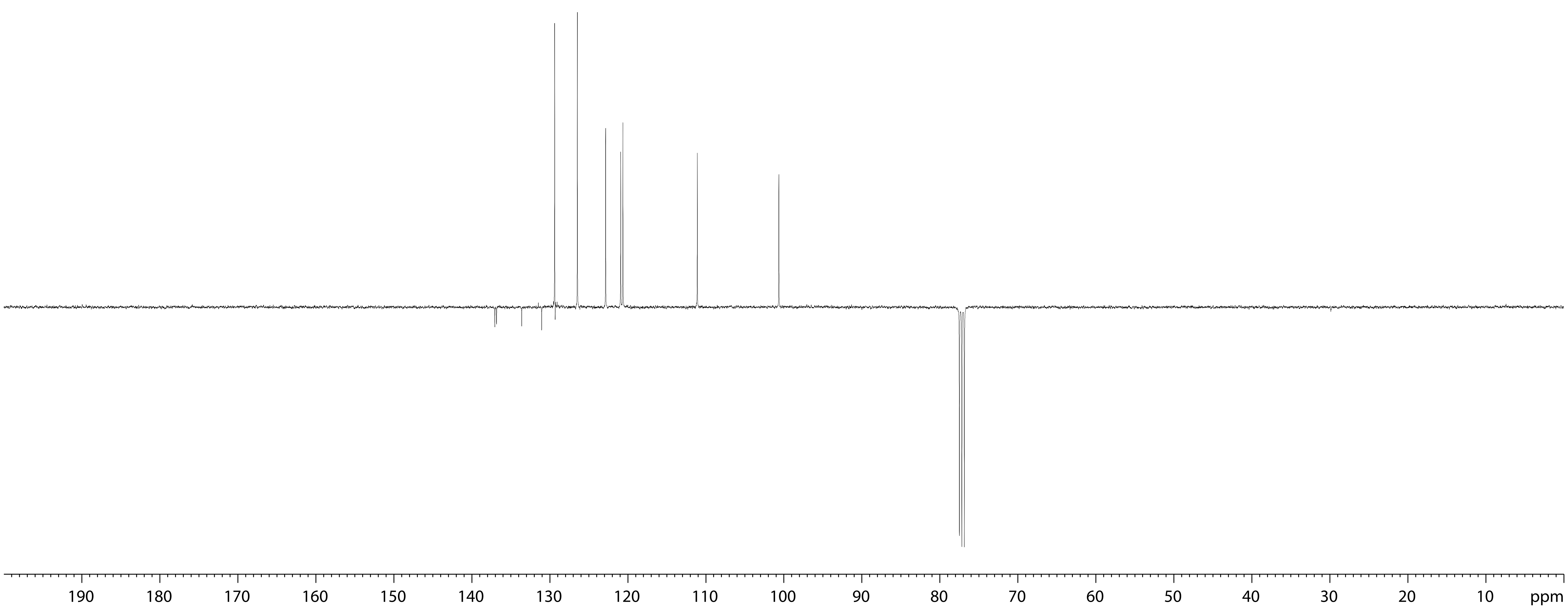

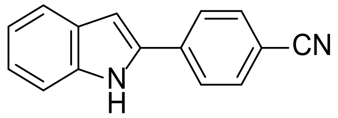

3o

($CDCl_3$, 400 MHz)

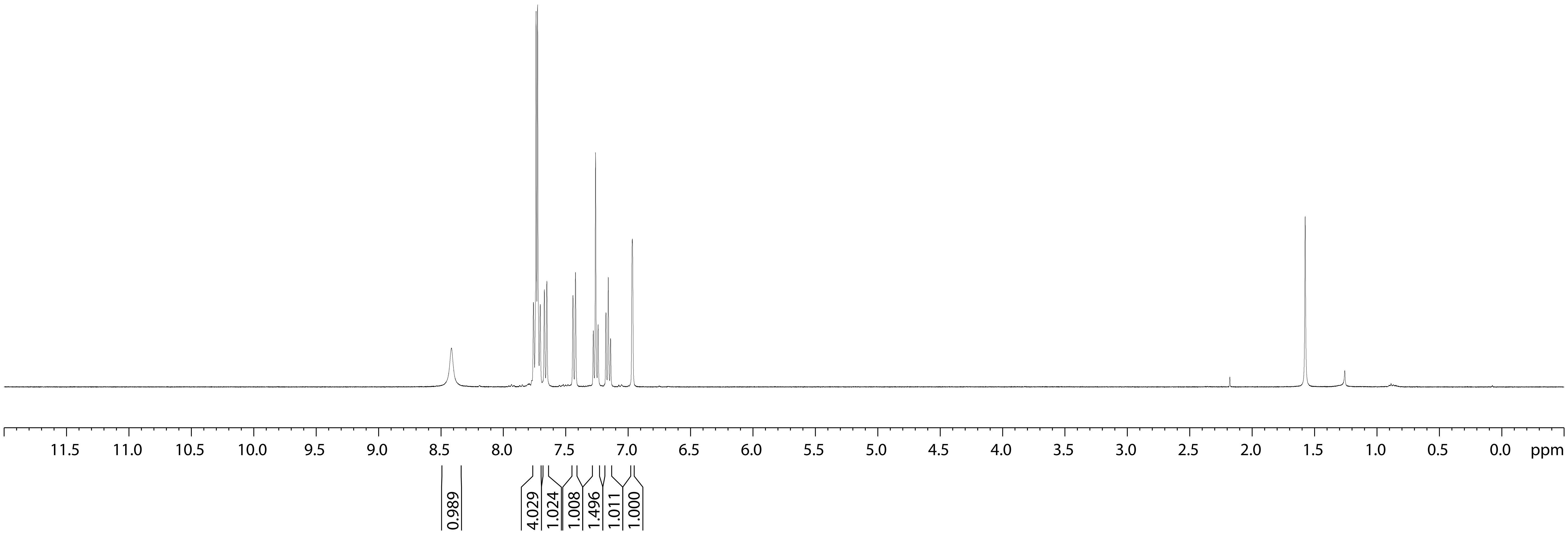

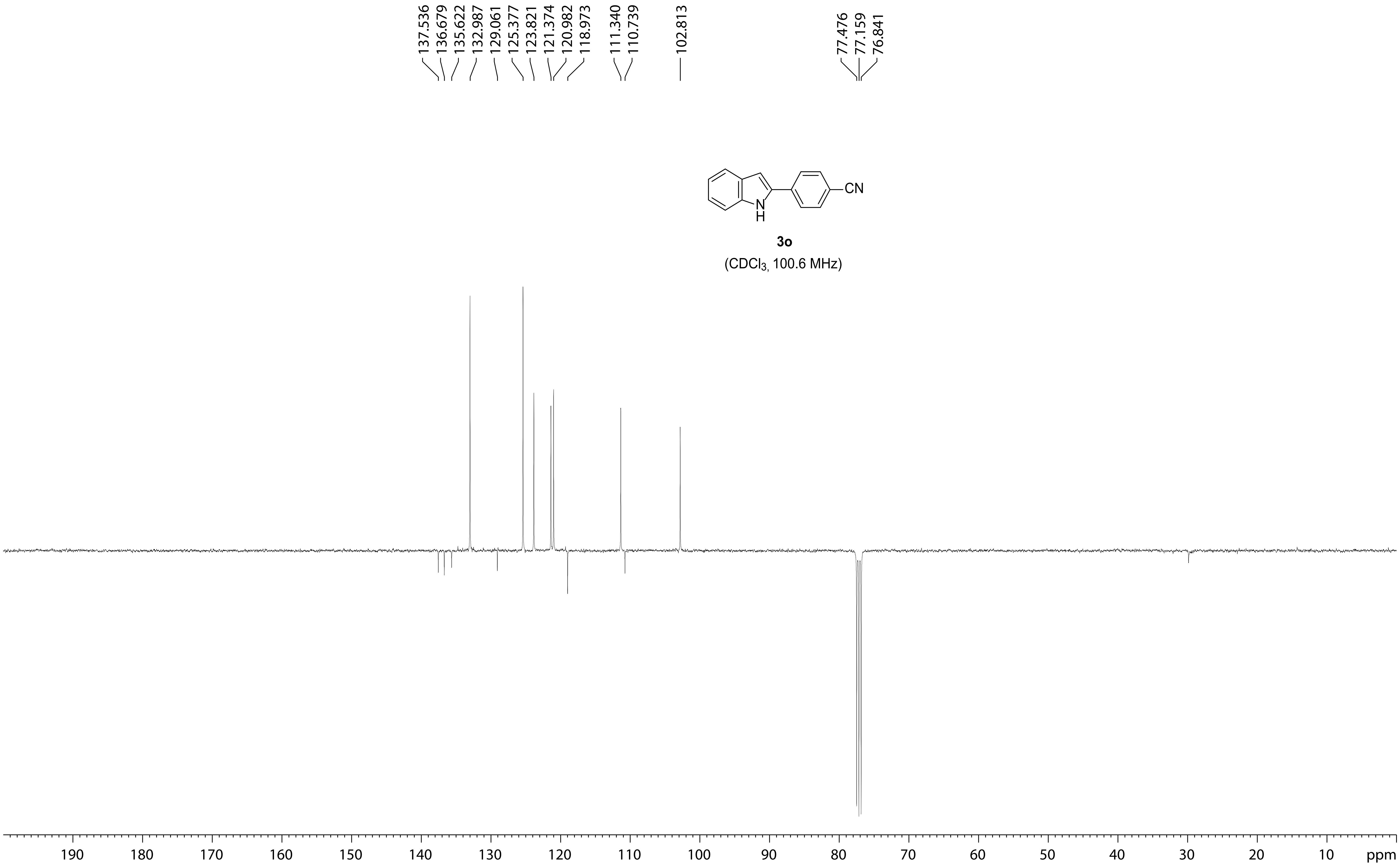
137.536
136.679
135.622
132.987
129.061
125.377
123.821
121.374
120.982
118.973
111.340
110.739
102.813
77.476
77.159
76.841
CN
N
H
3o
(CDCl3, 100.6 MHz)
190 180 170 160 150 140 130 120 110 100 90 80 70 60 50 40 30 20 10 ppm

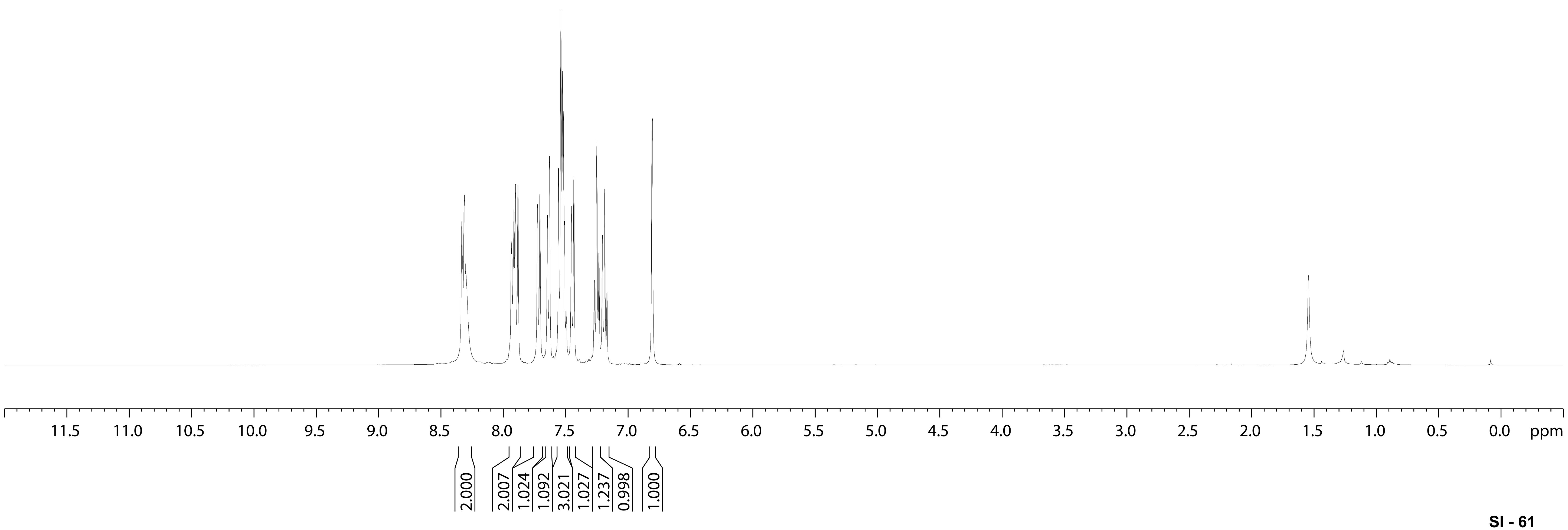

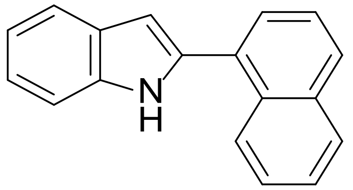

3p

($CDCl_3$, 400 MHz)

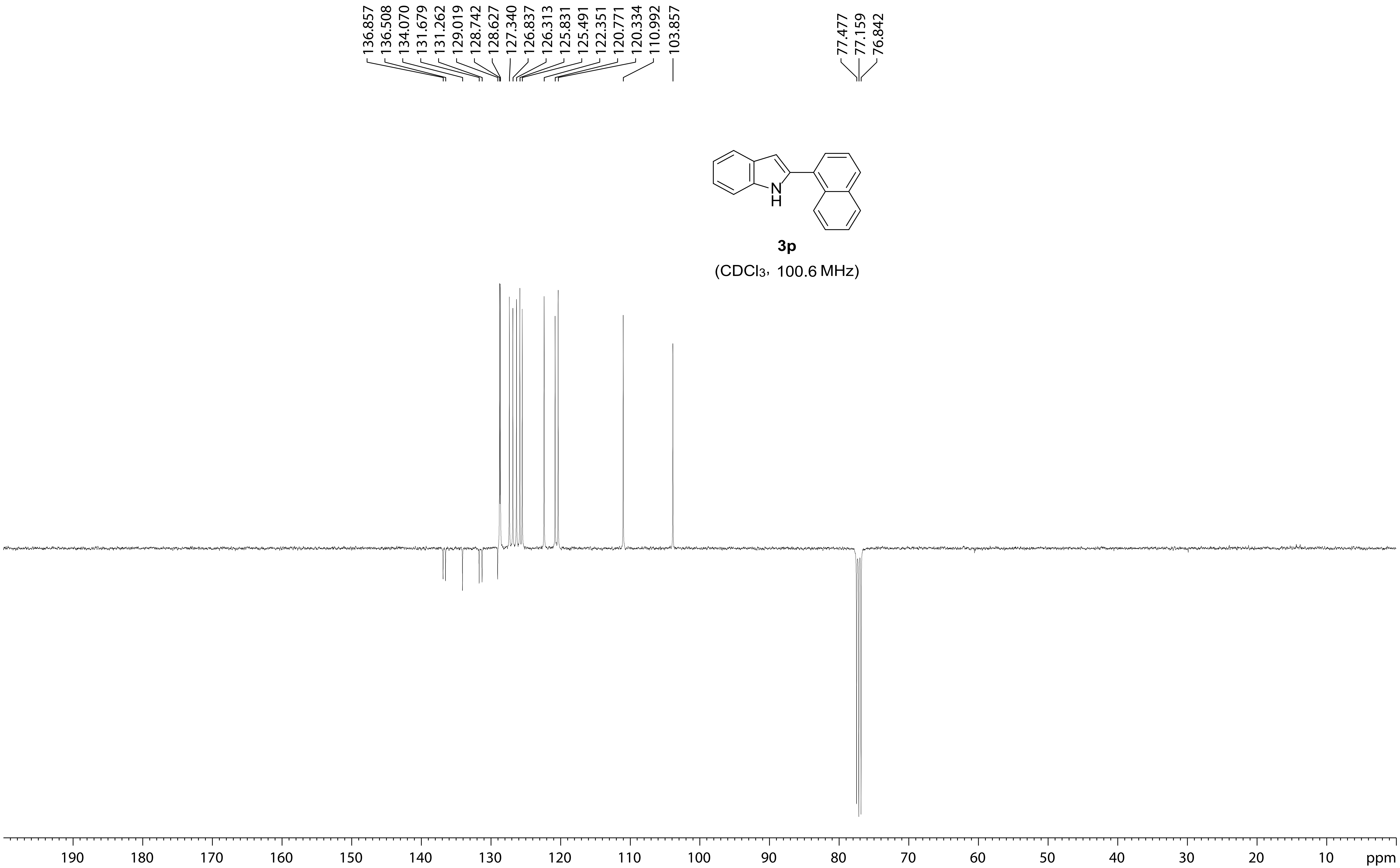
136.857
136.508
134.070
131.679
131.262
129.019
128.742
128.627
127.340
126.837
126.313
125.831
125.491
122.351
120.771
120.334
110.992
103.857
77.477
77.159
76.842
N
H
3p
(CDCl3, 100.6 MHz)
190
180
170
160
150
140
130
120
110
100
90
80
70
60
50
40
30
20
10
ppm

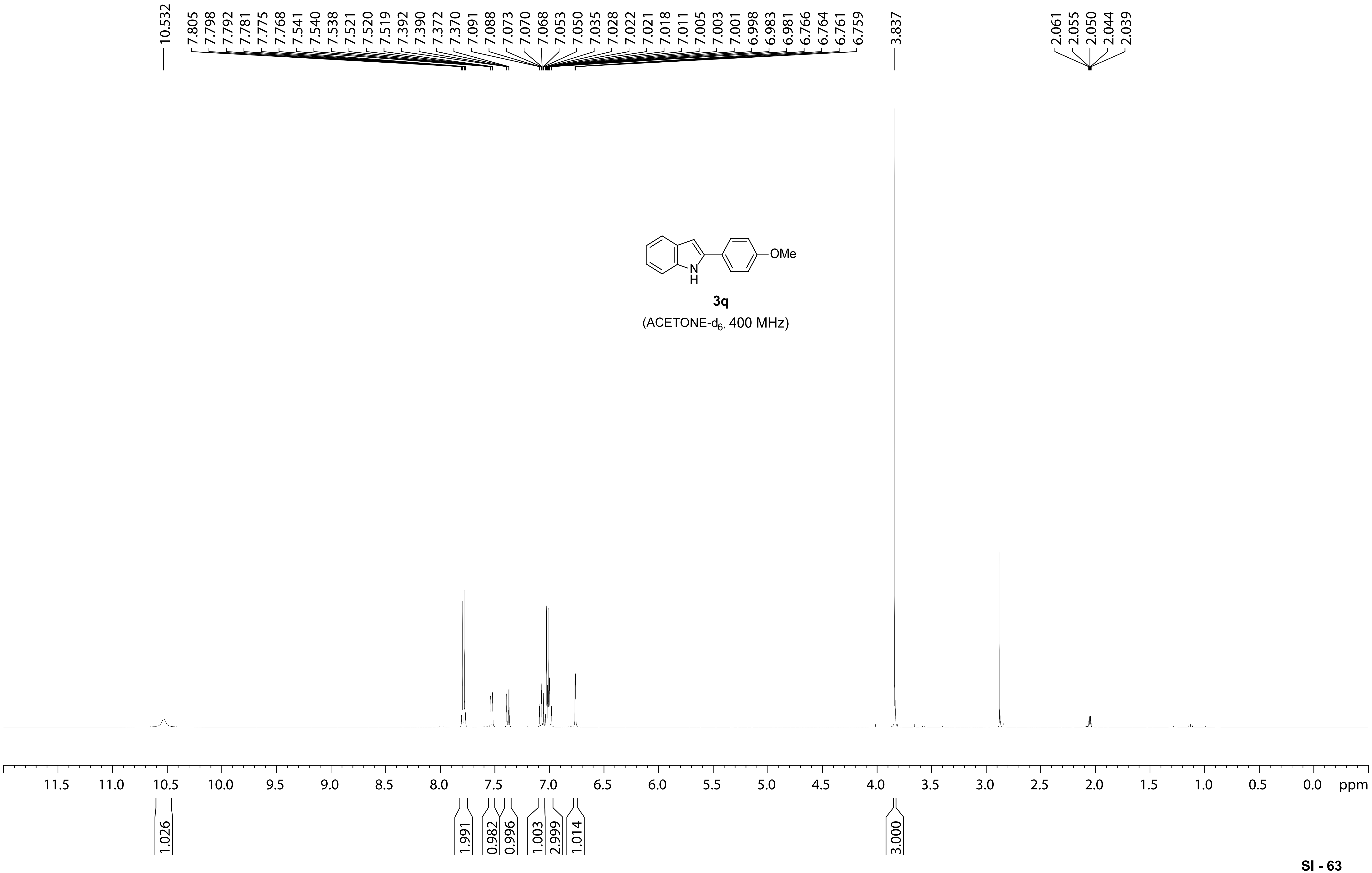
10.532
7.805 7.798 7.792 7.781 7.775 7.768 7.541 7.540 7.538 7.521 7.520 7.519 7.392 7.390 7.372 7.370 7.091 7.088 7.073 7.070 7.068 7.053 7.050 7.035 7.028 7.022 7.021 7.018 7.011 7.005 7.003 7.001 6.998 6.983 6.981 6.766 6.764 6.761 6.759
3.837
2.061 2.055 2.050 2.044 2.039
OMe
N
H
3q
(ACETONE-d$_6$, 400 MHz)
11.5 11.0 10.5 10.0 9.5 9.0 8.5 8.0 7.5 7.0 6.5 6.0 5.5 5.0 4.5 4.0 3.5 3.0 2.5 2.0 1.5 1.0 0.5 0.0 ppm
1.026 1.991 0.982 0.996 1.003 2.999 1.014 3.000

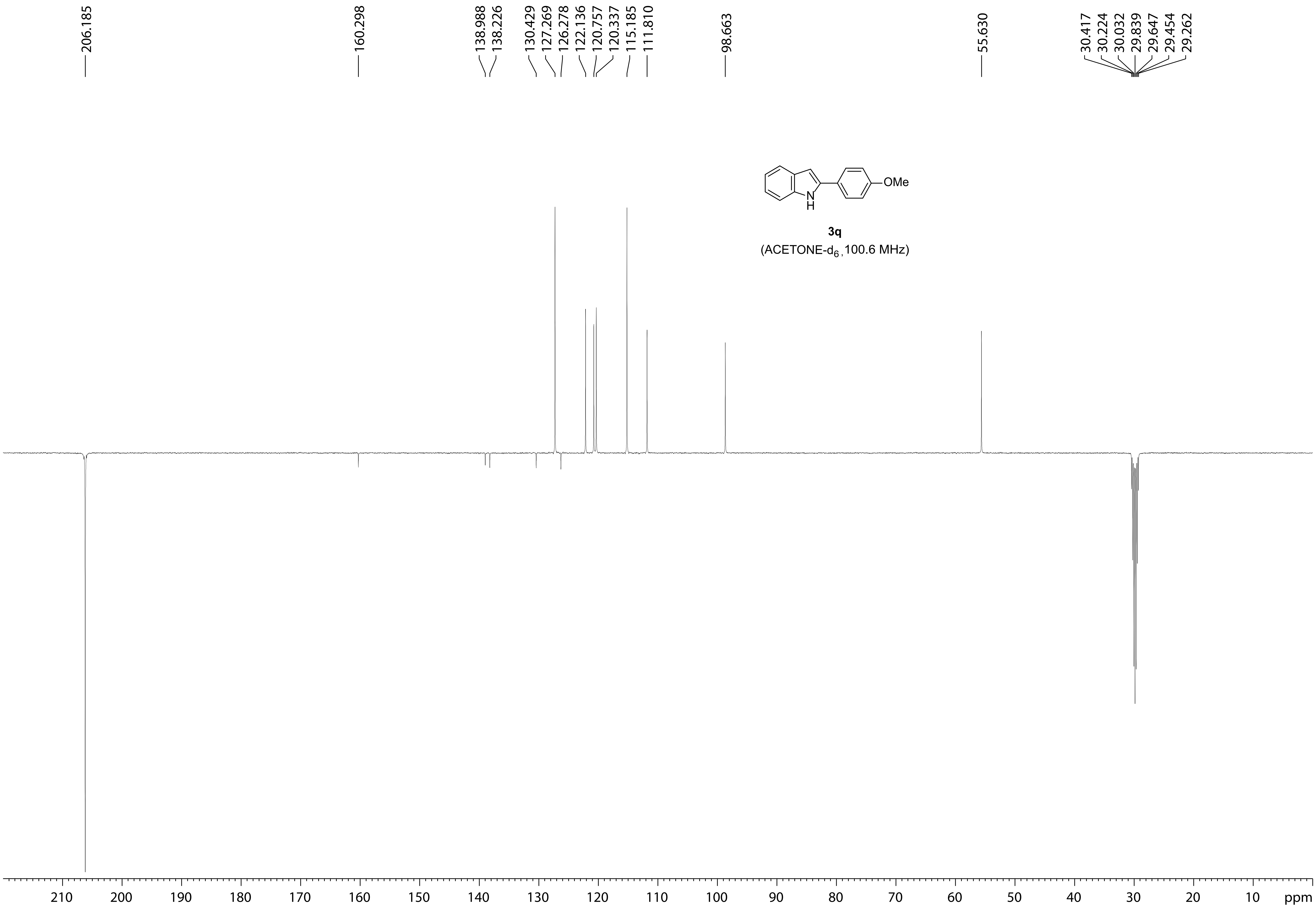
206.185
160.298
138.988
138.226
130.429
127.269
126.278
122.136
120.757
120.337
115.185
111.810
98.663
55.630
30.417
30.224
30.032
29.839
29.647
29.454
29.262
OMe
N
H
3q
(ACETONE-d6, 100.6 MHz)
210 200 190 180 170 160 150 140 130 120 110 100 90 80 70 60 50 40 30 20 10 ppm

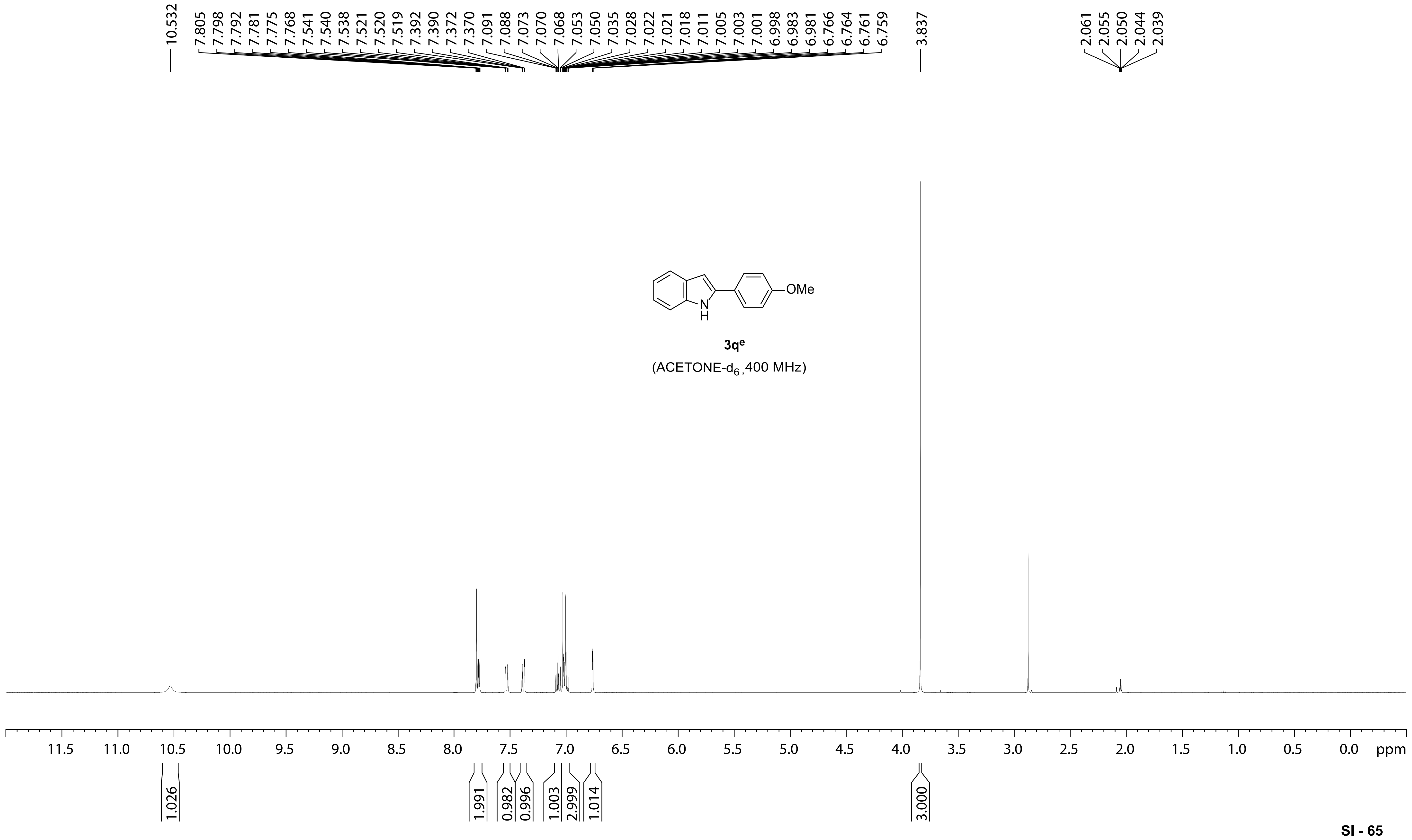

3q[e]

(ACETONE-d_6,400 MHz)

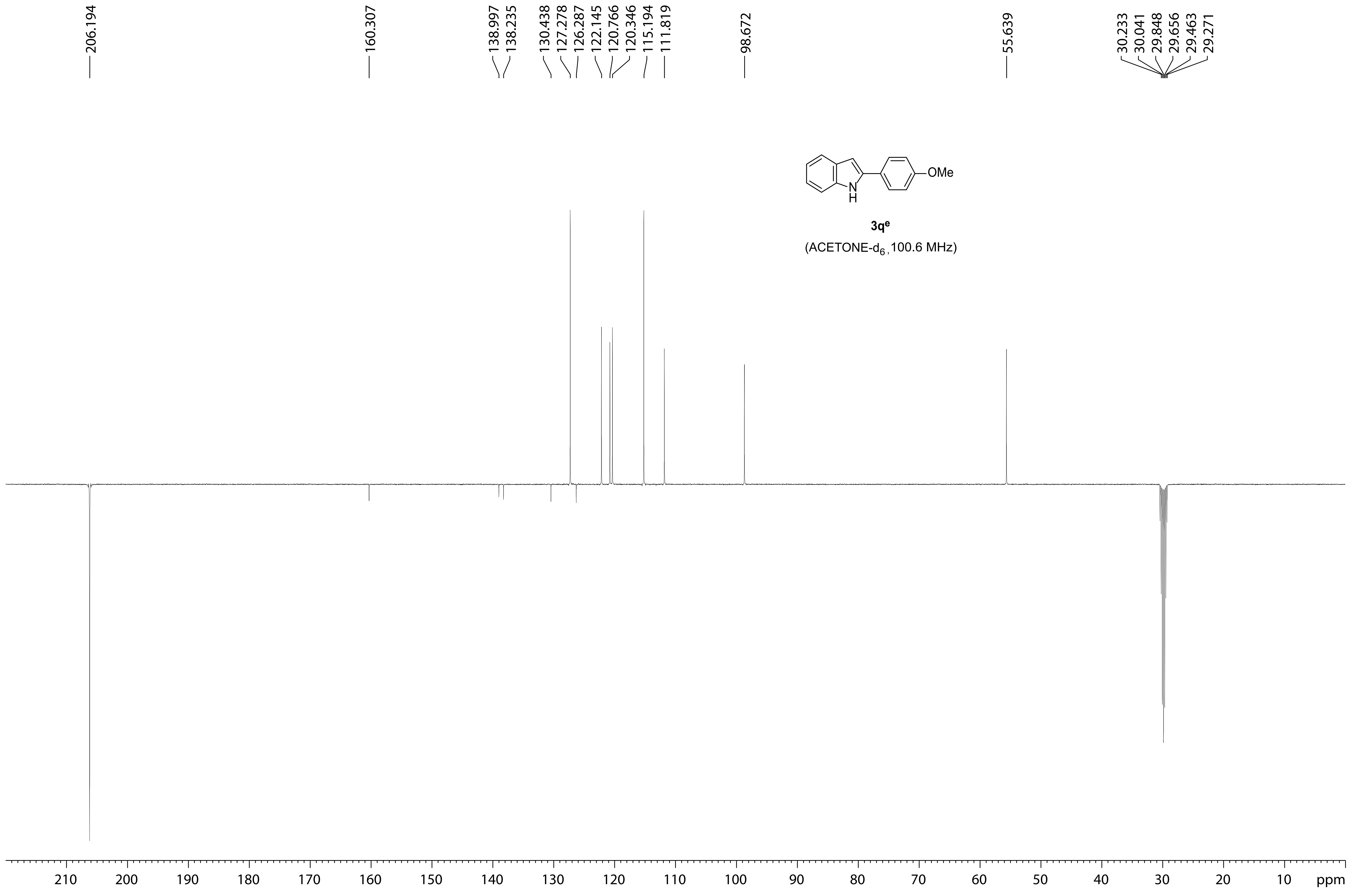
206.194
160.307
138.997
138.235
130.438
127.278
126.287
122.145
120.766
120.346
115.194
111.819
98.672
55.639
30.233
30.041
29.848
29.656
29.463
29.271
OMe
N
H
3q[e]
(ACETONE-d_6, 100.6 MHz)
210 200 190 180 170 160 150 140 130 120 110 100 90 80 70 60 50 40 30 20 10 ppm

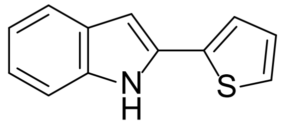

3r
($CDCl_3$, 400 MHz)

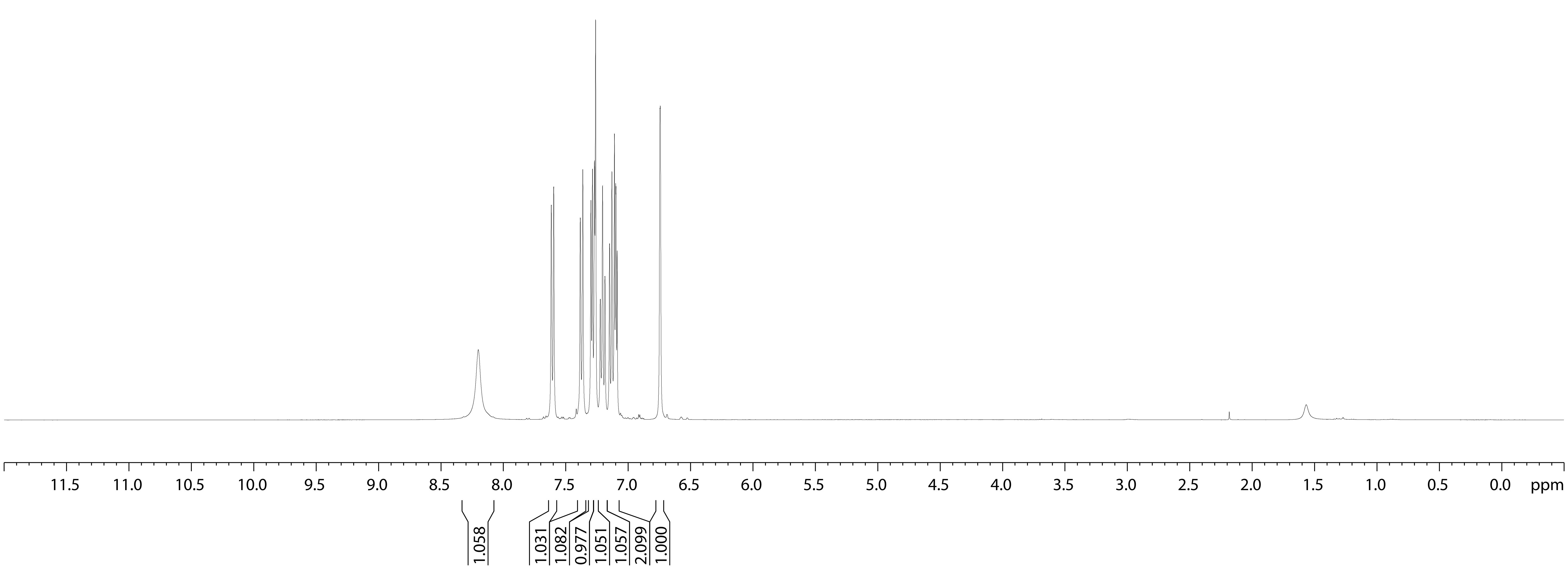

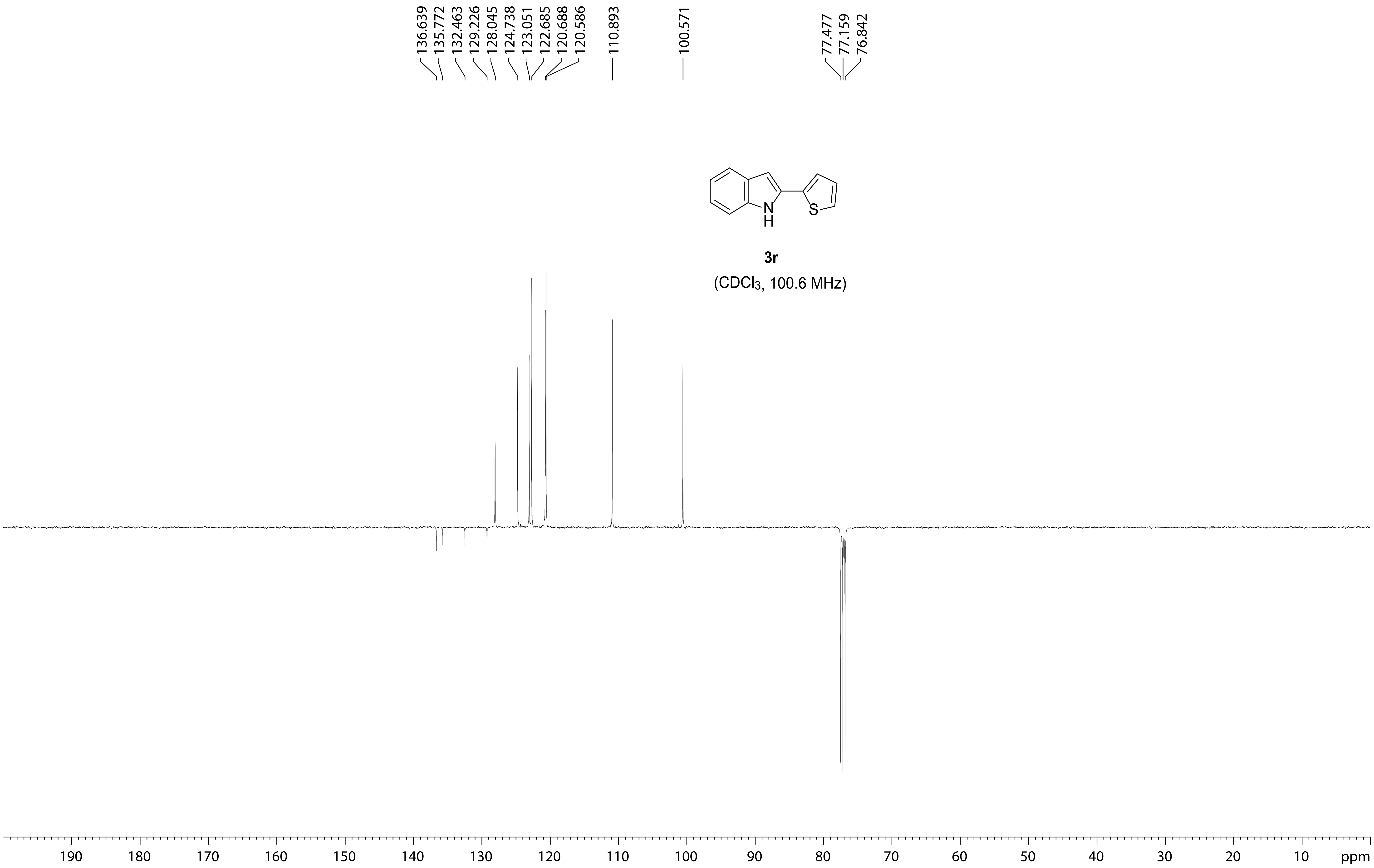
136.639
135.772
132.463
129.226
128.045
124.738
123.051
122.685
120.688
120.586
110.893
100.571
77.477
77.159
76.842
N
H
S
3r
(CDCl3, 100.6 MHz)
190 180 170 160 150 140 130 120 110 100 90 80 70 60 50 40 30 20 10 ppm

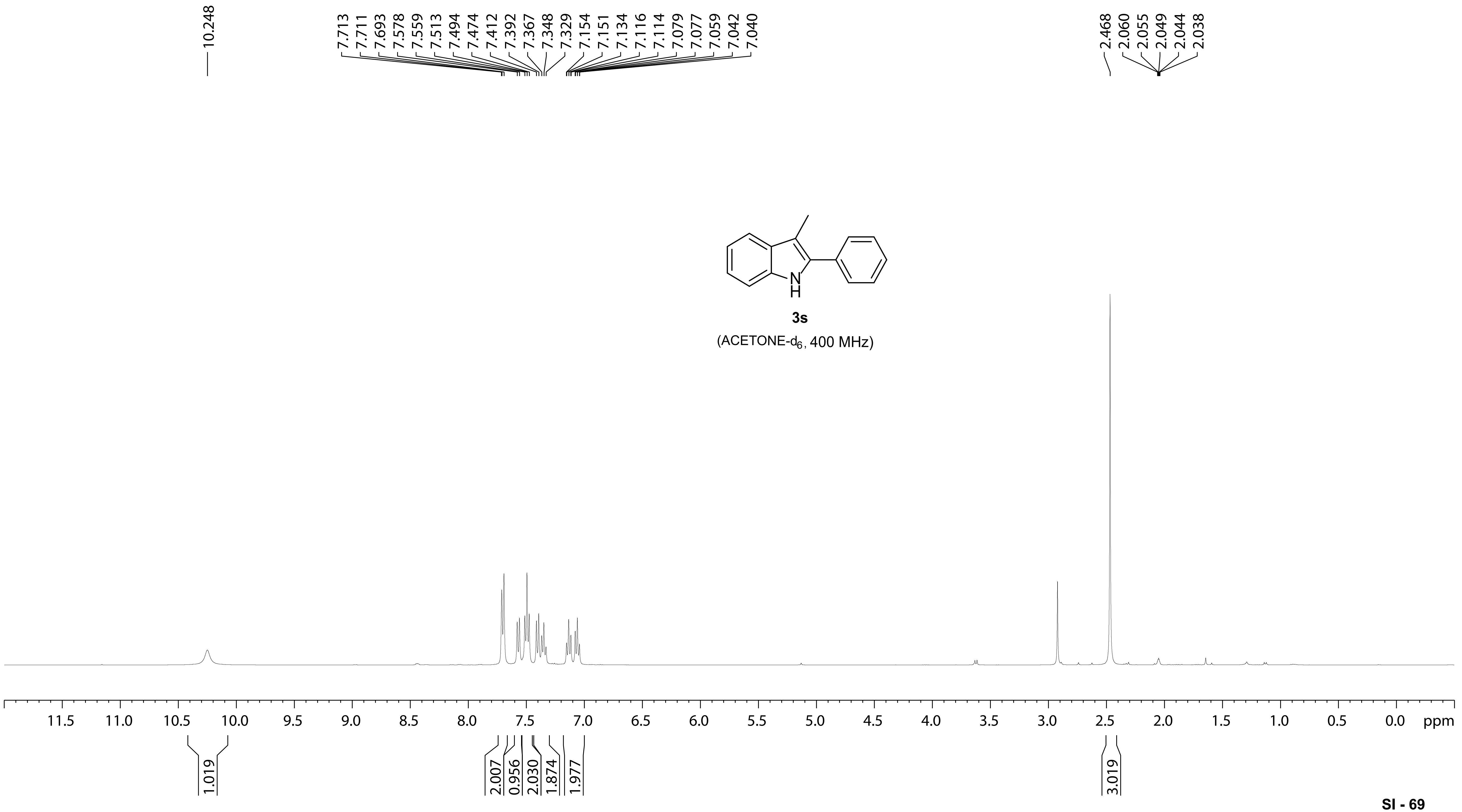
10.248
7.713 7.711 7.693 7.578 7.559 7.513 7.494 7.474 7.412 7.392 7.367 7.348 7.329 7.154 7.151 7.134 7.116 7.114 7.079 7.077 7.059 7.042 7.040
2.468 2.060 2.055 2.049 2.044 2.038
N
H
3s
(ACETONE-d_6, 400 MHz)
11.5 11.0 10.5 10.0 9.5 9.0 8.5 8.0 7.5 7.0 6.5 6.0 5.5 5.0 4.5 4.0 3.5 3.0 2.5 2.0 1.5 1.0 0.5 0.0 ppm
1.019
2.007 0.956 2.030 1.874 1.977
3.019

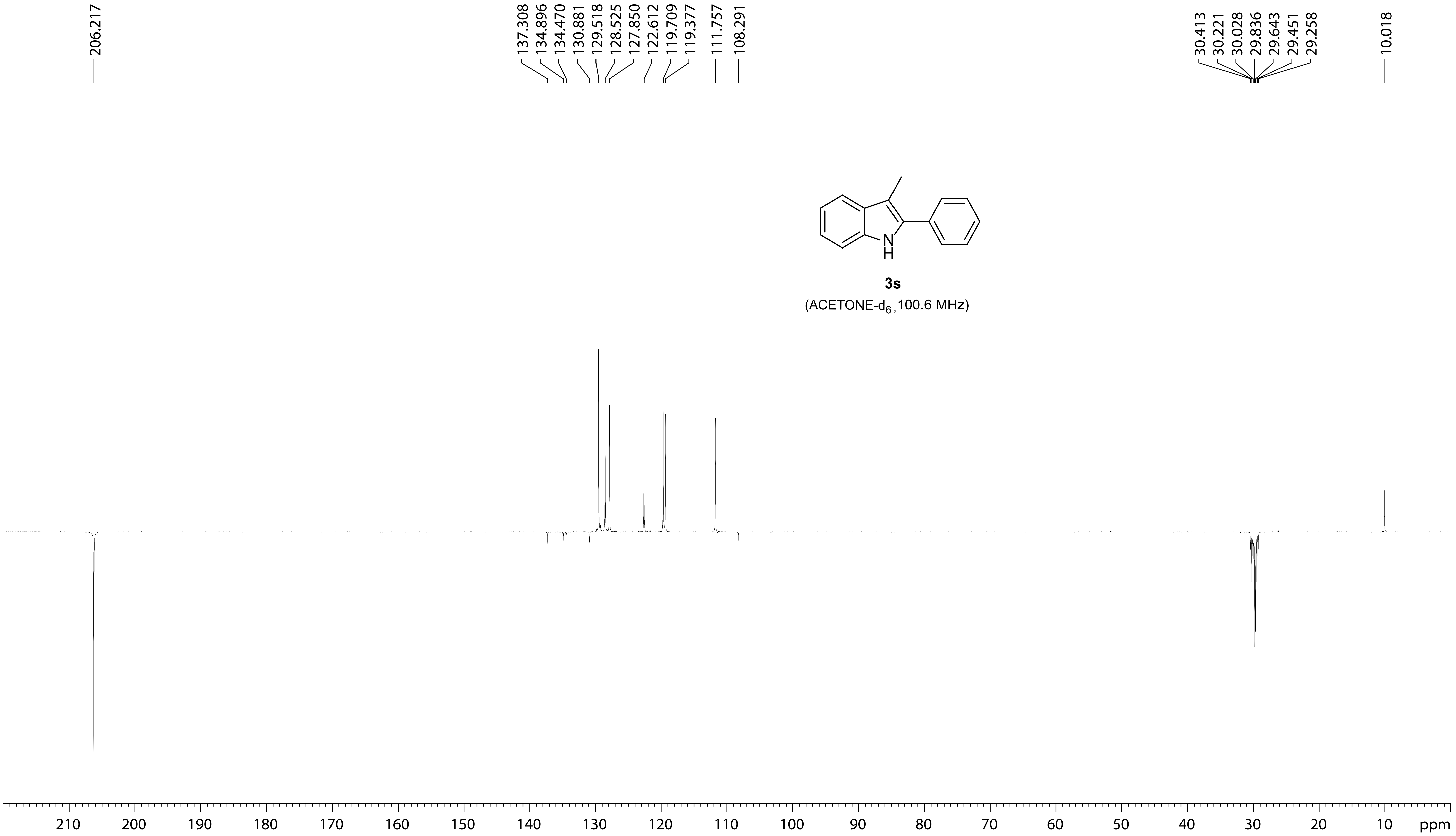

3s

(ACETONE-d_6, 100.6 MHz)
